# Alkynyl Halo-Aza-Prins Annulative Couplings

**DOI:** 10.1021/acs.joc.3c01305

**Published:** 2023-11-16

**Authors:** Jackson J. Hernandez, Alexandra P. Lawrie, Alison J. Frontier

**Affiliations:** Department of Chemistry, University of Rochester, 120 Trustee Road, Rochester, New York 14611, United States

## Abstract

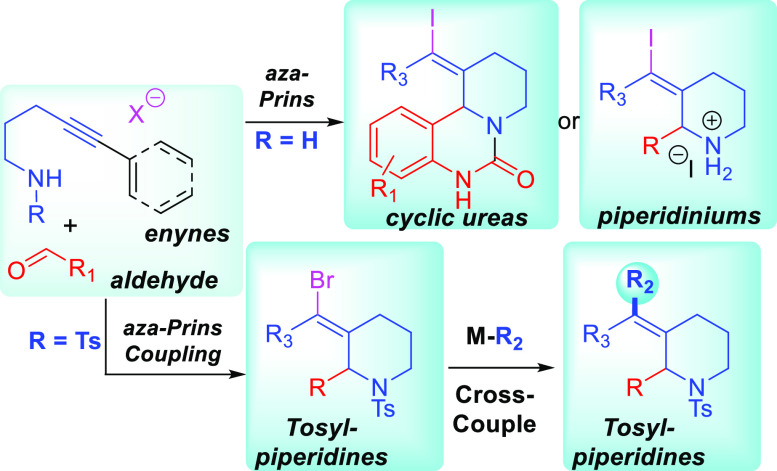

This article is a comprehensive report describing our studies in the field of aza-alkynyl Prins chemistry, comparing and contrasting the different reaction partners and reactivities observed during method development. The synthetic strategies combine an alkynyl aza-Prins coupling with an annulation, enabling the preparation of different nitrogen-containing heterocycles. Different iminium ions are explored as viable electrophiles for an alkynyl Prins cyclization, terminated by capture with a halogen nucleophile to form a vinyl halide. The synthetic utility of this functional handle is exploited through a number of Suzuki cross-couplings, allowing for the preparation of a modest library of compounds. In most cases, the Prins couplings are highly selective for the vinyl halides with *E* geometry, resulting from anti-addition across the alkyne.

## Introduction

**Scheme 1:**
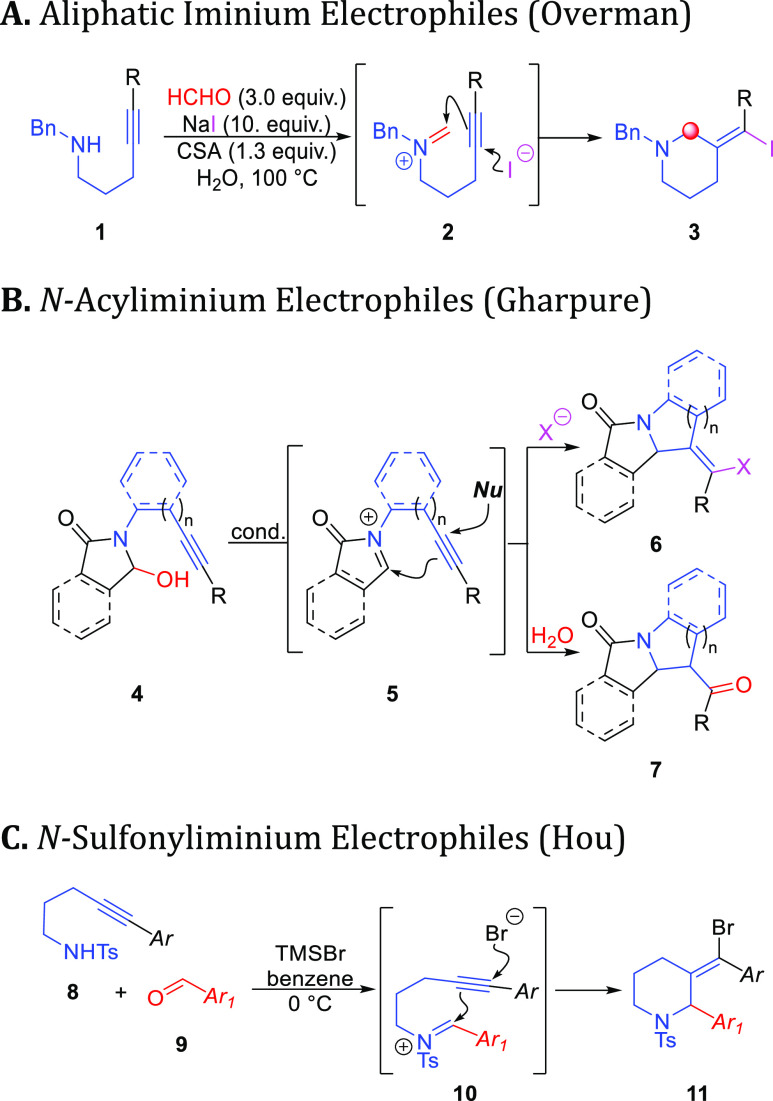
Alkynyl Halo-Aza-Prins Reaction

The alkynyl aza-Prins cyclization allows for the rapid generation of molecular complexity from simple starting materials.^1^ A wide range of methods have been developed to promote the efficient Prins reaction of alkynes with different reaction partners. Alkyl amines (Scheme 1A),^2^ alpha-cyano-amines,^3^ hemiaminals (Scheme 1B),^4−10^ and sulfonamides (Scheme 1C).^17−22^ have all been employed as iminium ion precursors.

The Overman group has spearheaded research in the field of the alkynyl aza-Prins cyclization of secondary or tertiary alkyl-amines.^2,3^ These reactions are often difficult due to the basic nature of the electron-rich nitrogen center, which explains the harsh conditions needed for cyclization. Scheme 1A shows an example of these reactions with a secondary benzylamine **1** in the presence of camphorsulfonic acid (CSA) or tosylic acid (TsOH), and an excess of sodium iodide (NaI), to afford adducts like **3** via iminium **2**.^2^

A number of reports have also described cyclizations of alkynes and *N*-acyliminium ion electrophiles. These often start with hemiaminals like **4**, which can be dehydrated to form the electrophilic *N*-acyliminium intermediate **5**. The subsequent alkynyl Prins cyclization then gives products like **6** in the presence of a halide nucleophile,^4−7^ or **7** upon hydrolysis^8−10^ (Scheme 1B).

*N*-Sulfonyl iminium ions are the last class of electrophiles that have been employed in alkynyl aza-Prins chemistry. In these cyclizations, sulfonamide reactants are leveraged for the preparation of functionalized pyrazoles,^11^ indoles,^12^ indolines,^13^ and piperidines.^14,15^ Recently, a report by Hou et al. shows the use of bromotrimethylsilane (TMSBr) to promote the reaction of a sulfonamide **8** and a carbonyl coupling partner **9** to make tosyl-piperidines like **11** via *N*-tosyl iminium intermediate **10** (Scheme 1C).

In this paper, we provide a comprehensive report of our work in the field of alkynyl aza-Prins cyclization.^16^ These studies were informed by our previous work on reaction cascades initiated by analogous alkynyl *oxa*-Prins cyclizations.^17,18^ Specifically, we have developed the first aza-Prins coupling of primary amines with benzaldehyde derivatives for the synthesis of functionalized quinazolinones, via an *N*-acyliminium intermediate. We also showcase conditions for the alkynyl aza-Prins cyclization of a phthalimide-derived *N*-acyliminium electrophile, allowing for the synthesis of functionalized isoindolones. Finally, we explore the coupling of aliphatic aldehydes with sulfonamides to generate *N*-tosyl iminiums capable of undergoing an alkynyl aza-Prins cyclization, resulting in the preparation of tosyl-piperidines. Through comparison of these different cases, we offer insights related to reactivity and conditions optimal for achieving the three types of cyclizations.

Regarding the specifics of the proposed mechanism for these cyclizations, the body of previous work on oxa- and aza-alkynyl Prins cyclizations offers plenty of data, but no consensus, on whether the reaction proceeds through a discrete vinyl cation intermediate (a stepwise process), or involves simultaneous cyclization and halide capture (a concerted process). In alkynyl aza-Prins literature in particular, a counterion effect is observed,^2,3,19^ which suggests a concerted pathway. The *E* isomer is expected in the concerted cyclization, through addition of halide to the alkyne *anti* to the Prins electrophile, and indeed the *E* geometric isomer is typically formed exclusively, or as the major isomer. However, in cases where *E*/*Z* mixtures are observed, a vinyl cation intermediate is implicated. In the schemes in this paper, we depict the concerted pathway for simplicity, but it must be stated that the stepwise vs concerted nature of the cyclizations has not been defined unambiguously.

## Results and Discussion

### Aza-Prins Coupling with Primary Amines and *o*-Formyl Carbamates

**Scheme 2:**
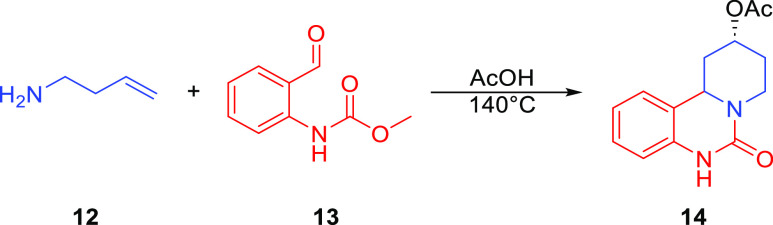
Aza-Prins Synthesis of 2-Quinazolines (Sawant)

In a report by Sawant and co-workers, conditions for the synthesis of 2-quinazolinones **14** from primary homoallylic amines **12** and benzaldehyde derivatives **13** were developed (Scheme 2).^20^ Inspired by this reaction, we wanted to explore the alkynyl Prins reaction in this context. To this end, homopropargylic amine **15a** was examined in this reaction (Table 1).

**Table 1:**
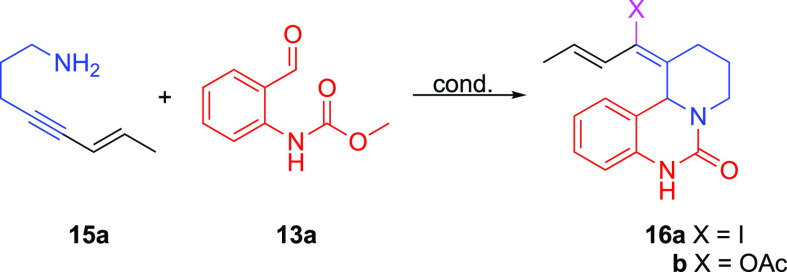
Optimization of Alkynyl Aza-Prins Reaction with Primary Amine **15a**

entry	solvent	promoter (equiv)	*T* (°C)	prod. (% *y*)
1	AcOH	solvent	120	**16b** (20)^a^
2^b^	EtOH	AcOH (15)	70	**16a** (11)
3^c^	MeCN	AcOH (15)	80	**16a** (60), **16b** (8)
4^d^	MeCN	AcOH (15)	80	**16a** (81)

a **16b** isolated in a 3.5:2.2:1.3:1 *E*/*Z* isomer ratio.

b 2.0 equiv TBAI used.

c 3.0 equiv TBAI used.

d 10 equiv of NaI used.

Subjecting amine **15a** and aldehyde **13a** to Sawant’s conditions produces vinyl acetate **16b** in 20% yield (Table 1, entry 1). While switching solvents to ethanol and adding tetrabutyl ammonium iodide (TBAI; 2–3 equiv) as a halide source affords **16a** in 11% yield (entry 2), the cyclization yield increases to 68% (**16a** + **16b**) upon heating to 80 °C in acetonitrile (MeCN; entry 3). Replacing the TBAI with 10 equiv of sodium iodide, which is a cheaper and greener alternative, gives **16a** in 81% yield (entry 4). Cyclizations using other halide sources (Et_4_NBr, LiCl, and LiF) are less efficient, with low yields and complex mixtures observed. Overall, iodide sources give the best results, and we settled on NaI as the halide source for the rest of the study. Fifteen equivalents of AcOH give optimal yields of the vinyl halide product **16a** and minimize the formation of undesired vinyl acetate **16b**, which becomes more favored at higher acetic acid concentrations. When the amount of acetic acid is reduced, reactions become sluggish and take over 24 h to go to completion.

**Scheme 3:**
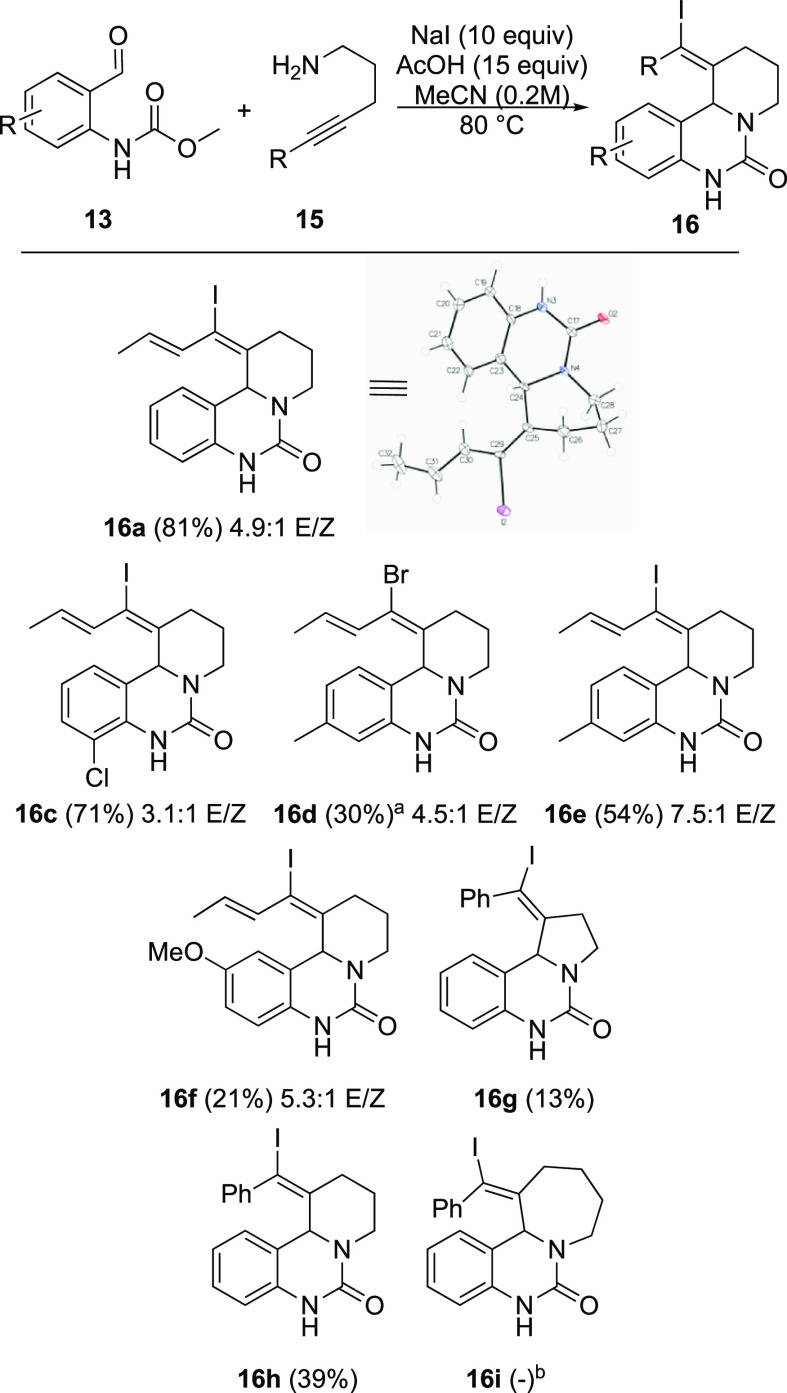
Scope of Alkynyl Aza-Prins Cyclizations 10 equiv of NaBr used instead of NaI. Aza-Prins adduct was not observed.

With these optimized conditions, a few experiments exploring the scope were performed (Scheme 3). As seen in the optimization Table 1, the reaction of benzaldehyde **13a** and amine **15a** results in the formation of aza-Prins adduct **16a** in 81% yield. A 3-chlorobenzaldehyde produces **16c** in good yield. As expected, a comparison of the results for **16d** and **16e** shows that better results are obtained with sodium iodide, relative to sodium bromide. Not surprisingly, a drop in yield is observed in the formation of **16f**, in which the carbamate is deactivated by the methoxy substituent. The enyne **15a** employed for the synthesis of **16a**–**f** has a 4.6:1 *E*/*Z* isomer ratio, and both isomers engage in the Prins cyclizations. The *E*/*Z* ratios of **16a**-**f** range from 3.1:1 to 7.5:1, depending upon the degree of isomerization during the cyclization, and whether the isomers can be separated during purification. The pyrrolidine adduct **16g** is produced in only 13% yield. The arenyne reactant **15c** engages to afford a modest yield of **16h**. The corresponding azepine adduct **16i** is not observed at all, amid a complex mixture of undesired products.

**Scheme 4:**
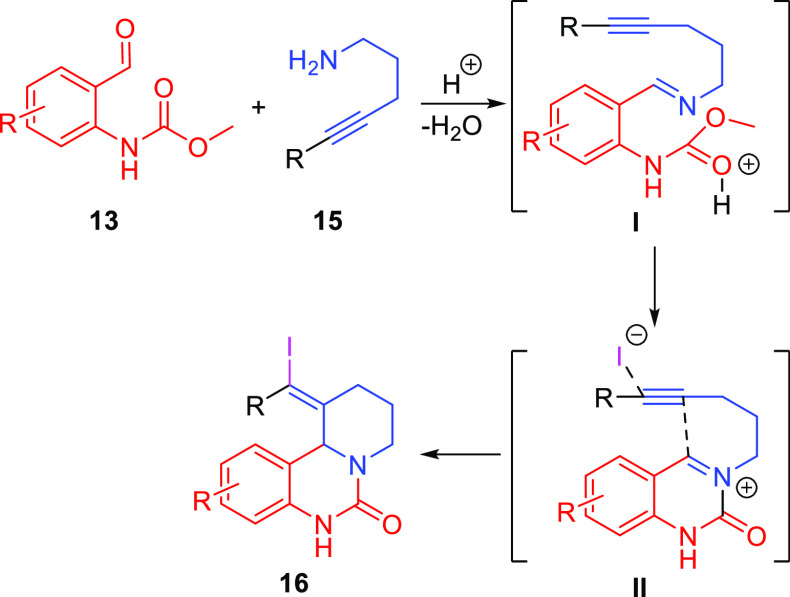
Mechanism for the Alkynyl Aza-Prins Coupling of Primary Amines and Benzaldehydes

The proposed mechanism for this reaction is shown in Scheme 4. Condensation of amine **15** and aldehyde in **13** generates imine **I**. The imine then adds to the carbamate group, releasing methanol and forming *N*-acyliminium **II**.^20,21^ Electrophile **II** then undergoes alkynyl Prins cyclization to form the final quinazolinone product **16**.

Continuing to explore primary enyne amine coupling partners, the isopropyl aldehyde reactant delivers ammonium salt **16j** using the optimized conditions in 92% crude yield (eq 1), although the product is unstable and decomposes upon isolation.
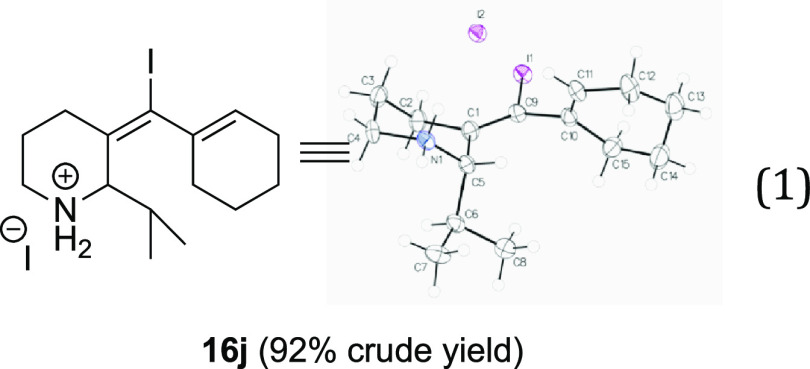
1

### Aza-Prins Cyclization of Phthalimide Derivatives

**Scheme 5:**
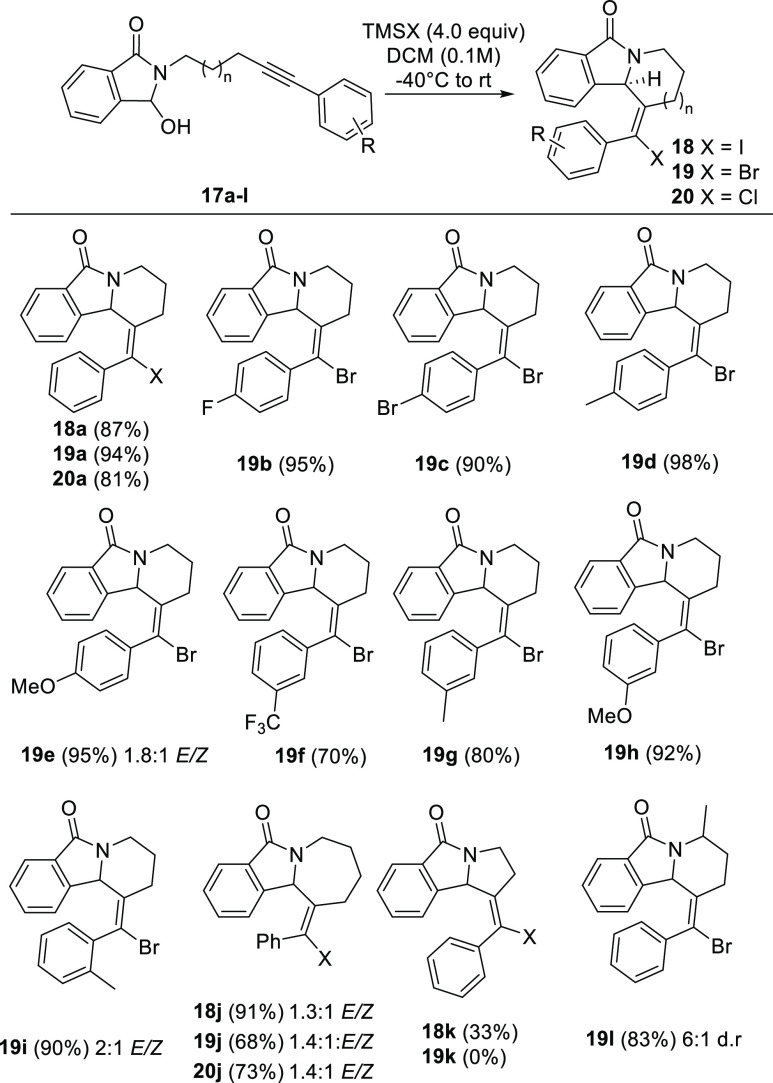
Alkynyl Aza-Prins Cyclization of Phthalimido Arenyne Hemiaminals^16^

The first study of alkynyl aza-Prins reactions conducted in our lab involved phthalimide-derived alkynes **17**.^16^ As shown in Scheme 5, halotrimethylsilane reagents work well for arenyne-derived substrates **17a**–**l**. Adducts **18**, **19**, and **20**, containing vinyl chloride, bromide, and iodide moieties, respectively, can all be synthesized in good yield. Six- and seven-membered rings form smoothly, while five-membered rings do not. Alkyne **19l**, with a methyl substituent at the carbon next to the nitrogen, cyclizes to afford **19l** with moderate diastereoselectivity (6:1 dr). The *E* isomer is formed exclusively in cyclizations that form six-membered rings unless the system is unusually hindered or electron-releasing (**19e** and **19i**). Seven-membered rings are obtained as *E*/*Z* mixtures. Both steric and electronic factors seem to influence the selectivity.

**Scheme 6:**
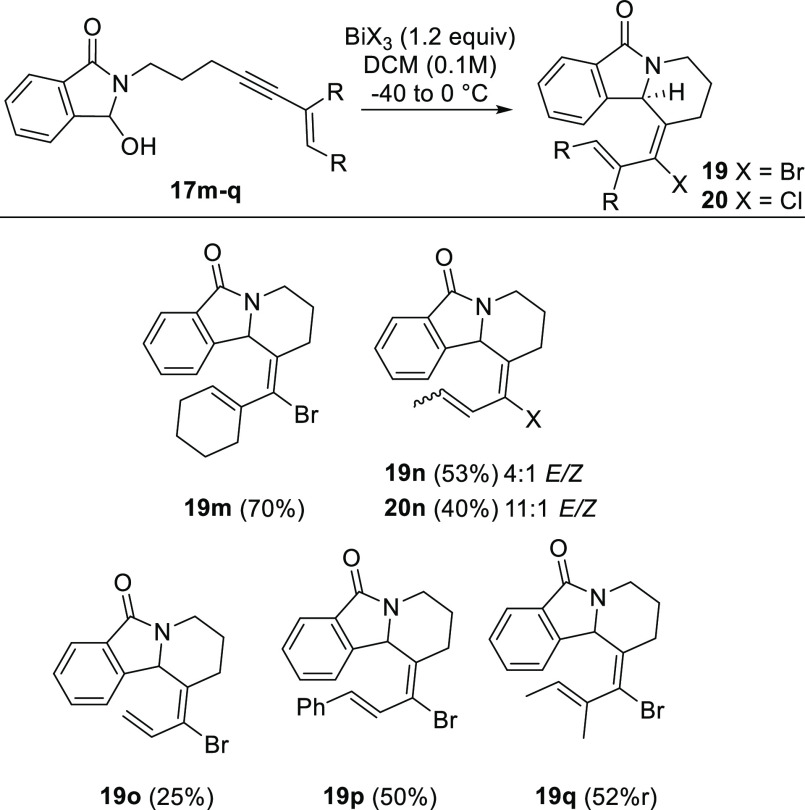
Alkynyl Aza-Prins Cyclization of Phthalimido Enyne Hemiaminals^16^

Alongside this cyclization study of arenyne-linked phthalimide derivatives **17a**–**l**, we evaluated enyne-linked reactants **17m**–**q**. In contrast to the arenynes, Bi(III) halide salts are optimal promoters for the enyne cyclizations.^22^ Yields are higher for di- and trisubstituted alkenes, compared to the terminal enyne **19o** (Scheme 6).

**Scheme 7:**
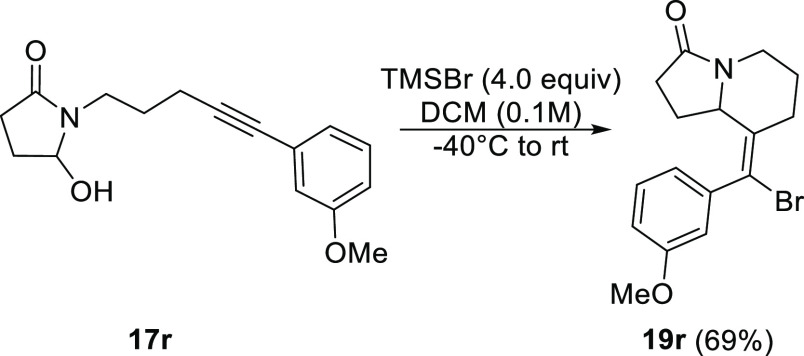
Alkynyl Aza-Prins Cyclization of a Succinimide-Derived Hemiaminal^16^

Finally, we tested cyclization onto the *N*-acyliminium ion intermediate generated from succinimide-derived reactant **17r**. Scheme 7 shows that the developed conditions delivered **19r** in good yield.

### Alkynyl Aza-Prins Annulation with Sulfonamides

In order to develop methods complementary to our previous work on alkynyl oxa-Prins cyclizations,^17^ we chose to target *N*-sulfonyl iminium electrophiles as intermediates in the intramolecular alkynyl aza-Prins reaction. Table 2 summarizes some of our efforts for optimizing the aza-Prins cyclization.

**Table 2:**
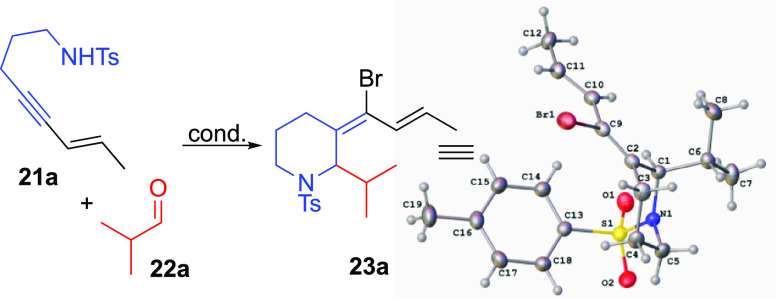
Optimization of Alkynyl Aza-Prins Reaction with Sulfonamides

entry	solvent	promoter (equiv)	*T* (°C)	**23a** (% yield)
1^a^^,^^b^	MeCN	AcOH (15)	80	no rxn
2^b^^,^^c^	H_2_O	CSA (1.0)	100	decomp
3^d^	DCM	FeCl_3_ (0.15)	rt	26%^e^
4^a^	DCM	*p*-TSA (5.0)	rt	26%^e^
5	benzene	TMSBr (4.0)	0	59%
6	toluene	TMSBr (4.0)	–20	75%

a 4.0 equiv of TBAI used as halide source.

b Benzyl-protecting group used instead of tosyl.

c 10.0 equiv of NaI used as halide source.

d 1.0 equiv of TMSCl used as halide source.

e Complex mixture of products observed.

The conditions that were developed for primary amines (see Table 1) do not promote cyclization of the corresponding sulfonamides **21a** (entry 1). Using CSA in water, in the presence of a halide ion^2c^ leads to decomposition upon heating to 100 °C and no reaction at lower temperatures. Catalytic FeCl_3_ in the presence of TMSCl^14b^ gives the halo-aza-Prins product in low yields.

**Scheme 8:**
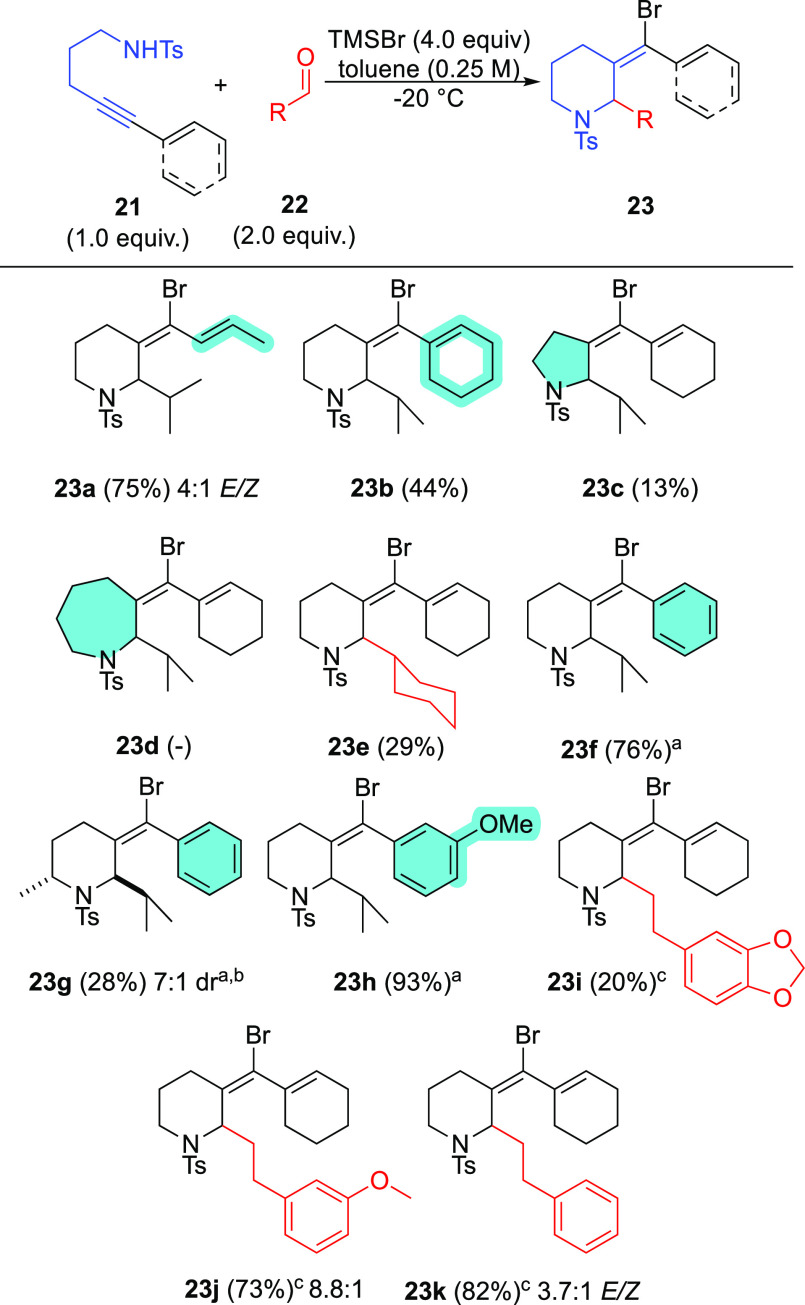
Scope of the Alkynyl Aza-Prins Annulation Reaction done at 0 °C. Reaction heated to 40 °C for 3 days. Reaction done at −40 °C.

As we were working on developing conditions for these cyclizations, Hou and co-workers reported using TMSBr in benzene to promote a similar aza-Prins reaction.^15^ Application of this TMSBr protocol delivers 59% of aza-Prins product **23a**. Switching solvents to toluene and keeping the reaction at −20 °C leads to an improved yield of **23a** (75%; Scheme 8).

Using these optimized conditions, the scope of the alkynyl Prins annulation was explored (Scheme 8). Isobutyraldehyde outperforms all other aldehydes examined, giving access to Prins adducts **23a–c,f–i**. Both enynes and arenynes perform with comparable efficiency under these conditions. Using enyne **21a** with a 4.1:1 *E*/*Z* isomer ratio results in the formation of tosyl-piperidine **23a** in a 4:1 *E*/*Z* mixture, suggesting that alkene isomerization during annulation is minimal. The reaction of an enyne with a shorter tether is problematic, giving low yields of tosyl pyrrolidine **23c** and complex mixtures. It is not possible to prepare the azepine Prins adduct **23d** under these conditions—only a complex mixture is observed.

An alpha-methyl substituent slows reaction rates dramatically and, after heating to 40 °C for 3 days, only 28% of **23g** was obtained as a 7:1 mixture of diastereomers. Notably, the stereochemical relationship of the methyl and isopropyl substituents was assigned as *anti* by NOESY (see Supporting Information).^23^ This outcome was quite surprising because a wealth of literature reports document the formation of oxa-Prins cyclization products with *syn* disposition of substituents.^18,24^ Aldehydes with a tethered arene nucleophile react to afford products **23i**–**k**, and we note that reactions need to be kept colder for better yields. Adducts **23j** and **23k** afford *E*/*Z* mixtures, whereas **23i** is isolated as a single *E* isomer, albeit in lower yield.

To rule out thermodynamic equilibration as the origin of the *E*/*Z* mixtures, the major isomer (*E* olefin) of **23k** was isolated by column chromatography and resubjected to the reaction conditions. After 16 h, no change was observed by ^1^H NMR, indicating that the alkynyl aza-Prins reaction is irreversible and therefore kinetically controlled.

### Cross-Coupling of Aza-Prins Coupling Products

**Scheme 9:**
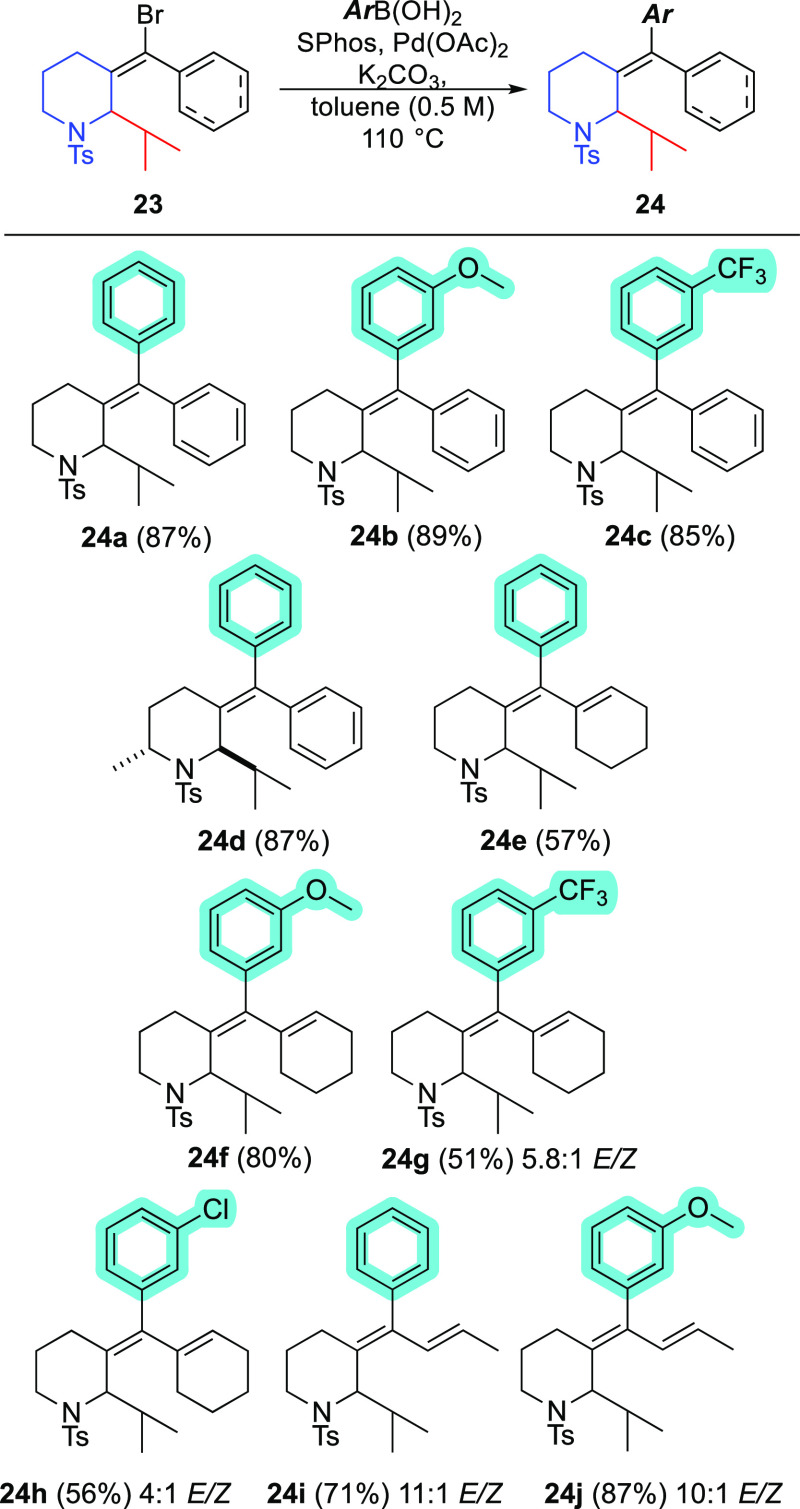
Suzuki Arylation of the Vinyl Halide

Prins adducts **23** can be further functionalized via Suzuki cross-couplings to give access to more diverse libraries of compounds (Scheme 9). After screening a few different sets of conditions, we found that SPhos, in combination with palladium acetate, potassium carbonate, and the corresponding aryl boronic acid, can afford products **24** after heating to 110 °C. Electron-neutral, rich, and deficient aryl boronic acids work well under these Suzuki conditions to afford products **24a**–**c** from arenyne-derived Prins adduct **23f**. Enyne-derived Prins adducts **23b** and **23a** also react cleanly to afford Suzuki products **24d**–**g** and **24h,i**, respectively.

The *E* geometry was retained in most cases, except cases with electron-deficient boronic acids (**24g** and **24h**). Cross-couplings of ethyl boronic acid, as well as pyridine and pyrimidine boronic acids, did not afford the corresponding cross-coupled products.

## Summary

The three variants of the alkynyl aza-Prins reaction have been developed as versatile methods for the synthesis of N-heterocycles containing a vinyl halide functional handle. We describe the construction of three different heterocyclic systems: quinazolinones from coupling primary amines (with pendent alkyne) with *o*-formyl carbamates, isoindolones from hemiaminals derived from phthalimide, and *N*-tosyl-piperidines from coupling of sulfonamides and aliphatic aldehydes. All cyclizations produced adducts with a vinyl halide functional handle, and we show that these can participate in Suzuki cross-coupling reactions.

While all variants of the alkynyl aza-Prins annulation generate piperidine ring systems smoothly, the success of five- and seven-membered ring formation varies widely. Low yields are consistently observed in the formation of pyrrolidine derivatives **16g** (Scheme 3), **18k** (Scheme 5), and **23c** (Scheme 8). For the longer tether, while no cyclization is observed at all in two cases (**16i**; Scheme 3 and **23d**; Scheme 8), the target azepine is formed in very good yield with the phthalimide-derived *N*-acyliminium electrophile (**18j**–**20j**, Scheme 5). While yields are unaffected by tether length, the *E*/*Z* selectivity in these cyclizations is significantly diminished, presumably because the typically favorable *anti*-addition pathway is compromised by the geometry of the larger ring. Notably, the corresponding cyclizations in the alkynyl oxa-Prins series are reasonably efficient: both the five- and seven-membered ring targets are formed in good yields.^17a^

A number of different combinations of promoter/halide sources have proved effective for carrying out alkynyl halo-aza-Prins cyclizations. For primary amine annulations, sodium halides combined with acetic acid are optimal, whereas the dehydrative cyclizations of phthalimide-derived hemiaminals are accomplished using either halotrimethylsilanes (TMSX) or bismuth trihalide reagents (BiX_3_). TMSX performs better for arenyne hemiaminals, while BiX_3_ salts work better for enyne hemiaminals. For annulations of sulfonamide reactants, TMSX is best. The combination of protic acid (TfOH or Tf_2_NH) and tetrabutylammonium salts, which are the conditions of choice in our alkynyl halo-oxa-Prins cyclizations,^17^ do not give good results in the halo-aza-Prins context. Regarding halide ion addition, iodide works best for the carbamate-derived iminium (Scheme 3), whereas chlorine, bromide, and iodide all perform well in the phthalimide-derived version of the reaction. Iodide was not tested in the sulfonamide annulations. In alkynyl oxa-Prins chemistry,^17^ bromide and iodide are both competent, whereas chloride is not as efficient.

The *E* isomer dominates in every aza-Prins cyclization scenario we examined, consistent with previous reports on alkynyl Prins cyclizations.^24a,25^ In the *o*-formyl carbamate annulations (Scheme 3), *E*/*Z* selectivity is moderate, whereas many of the cyclizations of phthalimide-derived iminium ions (Scheme 5) and sulfonyl iminium ions (Scheme 8) generate *E* isomer only (Scheme 5). This *E* isomer could result from either a concerted reaction pathway (*anti*-addition across the alkyne) or a stepwise one involving a vinyl cation intermediate. It was determined by experiment that the alkynyl halo-Prins cyclization is not reversible, meaning that *E*/*Z* mixtures are generated through a kinetic process. Therefore, a vinyl cation intermediate is implicated in cyclizations where the *Z* isomer is observed.

## Conclusions

These findings contribute to the expanding toolkit of synthetic chemists and hold significant promise for the synthesis of diverse nitrogen-containing compounds with potential applications in medicinal chemistry and drug discovery.

## Experimental Section

### General Remarks

All reactions were carried out under an argon atmosphere in flame-dried glassware with magnetic stirring. Syringe needles used to dispense solvent were not flame-dried. Reagents were used as obtained from commercial suppliers without further purification. Tetrahydrofuran (THF), diethyl ether (Et_2_O), methylene chloride (DCM), 1,2-dichloroethane (DCE), and toluene (PhMe) were purchased from Fisher and dispensed using the Glass Contour solvent purification system. 1,1,1,3,3,3-hexafluoropropan-2-ol (HFIP) was purchased from Oakwood Chemicals and used without further drying (bottle stored in a desiccator after opening). Celite 545 was purchased from EMD. ACS-grade hexanes, toluene, ethyl acetate, and DCM were used for column chromatography. Thin-layer chromatography (TLC) was performed on precoated silica gel 60 F254 glass-supported plates from EMD, and visualization was performed with a UV lamp followed by staining with *p*-anisaldehyde solution followed by heating. Column chromatography was carried out on EM Science silica gel (60 Å pore size, 230–400 mesh). Preparatory TLC (prep-TLC) was carried out using Analtech Uniplate F254 Prep-20 × 20 cm TLC plates. Deuterated chloroform was purchased from Cambridge Isotope Laboratories. For reactions that required heating, an oil bath was employed.

^1^H NMR spectra were recorded at room temperature on a 400 MHz Bruker AVANCE spectrometer or a 500 MHz Bruker AVANCE spectrometer. Chemical shifts are given in parts per million (ppm) referenced to solvent residual proton resonance (δ = 7.26 for CHCl_3_). NMR data are reported as chemical shift, multiplicity (s = singlet, d = doublet, t = triplet, q = quartet, m = multiplet, dd = doublet of doublets, dq = doublet of quartets, and br = broad), coupling constants (*J*) given in Hz, and integration. In those cases where two stereoisomers are present, and the ratio is greater than 3:1, only chemical shifts from the major stereoisomer are listed. For these cases, a characteristic peak from both major and minor stereoisomers is given, with proton integrations, from which the ratio of stereoisomers can be extrapolated.

The stereochemical assignments for the vinyl halide moiety (*E* vs *Z*) were made according to X-ray crystallographic data when possible, and otherwise by analogy to the overwhelming literature trend, wherein the major isomer has *E* geometry and is often the only isomer observed in alkynyl halo-Prins reactions.^1,24a,25^

^13^C NMR spectra were recorded at room temperature unless otherwise stated on a 125 or 101 MHz Bruker AVANCE spectrometer with proton decoupling. Chemical shifts are given in parts per million (ppm) from referenced to solvent carbon resonance (δ = 77.0 for CHCl_3_). In cases where two stereoisomers are present in greater than a 2:1 ratio, only chemical shifts from the major stereoisomer are listed. For these cases, a characteristic peak from both major and minor stereoisomers is given, with proton integrations, from which the ratio of stereoisomers can be extrapolated. In cases where two stereoisomers are present in less than a 2:1 ratio, all peaks are listed. For spectra where the solvent residue is present, yields were obtained after placing the sample under vacuum and bringing it to a constant weight. High-resolution mass spectra (HRMS) were measured at the University of Rochester Mass Spectrometry Resource Lab. Measurements were performed using a Thermo QExactive Plus hybrid quadrupole-Orbitrap mass spectrometer, and the scans were performed using the Orbitrap. X-ray crystallography data were collected by Dr. William W. Brennessel at the X-ray Crystallographic Facility of the University of Rochester, Rochester, NY 14627 (USA). The instrumentation used was a Rigaku XtaLab Synergy-S Dualflex diffractometer with a HyPix-6000HE HPC area detector at 100 K.

### General Procedures

The benzaldehydes **13** were prepared from the corresponding 2-aminophenyl benzoic acids: (1) reduction of the corresponding carboxylic acid to the benzyl alcohol was done with conditions from Zhao et al.,^26^ (2) aniline protection to carbamate was done using conditions from Mei et al.,^27^ and (3) reoxidation of the benzyl alcohol was done with conditions from Chong et al.^28^

Amines **15** were synthesized using the procedures below:

Mitsunobu with phthalimide and 4-pentyn-1-ol:^29^

To a flask purged with argon and equipped with a stir bar, 4-pentyn-1-ol (1.0 g, 1 equiv), phthalimide (1.3 equiv), PPh_3_ (1.3 equiv), and dry THF (125 mL) were added. The flask was brought to 0 °C, and DIAD (1.3 equiv) was added dropwise. The reaction mixture was let to be stirred for 3.5 h at room temperature until completion and monitored by TLC (usually using 20% EtOAc/hexanes as the eluent). The reaction mixture was diluted with water (125 mL) and extracted with hexanes (3 × 125 mL). The combined organic was washed with brine (125 mL) and concentrated via rotary evaporation. The crude was purified by column chromatography, eluting with 0–20% EtOAc/hexanes. Product spectra match literature precedent.

The alkynyl phthalimide above was cross-coupled to the desired vinyl group using a Sonogashira cross-coupling.^17,18^ Then, deprotection was performed using previously reported conditions^28^ to afford the amines **15**, which required no further purification.

#### General Procedure for the Synthesis of Quinazolinone **16a**

A mixture of amine **15a** (185 mg, 1.5 mmol), aldehyde **13a** (179 mg, 1.0 mmol), NaI (1.5 g, 10 mmol), and AcOH (901 mg, 858 μL, 15 mmol) in acetonitrile (5 mL, 0.2 M with respect to **13a**), under air, was allowed to stir at 80 °C for 24 h. After this time, the reaction mixture was diluted with Et_2_O (50 mL), washed with saturated sodium bicarbonate (3 × 20 mL), extracted with Et_2_O (3 × 20 mL), dried over MgSO_4_, filtered, and concentrated under vacuum. The crude material was purified by column chromatography using 10–25% ethyl acetate in hexanes as the mobile phase to give product **16a** as a golden, yellow solid (308 mg, 81%); as a 4.9:1 mixture of *E*/*Z* isomers. The compound was recrystallized by dissolving in hot methanol and allowing it to slowly cool to room temperature. ^1^H NMR (500 MHz, CDCl_3_): δ 8.21 (s, 1H), 7.23 (t, *J* = 7.6 Hz, 1H), 6.96 (t, *J* = 7.6 Hz, 1H), 6.87 (d, *J* = 7.7 Hz, 1H), 6.83 (d, *J* = 7.9 Hz, 1H), 6.17 (dd, *J* = 14.0, 7.0 Hz, 1H), 5.80–5.72 (m, 2H), 4.41 (dd, *J* = 13.5, 8.8 Hz, 1H), 2.92 (dd, *J* = 13.5, 8.1 Hz, 1H), 2.80–2.73 (m, 1H), 2.46 (dt, *J* = 13.5, 9.7 Hz, 1H), 2.02–1.95 (m, 1H), 1.83 (d, *J* = 6.8 Hz, 3H), 1.81–1.76 (m, 1H); ^13^C{^1^H} NMR (126 MHz, CDCl_3_): δ 154.8, 139.4, 138.5, 137.7, 129.0, 127.9, 125.3, 122.3, 118.7, 114.0, 102.7, 77.3, 77.0, 76.7, 58.9, 38.6, 34.6, 29.7, 22.5, 17.8; HRMS for C_16_H_17_IN_2_O (M + H) calcd, 381.0459; found, 381.0450.

#### **16b**

Purified by prep-TLC, using 20% ethyl acetate in hexanes as the mobile phase to give **16b** as a yellow solid (18 mg, 20%). Observed as a 3.5:2.2:1.3:1 mixture of *E*/*Z* isomers; ^1^H NMR (500 MHz, CDCl_3_): δ 7.65 (d, *J* = 8.1 Hz, 1H), 7.13 (td, *J* = 7.6, 1.7 Hz, 1H), 6.95 (dd, *J* = 12.7, 7.5 Hz, 1H), 6.92–6.86 (m, 1H), 6.67 (td, *J* = 7.6, 3.6 Hz, 1H), 5.14–4.89 (m, 3H), 4.67–4.55 (m, 1H), 2.87–2.81 (m, 1H), 2.66–2.55 (m, 1H), 2.49–2.36 (m, 1H), 1.97 (d, *J* = 1.3 Hz, 2H), 1.91 (s, 1H), 1.88–1.74 (m, 3H), 1.74–1.67 (m, 1H), 1.28–1.24 (m, 1H), 1.01 (d, *J* = 6.5 Hz, 1H), 0.93 (d, *J* = 6.5 Hz, 1H), 0.89 (d, *J* = 6.2 Hz, 1H); ^13^C{^1^H} NMR (126 MHz, CDCl_3_): δ 198.7, 198.0, 197.7, 197.3, 170.2, 153.2, 153.0, 135.9, 128.5, 128.4, 127.54, 127.47, 127.4, 127.2, 122.5, 121.42, 121.35, 121.3, 117.9, 117.8, 117.7, 113.6, 113.5, 113.4, 113.3, 106.1, 105.6, 105.3, 104.9, 96.2, 95.9, 95.6, 95.0, 69.3, 68.4, 68.0, 67.2, 60.8, 60.71, 60.65, 44.8, 44.7, 30.25, 30.20, 29.98, 29.95, 29.7, 25.7, 25.6, 25.4, 21.3, 21.2, 20.1, 19.8, 19.5, 19.1.; HRMS for C_18_H_20_N_2_O_3_ (M + H) calcd, 313.1547; found, 313.1546.

#### **16c**

Purified by prep-TLC, using 20% ethyl acetate in hexanes as the mobile phase. Isolated as a pale-yellow amorphous solid, as a 3.1:1 mixture of *E*/*Z* isomers (25 mg, 71%); ^1^H NMR (400 MHz, CDCl_3_): δ 7.29 (d, *J* = 8.3 Hz, 1H), 7.13 (s, 1H), 6.91 (td, *J* = 8.0, 1.9 Hz, 1H), 6.79 (d, *J* = 7.7 Hz, 1H), 6.18 (dq, *J* = 13.7, 6.8 Hz, 1H), 5.76–5.67 (m, 2H), 4.39 (dt, *J* = 13.6, 8.6 Hz, 1H), 2.93 (dd, *J* = 13.6, 8.2 Hz, 1H), 2.77 (ddd, *J* = 13.4, 11.3, 7.0 Hz, 1H), 2.48–2.37 (m, 1H), 2.04–1.93 (m, 1H), 1.86–1.80 (m, 4H); ^13^C{^1^H} NMR (101 MHz, CDCl_3_): δ 153.4, 139.0, 138.6, 134.5, 129.0, 127.6, 123.8, 122.5, 120.4, 118.4, 103.1, 58.8, 38.6, 34.4, 22.3, 17.9; HRMS (ESI) *m*/*z*: [M + H]^+^ calcd for C_16_H_16_ClIN_2_O: 415.0069; found, 415.0063.

#### **16d**

Purified by prep-TLC, using 20% ethyl acetate in hexanes as the mobile phase. Compound isolated as a yellow amorphous solid, as a 4.5:1 mixture of *E*/*Z* isomers (27 mg, 30%); ^1^H NMR (400 MHz, CDCl_3_): δ 8.24 (s, 1H), 6.77 (s, 2H), 6.64 (s, 1H), 6.28 (dt, *J* = 13.1, 6.7 Hz, 1H), 6.12 (d, *J* = 14.4 Hz, 1H), 5.59 (s, 1H), 4.42 (dd, *J* = 13.5, 8.4 Hz, 1H), 3.02 (dd, *J* = 13.3, 8.2 Hz, 1H), 2.88–2.69 (m, 1H), 2.38–2.22 (m, 5H), 2.08–1.91 (m, 1H), 1.82 (d, *J* = 6.7 Hz, 3H); ^13^C{^1^H} NMR (101 MHz, CDCl_3_): δ 155.0, 139.2, 137.8, 134.7, 134.0, 125.08, 125.05, 122.9, 121.6, 115.8, 114.5, 58.8, 38.5, 28.1, 22.4, 21.1, 18.1; HRMS for C_17_H_19_BrN_2_O (M + H) calcd, 347.0754; found, 347.0747.

#### **16e**

Purified by column chromatography, using 10–20% ethyl acetate in hexanes as the mobile phase. Compound was isolated as a yellow amorphous solid, as a 7.5:1 mixture of *E*/*Z* isomers (70 mg, 54%); ^1^H NMR (400 MHz, CDCl_3_): δ 8.20 (s, 1H), 6.76 (d, *J* = 1.8 Hz, 2H), 6.65 (s, 1H), 6.16 (dq, *J* = 13.7, 6.7 Hz, 1H), 5.76 (dd, *J* = 14.1, 1.7 Hz, 1H), 5.70 (s, 1H), 4.41 (dd, *J* = 13.5, 8.7 Hz, 1H), 2.98–2.86 (m, 1H), 2.75 (ddd, *J* = 13.3, 11.1, 7.0 Hz, 1H), 2.44 (dt, *J* = 13.6, 9.6 Hz, 1H), 2.31 (s, 3H), 1.98 (tt, *J* = 11.4, 4.9 Hz, 2H), 1.83 (dd, *J* = 6.8, 1.5 Hz, 3H); ^13^C{^1^H} NMR (101 MHz, CDCl_3_): δ 155.0, 139.7, 139.2, 138.4, 137.6, 127.8, 125.1, 123.0, 115.7, 114.6, 102.5, 58.8, 38.5, 34.5, 29.7, 22.5, 21.1, 17.8; HRMS for C_17_H_19_IN_2_O (M + H) calcd, 395.0615; found, 395.0610.

#### **16f**

Purified by prep-TLC, using 20% ethyl acetate in hexanes as the mobile phase. Compound was isolated as a yellow amorphous solid, as a 5:3:1 mixture of *E*/*Z* isomers (14 mg, 21%); ^1^H NMR (400 MHz, CDCl_3_): δ 7.46 (s, 1H), 6.78 (dd, *J* = 8.5, 2.6 Hz, 1H), 6.71 (d, *J* = 8.6 Hz, 1H), 6.47 (d, *J* = 2.7 Hz, 1H), 6.17 (dd, *J* = 14.0, 6.9 Hz, 1H), 5.77 (dd, *J* = 14.3, 1.9 Hz, 1H), 5.70 (s, 1H), 4.38 (dd, *J* = 13.6, 8.6 Hz, 1H), 3.73 (s, 3H), 2.91 (dd, *J* = 13.6, 8.1 Hz, 1H), 2.74 (ddd, *J* = 13.3, 11.3, 7.0 Hz, 1H), 2.49–2.38 (m, 1H), 2.03–1.93 (m, 1H), 1.83 (d, *J* = 6.8 Hz, 3H), 1.81–1.71 (m, 1H); ^13^C{^1^H} NMR (126 MHz, CDCl_3_): δ 155.2, 154.7, 139.4, 138.6, 131.3, 127.9, 120.2, 114.5, 113.6, 111.7, 102.7, 59.0, 55.6, 38.6, 34.6, 22.6, 17.8; HRMS for C_17_H_19_IN_2_O_2_ (M + H) calcd, 411.0564; found, 411.0555.

#### **16g**

Purified by prep-TLC, using 20% ethyl acetate in hexanes as the mobile phase. Compound isolated as a yellow amorphous solid (24 mg, 13%) ^1^H NMR (500 MHz, CDCl_3_): δ 7.77 (s, 1H), 7.27–7.15 (m, 3H), 7.04 (t, *J* = 7.6 Hz, 1H), 6.68 (d, *J* = 7.8 Hz, 3H), 6.48 (t, *J* = 7.5 Hz, 1H), 6.10 (d, *J* = 7.7 Hz, 1H), 5.48 (d, *J* = 3.3 Hz, 1H), 4.45 (dd, *J* = 13.0, 6.1 Hz, 1H), 3.31 (dtd, *J* = 40.4, 11.5, 5.6 Hz, 2H), 2.82–2.74 (m, 1H); ^13^C{^1^H} NMR (126 MHz, CDCl_3_): δ 154.2, 145.7, 142.2, 135.8, 128.7, 128.5, 128.0, 127.5, 121.1, 117.1, 113.4, 100.1, 61.9, 44.8, 38.2, 29.7; HRMS for C_18_H_15_IN_2_O (M + H) calcd, 403.0302; found, 403.0295.

#### **16h**

Purified by prep-TLC, using 20% ethyl acetate in hexanes as the mobile phase. Compound isolated as a yellow amorphous solid (23 mg, 39%); ^1^H NMR (500 MHz, CDCl_3_): δ 7.76 (s, 1H), 7.29–7.22 (m, 4H), 7.18 (q, *J* = 5.5 Hz, 2H), 7.09 (d, *J* = 7.6 Hz, 1H), 6.99 (t, *J* = 7.5 Hz, 1H), 6.73 (d, *J* = 7.8 Hz, 1H), 5.24 (s, 1H), 4.35 (dd, *J* = 13.5, 8.1 Hz, 1H), 2.94 (dt, *J* = 13.7, 7.1 Hz, 2H), 2.56 (dt, *J* = 13.4, 9.4 Hz, 1H), 2.11–1.87 (m, 3H); ^13^C{^1^H} NMR (126 MHz, CDCl_3_): δ 154.9, 142.7, 140.8, 137.8, 128.7, 128.5, 128.2, 128.0, 124.9, 122.1, 119.7, 114.0, 99.3, 57.5, 38.4, 33.5, 22.9; HRMS for C_19_H_17_IN_2_O (M + H) calcd, 417.0459; found, 417.0449.

#### **16i**

A mixture of amine **15b** (460 g, 2.82 mmol), isobutyraldehyde (515 μL, 5.64 mmol), NaI (4.24 g, 28.2 mmol), and AcOH (2.42 mL, 42.3 mmol) in acetonitrile (14 mL, 0.2 M with respect to **15b**), under air, was allowed to stir at 80 °C for 24 h. After this time, the reaction mixture was concentrated under vacuum, diluted with DCM, filtered to remove NaI salts, washed with DCM, and then concentrated once more to give a crude product **16i** as a yellow solid (1.35 g, quant.). The filtrate crude was recrystallized from warm methanol, and one crystal suitable for X-ray was obtained, the rest of the material decomposed overnight upon standing in the freezer, we could not obtain better spectra. ^1^H NMR (500 MHz, DMSO): δ 9.26 (s, 8H), 5.90 (s, 1H), 5.73 (s, 1H), 3.50 (t, *J* = 11.8 Hz, 1H), 2.89–2.70 (m, 4H), 2.56–2.43 (m, 2H), 2.33 (t, *J* = 7.1 Hz, 2H), 2.25–2.18 (m, 2H), 1.96 (d, *J* = 6.7 Hz, 8H), 1.83 (s, 15H), 1.67 (t, *J* = 7.4 Hz, 2H), 1.56 (p, *J* = 6.3 Hz, 3H), 1.47 (dp, *J* = 16.5, 5.3 Hz, 9H), 0.90 (d, *J* = 6.4 Hz, 3H), 0.62 (d, *J* = 6.6 Hz, 3H).; ^13^C{^1^H} NMR (126 MHz, CDCl_3_): δ 172.4, 139.2, 133.3, 126.6, 120.3, 85.9, 83.0, 61.0, 40.0, 39.9, 39.7, 39.5, 39.4, 39.2, 39.0, 38.9, 38.0, 34.3, 29.1, 27.9, 27.4, 26.4, 26.3, 25.3, 25.0, 24.5, 21.9, 21.8, 21.4, 21.1, 19.8, 19.7, 18.8, 15.9.; HRMS for C_15_H_24_IN (M + H) calcd, 346.1026; found, 346.1019.

Sulfonamides **21** were synthesized according to our previously reported procedure.^16^

#### General Procedure for the Synthesis of Tosyl-Piperidine **23a**

A solution of **22a** (288 mg, 365 μL, 4.0 mmol) in toluene (8 mL, 0.25 M with respect to **22a**) was cooled to −20 °C. TMSBr (1.23 g, 1.1 mL, 8.0 mmol) was added, and the mixture was allowed to stir for 5 min before adding **21a** (555 mg, 2 mmol) as a solid, in one portion. The reaction mixture was allowed to stir at that temperature until full consumption of the sulfonamide was observed by TLC (using DCM as the mobile phase and *p*-anisaldehyde to stain the plates). After this time, the reaction mixture was diluted with Et_2_O (50 mL), washed with saturated sodium bicarbonate (50 mL), extracted with Et_2_O (3 × 20 mL), washed with brine (50 mL), dried over MgSO_4_, filtered, and concentrated under vacuum. The crude material was purified with basified silica (see note 1), using 0–5% ethyl acetate in hexanes with 2% Et_3_N as the mobile phase. (Note 1: Prep-TLC plates were basified prior to use by allowing them to sit in 2% Et_3_N in hexanes for at least an hour.) Compound **23a** was obtained as a white solid, as a 4:1 mixture of *E*/*Z* isomers (629 mg, 75%). The compound was recrystallized by dissolving in hot methanol and allowing it to slowly cool to room temperature. ^1^H NMR (500 MHz, CDCl_3_): δ 7.58 (d, *J* = 8.2 Hz, 2H), 7.19 (d, *J* = 8.1 Hz, 2H), 6.50 (d, *J* = 14.4 Hz, 1H), 6.29–6.21 (m, 1H), 4.75 (d, *J* = 10.7 Hz, 1H), 3.71 (dd, *J* = 14.9, 4.6 Hz, 1H), 3.19 (td, *J* = 14.9, 3.2 Hz, 1H), 2.84 (d, *J* = 13.7 Hz, 1H), 2.39 (s, 3H), 2.22–2.14 (m, 1H), 2.09 (td, *J* = 13.7, 4.2 Hz, 2H), 1.95 (d, *J* = 6.8 Hz, 3H), 1.79–1.74 (m, 1H), 1.11 (d, *J* = 6.6 Hz, 3H), 0.95–0.91 (m, 1H), 0.87 (dd, *J* = 10.5, 5.5 Hz, 1H), 0.82 (d, *J* = 6.7 Hz, 3H); ^13^C{^1^H} NMR (126 MHz, CDCl_3_): δ 143.0, 138.0, 134.2, 133.6, 129.5, 129.3, 127.2, 127.1, 125.4, 121.4, 61.8, 40.6, 29.5, 28.2, 24.2, 21.5, 19.7, 18.9, 18.2; HRMS for C_19_H_26_BrNO_2_S (M + H) calcd, 412.0941; found, 412.0944.

#### **23b**

Purified by column chromatography with basified silica (see note 1), using 0–5% ethyl acetate in hexanes with 2% Et_3_N as the mobile phase. Isolated as an amorphous solid (39 mg, 44%). ^1^H NMR (500 MHz, CDCl_3_): δ 7.77–7.61 (m, 2H), 7.30–7.23 (m, 2H), 5.96–5.83 (m, 1H), 4.68 (d, *J* = 10.7 Hz, 1H), 3.49–3.38 (m, 1H), 3.07 (ddd, *J* = 14.4, 12.3, 3.8 Hz, 1H), 2.97 (d, *J* = 14.3 Hz, 1H), 2.41 (s, 5H), 2.21–2.04 (m, 5H), 1.77–1.60 (m, 5H), 0.95 (d, *J* = 6.6 Hz, 3H), 0.83 (d, *J* = 6.7 Hz, 3H); ^13^C{^1^H} NMR (126 MHz, CDCl_3_): δ 143.0, 137.9, 136.1, 132.9, 129.7, 129.4, 127.5, 125.2, 61.7, 40.2, 28.6, 27.8, 27.3, 25.4, 25.1, 22.4, 21.7, 21.5, 19.94, 19.89; HRMS for C_22_H_30_NO_2_S (M + H) calcd, 452.1254; found, 452.1255.

#### **23c**

Purified by column chromatography with basified silica (see note 1), using 0–5% ethyl acetate in hexanes with 2% Et_3_N as the mobile phase. Isolated as a viscous yellow wax (57 mg, 13%). ^1^H NMR (500 MHz, CDCl_3_): δ 7.69 (d, *J* = 8.0 Hz, 2H), 7.30 (d, *J* = 8.0 Hz, 2H), 5.55 (d, *J* = 4.2 Hz, 1H), 4.48 (dd, *J* = 4.5, 1.7 Hz, 1H), 3.65 (ddd, *J* = 13.5, 9.8, 4.2 Hz, 1H), 3.45 (dt, *J* = 12.7, 8.3 Hz, 1H), 2.43 (s, 3H), 2.28–2.12 (m, 2H), 2.10–2.04 (m, 2H), 1.96–1.81 (m, 2H), 1.62 (ddq, *J* = 31.3, 11.6, 5.9 Hz, 5H), 1.02 (d, *J* = 6.8 Hz, 3H), 0.87 (d, *J* = 6.9 Hz, 3H); ^13^C{^1^H} NMR (126 MHz, CDCl_3_): δ 143.6, 138.6, 137.5, 135.5, 129.6, 128.6, 127.3, 121.0, 67.8, 47.2, 34.7, 33.8, 27.5, 25.3, 22.2, 21.6, 21.5, 19.8, 18.0; HRMS for C_21_H_28_BrNO_2_S (M + H) calcd, 438.1097; found, 438.1097.

#### **23e**

Purified by column chromatography with basified silica (see note 1), using 0–5% ethyl acetate in hexanes with 2% Et_3_N as the mobile phase. Isolated as a waxy yellow solid (38 mg, 29%). ^1^H NMR (500 MHz, CDCl_3_): δ 7.70 (d, *J* = 8.3 Hz, 2H), 7.29–7.25 (m, 2H), 5.90–5.84 (m, 1H), 4.73 (d, *J* = 10.7 Hz, 1H), 3.42 (dd, *J* = 14.6, 4.7 Hz, 1H), 3.06 (ddd, *J* = 14.3, 12.7, 3.3 Hz, 1H), 2.95 (dt, *J* = 14.0, 3.6 Hz, 1H), 2.41 (s, 3H), 2.35 (q, *J* = 5.2 Hz, 1H), 2.21–2.08 (m, 3H), 2.04 (d, *J* = 17.4 Hz, 1H), 1.86–1.60 (m, 10H), 1.54–1.49 (m, 2H), 1.18–1.09 (m, 3H), 0.96–0.88 (m, 1H), 0.84–0.76 (m, 1H); ^13^C{^1^H} NMR (126 MHz, CDCl_3_): δ 143.0, 138.0, 136.1, 132.6, 129.7, 129.4, 127.5, 125.2, 60.7, 40.2, 36.5, 30.4, 29.9, 28.7, 27.8, 26.5, 26.21, 26.14, 25.3, 25.1, 22.5, 21.7, 21.5; HRMS for C_25_H_34_BrNO_2_S (M + H) calcd, 492.1566; found, 492.1570.

#### **23f**

Purified by column chromatography with basified silica (see note 1), using 0–5% ethyl acetate in hexanes with 2% Et_3_N as the mobile phase. Isolated as an amorphous white solid (175 mg, 76%). ^1^H NMR (500 MHz, CDCl_3_): δ 7.66 (d, *J* = 8.0 Hz, 2H), 7.38 (q, *J* = 7.8 Hz, 4H), 7.30 (s, 1H), 7.25 (d, *J* = 7.8 Hz, 2H), 4.30 (d, *J* = 10.7 Hz, 1H), 3.59 (d, *J* = 14.3 Hz, 1H), 3.18–2.99 (m, 2H), 2.39 (s, 3H), 2.25 (dt, *J* = 14.3, 9.3 Hz, 1H), 2.18–2.07 (m, 1H), 1.68 (s, 2H), 0.73 (t, *J* = 6.9 Hz, 6H); ^13^C{^1^H} NMR (126 MHz, CDCl_3_): δ 143.0, 139.5, 137.7, 135.7, 129.6, 129.3, 128.2, 128.0, 127.4, 119.9, 61.2, 40.0, 29.1, 28.1, 25.6, 21.4, 19.6, 19.4; HRMS for C_22_H_26_BrNO_2_S (M + H) calcd, 448.0941; found, 448.0941.

#### **23g**

TMSBr was added at 0 °C, then the reaction mixture was allowed to warm up to room temperature and then heated to 40 °C for 3 days. Purified by column chromatography with basified silica (see note 1), using 0–5% ethyl acetate in hexanes with 2% Et_3_N as the mobile phase. Product obtained as a light-yellow foam, as a 7:1 mixture of diastereomers (130 mg, 28%); ^1^H NMR (500 MHz, CDCl_3_): δ 7.72 (d, *J* = 8.0 Hz, 2H), 7.45 (d, *J* = 7.4 Hz, 2H), 7.36 (dd, *J* = 15.4, 8.0 Hz, 3H), 7.30 (d, *J* = 7.2 Hz, 2H), 7.25 (d, *J* = 6.7 Hz, 3H), 4.63 (d, *J* = 10.4 Hz, 1H), 4.58 (d, *J* = 10.7 Hz, 1H), 3.65–3.54 (m, 1H), 3.16 (d, *J* = 15.0 Hz, 1H), 2.46–2.35 (m, 5H), 2.27–2.17 (m, 1H), 1.86–1.74 (m, 2H), 1.71 (t, *J* = 13.4 Hz, 2H), 1.18 (d, *J* = 6.8 Hz, 3H), 0.91 (d, *J* = 6.5 Hz, 4H), 0.79 (d, *J* = 6.6 Hz, 3H); ^13^C{^1^H} NMR (126 MHz, CDCl_3_): δ 142.7, 141.2, 139.6, 135.6, 129.8, 129.5, 129.3, 128.3, 128.0, 127.6, 127.4, 120.7, 63.3, 49.7, 32.8, 29.7, 29.2, 21.4, 20.0, 19.7, 19.4; HRMS for C_23_H_28_BrNO_2_S (M + H) calcd, 462.1097; found, 462.1107.

#### **23h**

Purified by column chromatography with basified silica (see note 1), using 0–5% ethyl acetate in hexanes with 2% Et_3_N as the mobile phase. Isolated as a light-yellow foam (238 mg, 93%); ^1^H NMR (500 MHz, CDCl_3_): δ 7.66–7.60 (m, 2H), 7.22 (dd, *J* = 7.9, 4.7 Hz, 3H), 7.00–6.96 (m, 1H), 6.92 (dt, *J* = 7.6, 1.3 Hz, 1H), 6.82 (ddd, *J* = 8.3, 2.6, 1.0 Hz, 1H), 4.31 (d, *J* = 10.7 Hz, 1H), 3.80 (s, 3H), 3.53 (dt, *J* = 13.5, 3.3 Hz, 1H), 3.11–3.04 (m, 1H), 3.01 (ddd, *J* = 14.4, 7.9, 4.0 Hz, 1H), 2.36 (s, 3H), 2.21 (ddd, *J* = 14.4, 10.8, 8.1 Hz, 1H), 2.12–2.04 (m, 1H), 1.69–1.62 (m, 2H), 0.72 (d, *J* = 6.7 Hz, 3H), 0.69 (d, *J* = 6.6 Hz, 3H); ^13^C{^1^H} NMR (126 MHz, CDCl_3_): δ 159.0, 143.1, 140.7, 137.6, 135.7, 129.4, 129.0, 127.5, 122.1, 119.7, 114.7, 114.5, 61.2, 55.2, 40.0, 29.1, 28.1, 25.6, 21.4, 19.6, 19.4.; HRMS for C_23_H_28_BrNO_3_S (M + H) calcd, 478.1046; found, 478.1056.

#### **23i**

Purified by column chromatography with basified silica (see note 1), using 0–5% ethyl acetate in hexanes with 2% Et_3_N as the mobile phase. Isolated as a foamy solid (55 mg, 20%). ^1^H NMR (500 MHz, CDCl_3_): δ 7.68 (d, *J* = 8.1 Hz, 2H), 7.27 (d, *J* = 8.1 Hz, 2H), 6.71 (d, *J* = 7.8 Hz, 1H), 6.64–6.57 (m, 2H), 5.91 (s, 2H), 5.69–5.62 (m, 1H), 4.99 (dd, *J* = 9.3, 5.5 Hz, 1H), 3.68 (dt, *J* = 13.7, 3.7 Hz, 1H), 3.23–3.11 (m, 1H), 2.85 (dt, *J* = 14.8, 3.8 Hz, 1H), 2.56–2.50 (m, 1H), 2.42 (s, 3H), 2.18–2.04 (m, 6H), 1.71–158 (m, 7H), 1.42–132 (m, 1H).; ^13^C{^1^H} NMR (126 MHz, CDCl_3_): δ 147.5, 145.7, 143.2, 138.0, 136.5, 135.2, 133.2, 129.5, 129.2, 127.3, 124.3, 121.1, 108.8, 108.1, 100.8, 55.7, 40.2, 33.8, 32.1, 28.0, 27.5, 25.2, 24.7, 22.4, 21.7, 21.5.; HRMS for C_28_H_32_BrNO_4_S (M + H) calcd, 558.1308; found, 558.1315.

#### **23j**

Purified by column chromatography with basified silica (see note 1), using 0–5% ethyl acetate in hexanes with 2% Et_3_N as the mobile phase. Isolated as a colorless waxy solid, as an 8.8:1 mixture of *E*/*Z* isomers (73 mg, 73%). ^1^H NMR (500 MHz, CDCl_3_): δ 7.67 (dd, *J* = 12.0, 8.2 Hz, 3H), 7.33 (d, *J* = 8.0 Hz, 1H), 7.27 (d, *J* = 8.2 Hz, 2H), 7.19 (t, *J* = 7.8 Hz, 1H), 6.73 (d, *J* = 10.0 Hz, 3H), 5.65 (s, 1H), 5.02 (dd, *J* = 9.1, 5.7 Hz, 1H), 3.80 (s, 3H), 3.67 (d, *J* = 14.6 Hz, 1H), 3.21–3.12 (m, 1H), 2.86 (d, *J* = 14.7 Hz, 1H), 2.69 (s, 3H), 2.59 (ddd, *J* = 15.5, 11.9, 6.6 Hz, 1H), 2.50–2.38 (m, 7H), 2.20–1.98 (m, 7H), 1.77–1.53 (m, 9H), 1.31 (t, *J* = 7.2 Hz, 1H); ^13^C{^1^H} NMR (126 MHz, CDCl_3_): δ 159.6, 143.2, 143.0, 138.0, 136.4, 133.2, 129.6, 129.5, 129.4, 129.3, 129.2, 127.8, 127.3, 124.4, 120.7, 114.2, 111.3, 55.8, 55.2, 40.2, 37.9, 33.3, 32.4, 28.0, 27.5, 25.1, 24.7, 22.4, 21.7, 21.5; HRMS for C_28_H_34_BrNO_3_S (M + H) calcd, 544.1516; found, 544.1524.

#### **23k**

Purified by column chromatography with basified silica (see note 1), using 0–5% ethyl acetate in hexanes with 2% Et_3_N as the mobile phase. Isolated as a foamy solid, as a 3.7:1 *E*/*Z* mixture of *E*/*Z* isomers (77.6 mg, 82%). ^1^H NMR (500 MHz, CDCl_3_): δ 7.69 (d, *J* = 8.3 Hz, 2H), 7.31–7.26 (m, 4H), 7.19 (d, *J* = 7.3 Hz, 1H), 7.14 (d, *J* = 7.2 Hz, 2H), 5.67 (s, 2H), 5.03 (dd, *J* = 9.0, 5.8 Hz, 1H), 3.69 (d, *J* = 14.4 Hz, 1H), 3.24–3.13 (m, 1H), 2.87 (d, *J* = 14.5 Hz, 1H), 2.65–2.56 (m, 1H), 2.47 (ddd, *J* = 13.6, 9.8, 4.4 Hz, 1H), 2.42 (d, *J* = 5.7 Hz, 4H), 2.38–2.29 (m, 1H), 2.20–2.14 (m, 2H), 2.14–1.95 (m, 4H), 1.72 (tdd, *J* = 16.0, 11.0, 5.3 Hz, 2H), 1.68–1.51 (m, 7H), 1.44–1.34 (m, 1H); ^13^C{^1^H} NMR (126 MHz, CDCl_3_): δ 143.2, 141.4, 138.0, 136.5, 133.2, 129.5, 129.3, 129.2, 128.4, 128.3, 127.3, 127.3, 126.0, 124.4, 55.9, 40.2, 33.4, 32.4, 28.0, 27.5, 25.2, 24.8, 22.4, 21.7, 21.5; HRMS for C_27_H_32_BrNO_2_S (M + H) calcd, 514.1410; found, 514.1413.

#### General Procedure for Suzuki Cross-Coupling of Aza-Prins Adducts to Afford Functionalized Tosyl-Piperidine **24a**

A mixture of **23f** (174 mg, 0.39 mmol), PhB(OH)_2_ (95 mg, 0.79 mmol), SPhos (16 mg, 0.039 mmol), Pd(OAc)_2_ (4.4 mg, 0.019 mmol), and K_2_CO_3_ (161 mg, 1.2 mmol) in toluene (0.8 mL) was sparged with argon for 30 min, then heated to 110 °C for 24 h. After this time, the reaction mixture was diluted with Et_2_O (20 mL) and filtered through celite, rinsed with Et_2_O, and then concentrated under vacuum. The obtained product was purified by column chromatography, using 5% ethyl acetate in hexanes with 2% Et_3_N as the mobile phase. The product was obtained as a white foamy solid (139 mg, 87%). ^1^H NMR (500 MHz, CDCl_3_): δ 7.77–7.69 (m, 2H), 7.32 (t, *J* = 7.6 Hz, 2H), 7.27 (m, 7H), 7.22–7.18 (m, 1H), 7.12–7.04 (m, 2H), 4.49 (d, *J* = 10.6 Hz, 1H), 3.68–3.59 (m, 1H), 3.21–3.07 (m, 1H), 2.47 (d, *J* = 14.1 Hz, 1H), 2.42 (s, 3H), 2.29–2.15 (m, 2H), 1.55 (m, 2H), 0.88 (d, *J* = 6.7 Hz, 3H), 0.83 (d, *J* = 6.6 Hz, 3H); ^13^C{^1^H} NMR (126 MHz, CDCl_3_): δ 142.8, 142.6, 141.2, 138.7, 138.4, 133.2, 129.9, 129.3, 129.1, 128.1, 128.0, 127.6, 126.8, 126.6, 61.5, 40.3, 28.1, 26.4, 25.9, 21.5, 19.9, 19.8; HRMS for C_28_H_31_NO_2_S (M + H) calcd, 446.2148; found, 446.2148.

#### **24b**

Purified by column chromatography with basified silica (see note 1), using 0–5% ethyl acetate in hexanes with 2% Et_3_N as the mobile phase. Isolated as a white foam (142 mg, 89%); ^1^H NMR (500 MHz, CDCl_3_): δ 7.78–7.67 (m, 2H), 7.35–7.24 (m, 6H), 7.24–7.15 (m, 2H), 6.73 (dd, *J* = 8.1, 2.6 Hz, 1H), 6.67 (dt, *J* = 7.5, 1.2 Hz, 1H), 6.60 (dd, *J* = 2.6, 1.5 Hz, 1H), 4.47 (d, *J* = 10.6 Hz, 1H), 3.73 (s, 3H), 3.63 (dt, *J* = 14.4, 3.4 Hz, 1H), 3.20–3.06 (m, 1H), 2.47 (dd, *J* = 13.8, 3.5 Hz, 1H), 2.40 (s, 3H), 2.30–2.11 (m, 1H), 1.62–1.47 (m, 2H), 0.87 (d, *J* = 6.8 Hz, 3H), 0.83 (d, *J* = 6.6 Hz, 3H); ^13^C{^1^H} NMR (126 MHz, CDCl_3_): δ 159.3, 143.9, 142.7, 140.9, 138.3, 133.2, 129.7, 129.2, 129.1, 127.9, 127.5, 126.7, 121.4, 115.0, 111.4, 61.3, 55.0, 40.3, 28.0, 26.4, 25.9, 21.4, 19.8, 19.7; HRMS for C_29_H_33_NO_3_S (M + H) calcd, 476.2254; found, 476.2262.

#### **24c**

Purified by column chromatography with basified silica (see note 1), using 0–5% ethyl acetate in hexanes with 2% Et_3_N as the mobile phase. Isolated as a white foam (147 mg, 85%); ^1^H NMR (500 MHz, CDCl_3_): δ 7.75–7.66 (m, 2H), 7.45 (d, *J* = 7.8 Hz, 1H), 7.41–7.31 (m, 3H), 7.31–7.22 (m, 7H), 4.48 (d, *J* = 10.6 Hz, 1H), 3.65 (dt, *J* = 14.3, 3.5 Hz, 1H), 3.13 (m, 1H), 2.40 (s, 3H), 2.34 (dt, *J* = 14.1, 3.5 Hz, 1H), 2.28–2.17 (m, 2H), 1.56 (m, 2H), 0.88 (d, *J* = 6.8 Hz, 3H), 0.82 (d, *J* = 6.5 Hz, 3H); ^13^C{^1^H} NMR (126 MHz, CDCl_3_): δ 143.2, 143.0, 140.3, 138.1, 137.3, 134.7, 132.5, 129.8, 129.3, 128.7, 128.1, 127.6, 127.1, 125.5, 125.5, 123.4, 61.3, 40.2, 28.0, 26.4, 25.9, 21.4, 19.8; HRMS for C_29_H_30_F_3_NO_2_S (M + H) calcd, 514.2022; found, 514.2033.

#### **24d**

Purified by column chromatography with basified silica (see note 1), using 0–5% ethyl acetate in hexanes with 2% Et_3_N as the mobile phase. Isolated as a white foam, as an 11:1 mixture of diastereomers (67 mg, 87%); ^1^H NMR (500 MHz, CDCl_3_): δ 7.83 (d, *J* = 8.2 Hz, 2H), 7.36–7.32 (m, 5H), 7.28 (dd, *J* = 13.8, 7.9 Hz, 6H), 7.25–7.18 (m, 5H), 4.84 (d, *J* = 10.4 Hz, 1H), 4.74 (d, *J* = 10.8 Hz, 1H), 3.64 (dd, *J* = 8.8, 5.9 Hz, 1H), 2.54 (dd, *J* = 10.7, 3.9 Hz, 1H), 2.48–2.32 (m, 7H), 1.70–1.59 (m, 2H), 1.59–1.51 (m, 1H), 1.19 (d, *J* = 6.9 Hz, 3H), 0.99 (d, *J* = 6.6 Hz, 3H), 0.90 (d, *J* = 6.7 Hz, 3H); ^13^C{^1^H} NMR (126 MHz, CDCl_3_): δ 142.8, 142.5, 141.7, 141.3, 138.9, 132.9, 129.8, 129.2, 129.1, 128.2, 128.0, 127.9, 127.5, 127.4, 126.7, 126.5, 63.5, 49.8, 33.7, 28.8, 26.5, 21.4, 20.1, 19.9, 19.8; HRMS for C_29_H_33_NO_2_S (M + H) calcd, 460.2305; found, 460.2310.

#### **24e**

Purified by prep-TLC, using 20% ethyl acetate in hexanes with 2% Et_3_N as the mobile phase. Isolated as an amorphous white solid (28.6 mg, 57%). ^1^H NMR (500 MHz, CDCl_3_): δ 7.79 (d, *J* = 7.9 Hz, 2H), 7.28 (dd, *J* = 15.3, 7.9 Hz, 4H), 7.24–7.20 (m, 1H), 7.11 (d, *J* = 7.7 Hz, 2H), 5.81 (s, 1H), 4.86 (d, *J* = 10.7 Hz, 1H), 3.52–3.41 (m, 1H), 3.17–3.05 (m, 1H), 2.41 (s, 3H), 2.21 (ddd, *J* = 48.6, 31.8, 17.0 Hz, 4H), 2.10–1.97 (m, 1H), 1.89 (d, *J* = 15.7 Hz, 1H), 1.77 (d, *J* = 17.5 Hz, 1H), 1.56 (d, *J* = 4.2 Hz, 4H), 1.42 (dd, *J* = 13.0, 9.6 Hz, 2H), 1.03 (t, *J* = 7.7 Hz, 3H), 0.94 (t, *J* = 11.8 Hz, 3H); ^13^C{^1^H} NMR (126 MHz, CDCl_3_): δ 142.7, 141.2, 141.1, 138.6, 137.2, 130.8, 129.3, 128.8, 127.9, 127.6, 126.5, 126.4, 61.9, 40.4, 28.2, 27.2, 26.3, 25.3, 25.2, 22.8, 22.1, 21.5, 20.2, 20.2; HRMS for C_28_H_35_NO_2_S (M + H) calcd, 450.2462; found, 450.2468.

#### **24f**

Purified by column chromatography with basified silica (see note 1), using 0–5% ethyl acetate in hexanes with 2% Et_3_N as the mobile phase. Isolated as an amorphous solid (102 mg, 80%). ^1^H NMR (500 MHz, CDCl_3_): δ 7.77 (d, *J* = 7.9 Hz, 2H), 7.25 (d, *J* = 7.9 Hz, 2H), 7.19 (t, *J* = 7.8 Hz, 1H), 6.75 (dd, *J* = 8.2, 2.5 Hz, 1H), 6.68 (d, *J* = 7.6 Hz, 1H), 6.63 (d, *J* = 2.5 Hz, 1H), 5.81–5.76 (m, 1H), 4.82 (d, *J* = 10.6 Hz, 1H), 3.77 (s, 3H), 3.45 (dd, *J* = 13.8, 4.3 Hz, 1H), 3.10 (ddd, *J* = 15.0, 12.3, 4.0 Hz, 1H), 2.39 (s, 3H), 2.27 (dt, *J* = 14.3, 4.0 Hz, 1H), 2.23–2.11 (m, 3H), 2.00 (m, 1H), 1.92–18.87 (m, 1H), 1.78–1.75 (m, 1H), 1.59–1.51 (m, 4H), 1.41 (dt, *J* = 8.3, 4.8 Hz, 2H), 1.00 (dd, *J* = 7.1, 1.8 Hz, 3H), 0.93 (d, *J* = 6.6 Hz, 3H); ^13^C{^1^H} NMR (126 MHz, CDCl_3_): δ 159.2, 142.7, 142.6, 141.0, 138.5, 137.0, 130.9, 129.2, 128.8, 127.5, 126.4, 121.4, 114.8, 111.3, 61.8, 55.1, 46.2, 40.4, 28.2, 27.2, 26.3, 25.3, 25.2, 22.8, 22.0, 21.4, 20.2, 20.1, 11.6; HRMS for C_29_H_37_NO_3_S (M + H) calcd, 480.2567; found, 480.2566.

#### **24g**

Purified by column chromatography with basified silica (see note 1), using 0–5% ethyl acetate in hexanes with 2% Et_3_N as the mobile phase. Isolated as a light-yellow foam, as a 5.8:1 mixture of *E*/*Z* isomers (53 mg, 51%); ^1^H NMR (500 MHz, CDCl_3_): δ 7.78 (d, *J* = 8.1 Hz, 2H), 7.48 (d, *J* = 6.8 Hz, 1H), 7.42 (t, *J* = 7.7 Hz, 1H), 7.37 (d, *J* = 9.9 Hz, 1H), 7.29 (dd, *J* = 13.0, 8.4 Hz, 4H), 5.88 (s, 1H), 4.85 (d, *J* = 10.7 Hz, 1H), 3.48 (d, *J* = 12.8 Hz, 1H), 3.15–3.05 (m, 1H), 2.41 (d, *J* = 9.2 Hz, 3H), 2.38 (d, *J* = 12.2 Hz, 2H), 2.26–2.13 (m, 5H), 2.09–2.00 (m, 1H), 1.88 (d, *J* = 16.4 Hz, 1H), 1.75 (d, *J* = 16.8 Hz, 1H), 1.57 (d, *J* = 4.9 Hz, 6H), 1.46 (s, 2H), 1.02 (d, *J* = 6.5 Hz, 3H), 0.96 (d, *J* = 6.7 Hz, 3H); ^13^C{^1^H} NMR (126 MHz, CDCl_3_): δ 142.9, 141.9, 139.9, 138.3, 136.6, 132.3, 132.2, 129.3, 129.2, 128.7, 128.4, 127.6, 127.5, 127.4, 127.3, 125.4, 123.4, 61.7, 40.3, 28.2, 27.2, 26.3, 25.3, 25.2, 22.8, 22.0, 21.4, 20.2, 20.1; HRMS for C_29_H_34_F_3_NO_2_S (M + H) calcd, 518.2335; found, 518.2327.

#### **24h**

Purified by column chromatography with basified silica (see note 1), using 0–5% ethyl acetate in hexanes with 2% Et_3_N as the mobile phase. Isolated as a white foam, as a 4:1 mixture of *E*/*Z* isomers (54 mg, 56%); ^1^H NMR (500 MHz, CDCl_3_): δ 7.77 (d, *J* = 8.0 Hz, 2H), 7.28 (d, *J* = 8.0 Hz, 2H), 7.24–7.19 (m, 2H), 7.07 (s, 1H), 6.99 (d, *J* = 6.9 Hz, 1H), 5.82 (s, 1H), 4.83 (d, *J* = 10.7 Hz, 1H), 3.47 (d, *J* = 12.7 Hz, 1H), 3.15–3.04 (m, 1H), 2.42 (s, 4H), 2.33–1.99 (m, 10H), 1.87 (s, 1H), 1.76 (d, *J* = 16.6 Hz, 1H), 1.57 (d, *J* = 2.4 Hz, 5H), 1.43 (dd, *J* = 14.6, 10.4 Hz, 3H), 1.01 (d, *J* = 7.1 Hz, 3H), 0.94 (d, *J* = 6.7 Hz, 3H); ^13^C{^1^H} NMR (126 MHz, CDCl_3_): δ 143.0, 142.8, 140.0, 138.4, 136.7, 133.8, 131.9, 129.5, 129.3, 129.2, 128.7, 127.6, 127.3, 127.1, 127.1, 126.7, 61.7, 40.3, 28.1, 27.2, 26.3, 25.4, 25.2, 22.8, 22.0, 21.4, 20.2, 20.1; HRMS for C_28_H_34_ClNO_2_S (M + H) calcd, 484.2072; found, 484.2070.

#### **24i**

Purified by column chromatography with basified silica (see note 1), using 0–5% ethyl acetate in hexanes with 2% Et_3_N as the mobile phase. Isolated as a white foam, as an 11:1 mixture of *E*/*Z* isomers (312 mg, 71%); ^1^H NMR (500 MHz, CDCl_3_): δ 7.73 (t, *J* = 6.5 Hz, 2H), 7.29–7.15 (m, 7H), 6.75 (d, *J* = 15.1 Hz, 1H), 5.10–4.99 (m, 1H), 4.82 (d, *J* = 10.7 Hz, 1H), 3.84 (dd, *J* = 14.9, 4.2 Hz, 1H), 3.20 (td, *J* = 15.0, 2.9 Hz, 1H), 2.43 (s, 3H), 2.41–2.35 (m, 1H), 2.28–2.19 (m, 1H), 1.87–1.72 (m, 4H), 1.18 (d, *J* = 6.5 Hz, 3H), 1.16–1.02 (m, 2H), 0.94 (d, *J* = 6.7 Hz, 3H); ^13^C{^1^H} NMR (126 MHz, CDCl_3_): δ 142.5, 140.2, 139.0, 136.4, 130.8, 130.5, 129.5, 129.2, 129.1, 129.0, 127.9, 127.5, 127.4, 127.4, 127.3, 127.2, 126.3, 60.4, 40.9, 27.6, 25.7, 24.9, 21.3, 19.7, 19.0, 18.6; HRMS for C_25_H_31_NO_2_S (M + H) calcd, 410.2148; found, 410.2150.

#### **24j**

Purified by column chromatography with basified silica (see note 1), using 0–5% ethyl acetate in hexanes with 2% Et_3_N as the mobile phase. Isolated as a light-yellow foam, as a 10:1 mixture of *E*/*Z* isomers (192 mg, 87%); ^1^H NMR (500 MHz, CDCl_3_): δ 7.72 (d, *J* = 8.2 Hz, 2H), 7.24 (d, *J* = 8.0 Hz, 2H), 7.15 (t, *J* = 7.9 Hz, 1H), 6.74 (dd, *J* = 14.2, 8.7 Hz, 2H), 5.15–5.06 (m, 1H), 4.81 (d, *J* = 10.7 Hz, 1H), 3.74 (s, 3H), 3.20 (td, *J* = 14.9, 2.9 Hz, 1H), 2.42 (s, 3H), 2.40 (d, *J* = 7.5 Hz, 1H), 2.29–2.16 (m, 2H), 1.80 (d, *J* = 6.4 Hz, 6H), 1.26–1.19 (m, 2H), 1.18 (d, *J* = 6.5 Hz, 3H), 1.17–1.08 (m, 1H), 0.93 (d, *J* = 6.7 Hz, 4H), 0.92–0.86 (m, 1H); ^13^C{^1^H} NMR (126 MHz, CDCl_3_): δ 159.3, 142.6, 141.8, 139.0, 136.2, 130.8, 130.5, 129.7, 129.3, 129.2, 129.2, 128.9, 128.4, 127.5, 127.4, 127.4, 123.2, 121.6, 115.1, 111.5, 111.4, 60.4, 55.0, 40.9, 27.6, 25.8, 25.0, 21.4, 19.8, 19.1, 18.7; HRMS for C_26_H_33_NO_3_S (M + H) calcd, 440.2254; found, 440.2258.

## Data Availability

The data underlying this study are available in the published article and its Supporting Information.

## Supporting Information Available

Crystallographic data and NMR spectra (PDF)

## Accession Codes

CCDC 2269089–2269091 contain the supplementary crystallographic data for this paper. These data can be obtained free of charge via www.ccdc.cam.ac.uk/data_request/cif, or by emailing data_request@ccdc.cam.ac.uk, or by contacting the Cambridge Crystallographic Data Centre, 12 Union Road, Cambridge CB2 1EZ, UK; fax: +44 1223 336033.
