# Supplementary material for: Alkynyl Halo-Aza-Prins Annulative Couplings
Source: J Org Chem. 2023 Nov 16;88(23):16065–75. doi: 10.1021/acs.joc.3c01305 (PMC10696554; doi:10.1021/acs.joc.3c01305)
Supplement: Supplementary file 1 — jo3c01305_si_001.pdf [file jo3c01305_si_001.pdf]

Alkynyl *halo-aza*-Prins Annulative Couplings

Jackson J. Hernandez; Alexandra P. Lawrie; Alison J. Frontier*

Department of Chemistry, University of Rochester, 414 Hutchison Hall, 100 Trustee Road, Rochester, NY 14627-0216 (USA).

*Corresponding Author: alison.frontier@rochester.edu

Table of Contents:

X-Ray Data **S3**

NMR Data **S7**

X-Ray Data:

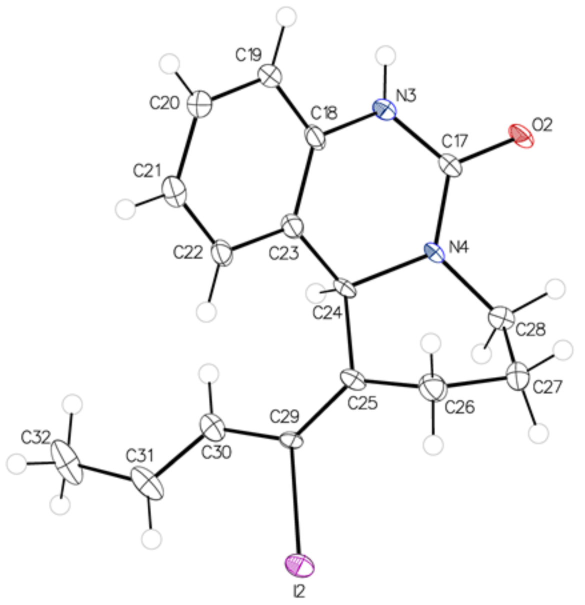

Table S1. Crystal data and structure refinement for **16a.** Ellipsoids drawn at the 50% probability level.

Identification code	**16a**	
Empirical formula	C16.25 H18 I N2 O1.25	
Formula weight	388.23	
Temperature	100.00(10) K	
Wavelength	1.54184 Å	
Crystal system	monoclinic	
Space group	$P2_1/n$	
Unit cell dimensions	a = 10.25081(12) Å	α = 90°
	b = 17.90015(16) Å	β = 92.8359(10)°
	c = 16.93092(18) Å	γ = 90°
Volume	3102.87(6) Å^3	
Z	8	
Density (calculated)	1.662 Mg/m^3	
Absorption coefficient	16.217 mm^{-1}	
F(000)	1540	
Crystal color, morphology	colourless, block	
Crystal size	0.147 x 0.135 x 0.114 mm^3	
Theta range for data collection	3.596 to 77.861°	
Index ranges	$-12 \le h \le 12$, $-12 \le k \le 22$, $-21 \le l \le 21$	

Reflections collected	29114
Independent reflections	6525 [R(int) = 0.0511]
Observed reflections	6161
Completeness to theta = 74.504°	100.0%
Absorption correction	Multi-scan
Max. and min. transmission	1.00000 and 0.36859
Refinement method	Full-matrix least-squares on F^2
Data / restraints / parameters	6525 / 0 / 383
Goodness-of-fit on F^2	1.087
Final R indices [I>2sigma(I)]	$R1$ = 0.0404, $wR2$ = 0.1085
R indices (all data)	$R1$ = 0.0424, $wR2$ = 0.1101
Largest diff. peak and hole	0.796 and -1.771 e.Å^{-3}

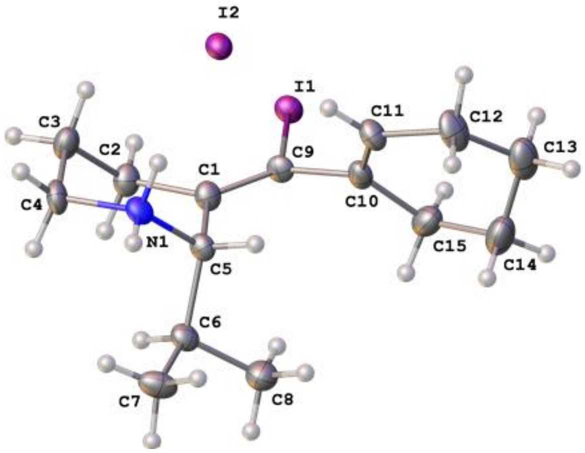

Table S2. Crystal data and structure refinement for **16i.** Ellipsoids drawn at the 50% probability level.

Identification code	**16i**	
Empirical formula	C15 H25 I2 N	
Formula weight	473.16	
Temperature	100.00(10) K	
Wavelength	1.54184 Å	
Crystal system	monoclinic	
Space group	$P2_1/c$	
Unit cell dimensions	a = 8.3088(2) Å	α = 90°
	b = 12.6175(3) Å	β = 97.868(2)°
	c = 16.7978(4) Å	γ = 90°

Volume	1744.44(7) Å^3
Z	4
Density (calculated)	1.802 Mg/m^3
Absorption coefficient	28.190 mm^{-1}
F(000)	912
Crystal color, morphology	colourless, needle
Crystal size	0.333 x 0.04 x 0.026 mm^3
Theta range for data collection	4.398 to 77.775°
Index ranges	$-10 \le h \le 10$, $-15 \le k \le 15$, $-21 \le l \le 20$
Reflections collected	27294
Independent reflections	3688 [R(int) = 0.0970]
Observed reflections	3384
Completeness to theta = 74.504°	100.0%
Absorption correction	Multi-scan
Max. and min. transmission	1.00000 and 0.53589
Refinement method	Full-matrix least-squares on F^2
Data / restraints / parameters	3688 / 0 / 165
Goodness-of-fit on F^2	1.078
Final R indices [I>2sigma(I)]	$R1$ = 0.0442, $wR2$ = 0.1265
R indices (all data)	$R1$ = 0.0464, $wR2$ = 0.1287
Largest diff. peak and hole	1.493 and -1.518 e.Å^{-3}

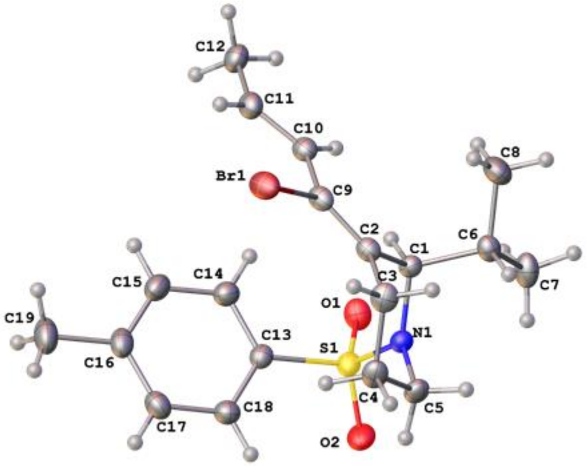

Table S3. Crystal data and structure refinement for **23a.** Ellipsoids drawn at the 50% probability level.

Identification code	**23a**	
Empirical formula	C19 H26 Br N O2 S	
Formula weight	412.38	
Temperature	100.00(10) K	
Wavelength	1.54184 Å	
Crystal system	monoclinic	
Space group	*C*2/*c*	
Unit cell dimensions	*a* = 26.6447(3) Å	α = 90°
	b = 10.77090(10) Å	β = 92.0630(10)°
	c = 13.4469(2) Å	γ = 90°
Volume	3856.59(8) $Å^3$	
Z	8	
Density (calculated)	1.420 Mg/m^3	
Absorption coefficient	3.999 mm^{-1}	
F(000)	1712	
Crystal color, morphology	colourless, block	
Crystal size	0.256 x 0.235 x 0.163 mm^3	
Theta range for data collection	3.320 to 77.596°	
Index ranges	$-33 \leq h \leq 29$, $-13 \leq k \leq 13$, $-17 \leq l \leq 14$	
Reflections collected	16944	
Independent reflections	4043 [*R*(int) = 0.0290]	
Observed reflections	3962	
Completeness to theta = 74.504°	99.9%	
Absorption correction	Multi-scan	
Max. and min. transmission	1.00000 and 0.63139	
Refinement method	Full-matrix least-squares on F^2	
Data / restraints / parameters	4043 / 0 / 221	
Goodness-of-fit on F^2	1.055	
Final *R* indices [*I*>2sigma(*I*)]	*R*1 = 0.0320, *wR*2 = 0.0846	
R indices (all data)	*R*1 = 0.0326, *wR*2 = 0.0850	
Largest diff. peak and hole	0.650 and -0.758 e.$Å^{-3}$	

NMR Data:

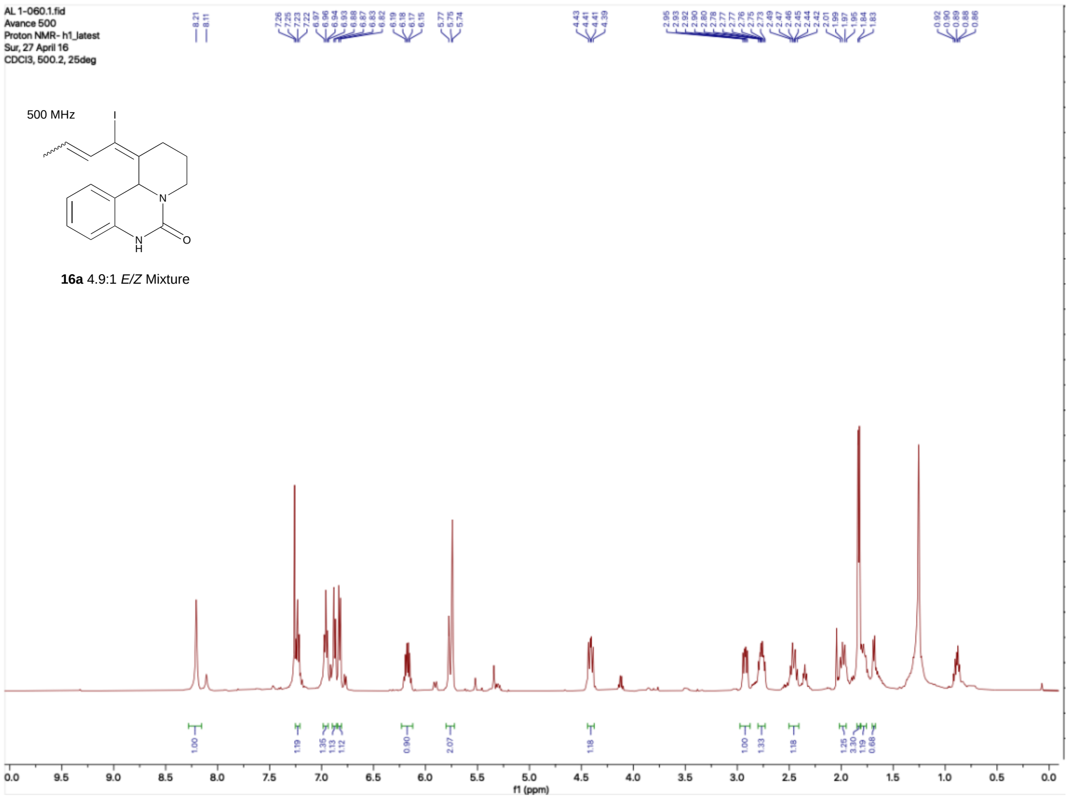
AL 1-060.1.fid
Avance 500
Proton NMR- h1_latest
Sur, 27 April 16
CDCl3, 500.2, 25deg
500 MHz
16a 4.9:1 E/Z Mixture
f1 (ppm)

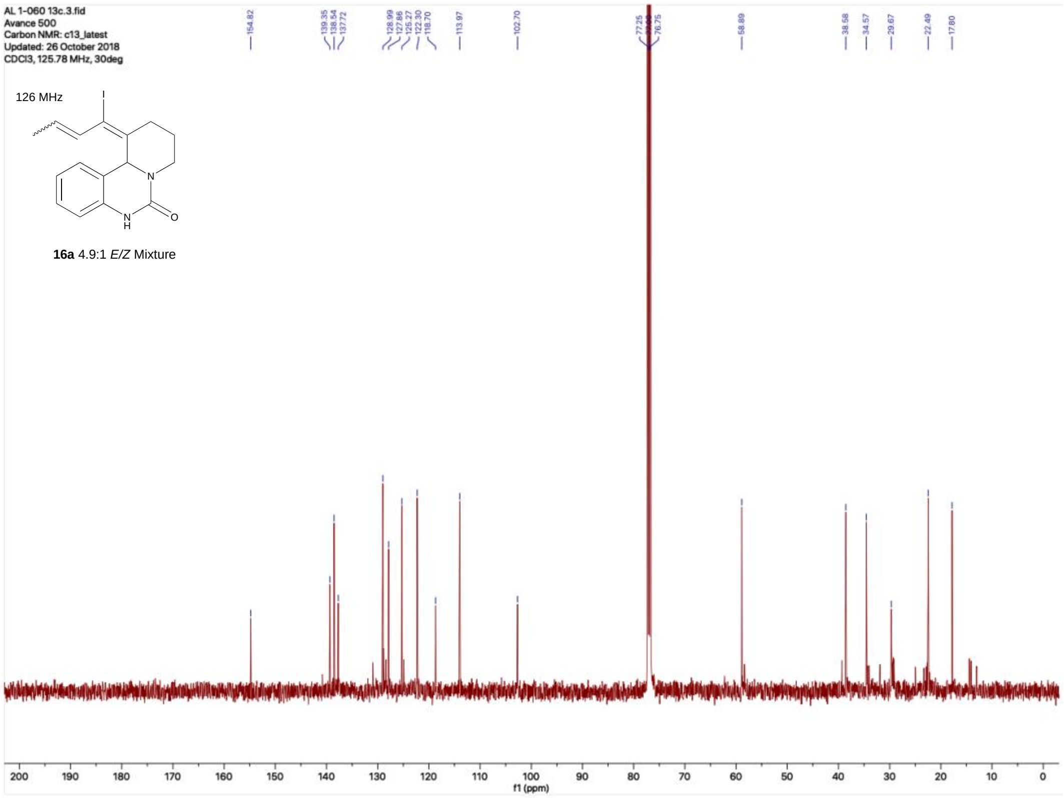
AL 1-060 13c.3.fid
Avance 500
Carbon NMR: c13_latest
Updated: 26 October 2018
CDCl3, 125.78 MHz, 30deg
126 MHz
16a 4.9:1 E/Z Mixture
f1 (ppm)

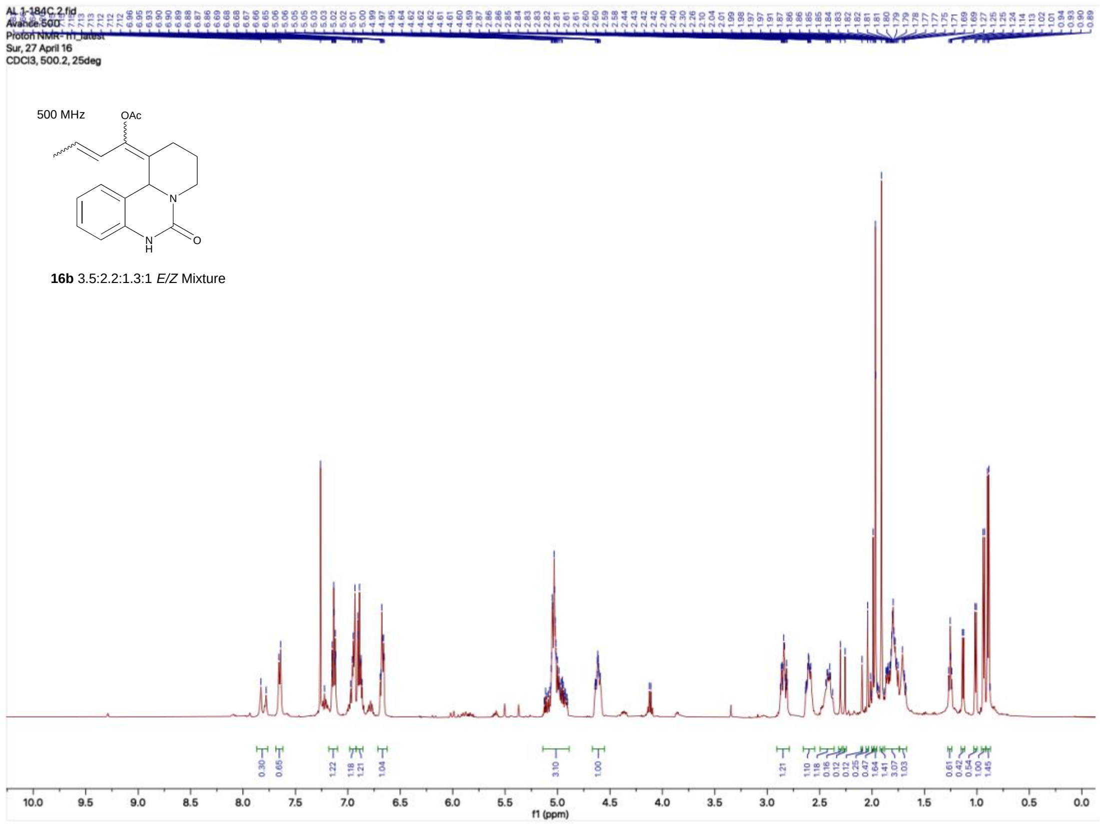
AL 1-184C.2.fid
Avance 500
Proton NMR: h1_latest
Sun, 27 April 16
CDCl3, 500.2, 25deg
500 MHz
OAc
N
H
O
16b 3.5:2.2:1.3:1 E/Z Mixture
f1 (ppm)

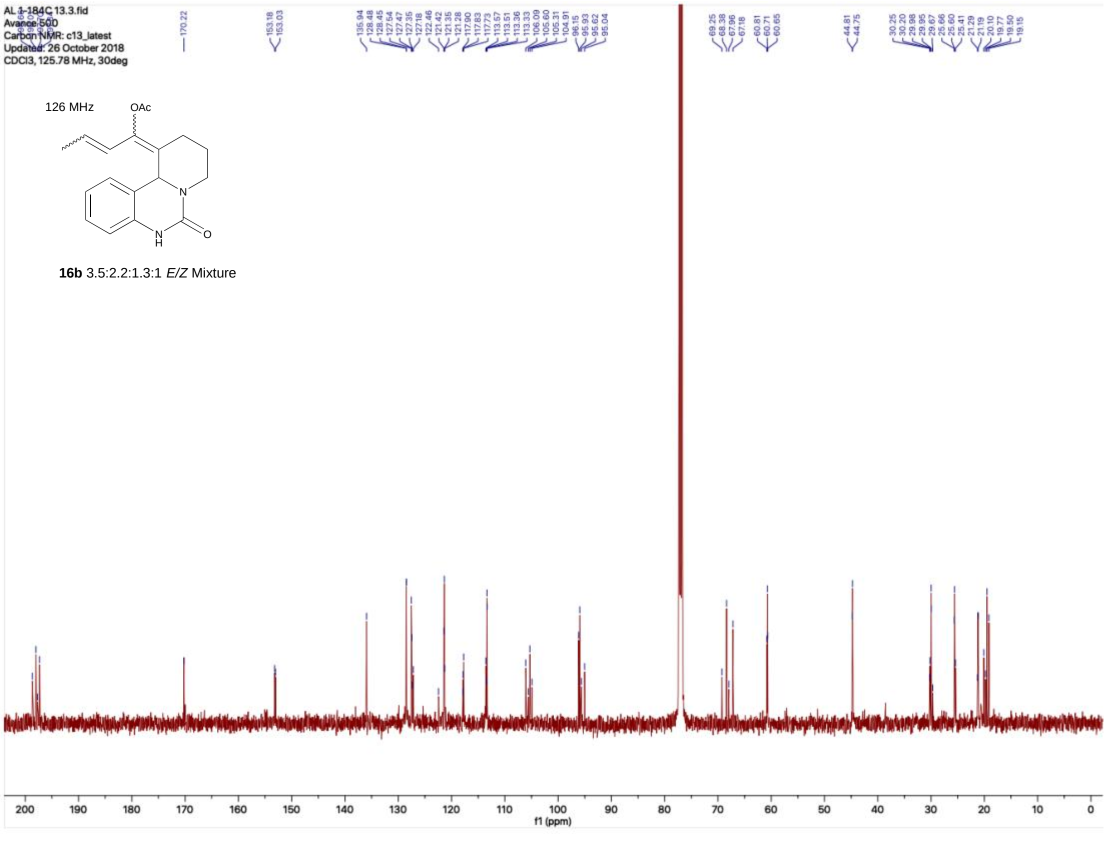
AL 1-184C.13.3.fid
Avance 500
Carbon NMR: c13_latest
Updated: 26 October 2018
CDCl3, 125.78 MHz, 30deg
126 MHz
OAc
N
H
O
16b 3.5:2.2:1.3:1 E/Z Mixture
f1 (ppm)

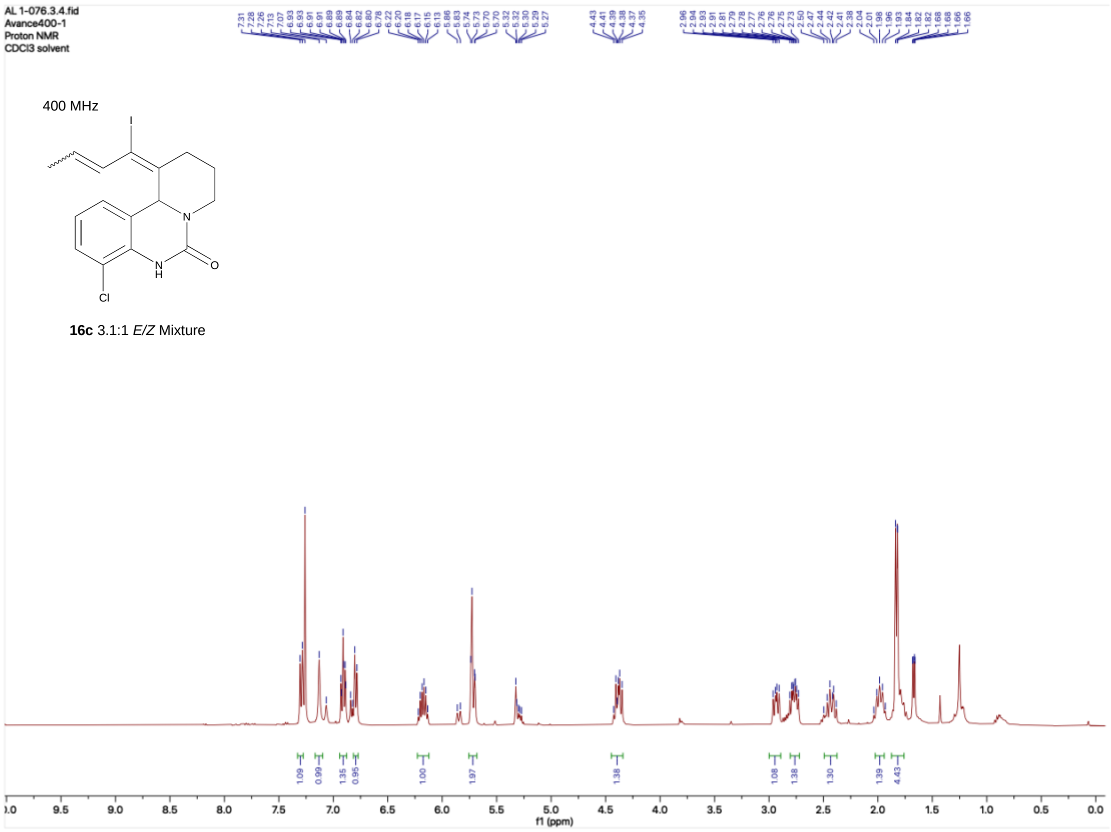
AL 1-076.3.4.fid
Avance400-1
Proton NMR
CDCl3 solvent
400 MHz
I
N
H
O
Cl
16c 3.1:1 *E/Z* Mixture
1.09
0.99
1.35
0.95
1.00
1.97
1.38
1.08
1.38
1.30
1.39
4.43
f1 (ppm)

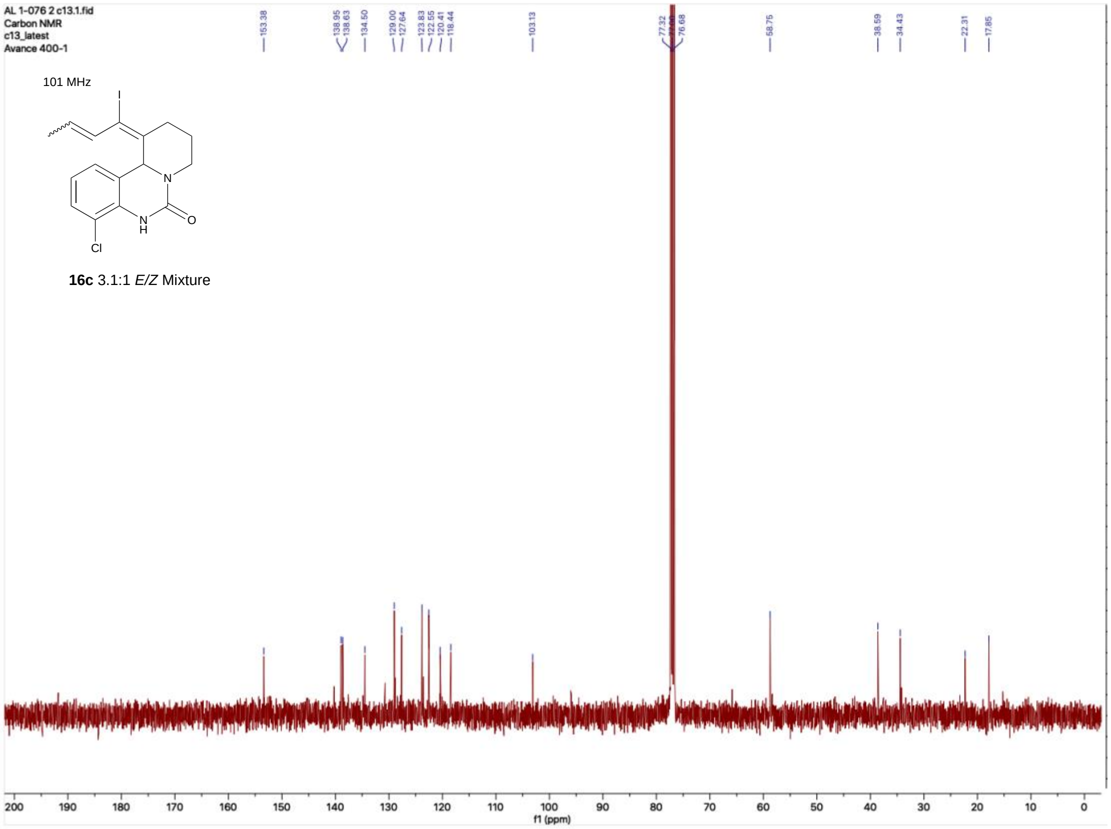
AL 1-076 2 c13.1.fid
Carbon NMR
c13_latest
Avance 400-1
101 MHz
153.38
138.95
138.63
134.50
129.00
127.64
123.83
122.55
120.41
118.44
103.13
77.32
76.68
58.75
38.59
34.43
22.31
17.85
I
N
H
O
Cl
16c 3.1:1 *E/Z* Mixture
f1 (ppm)

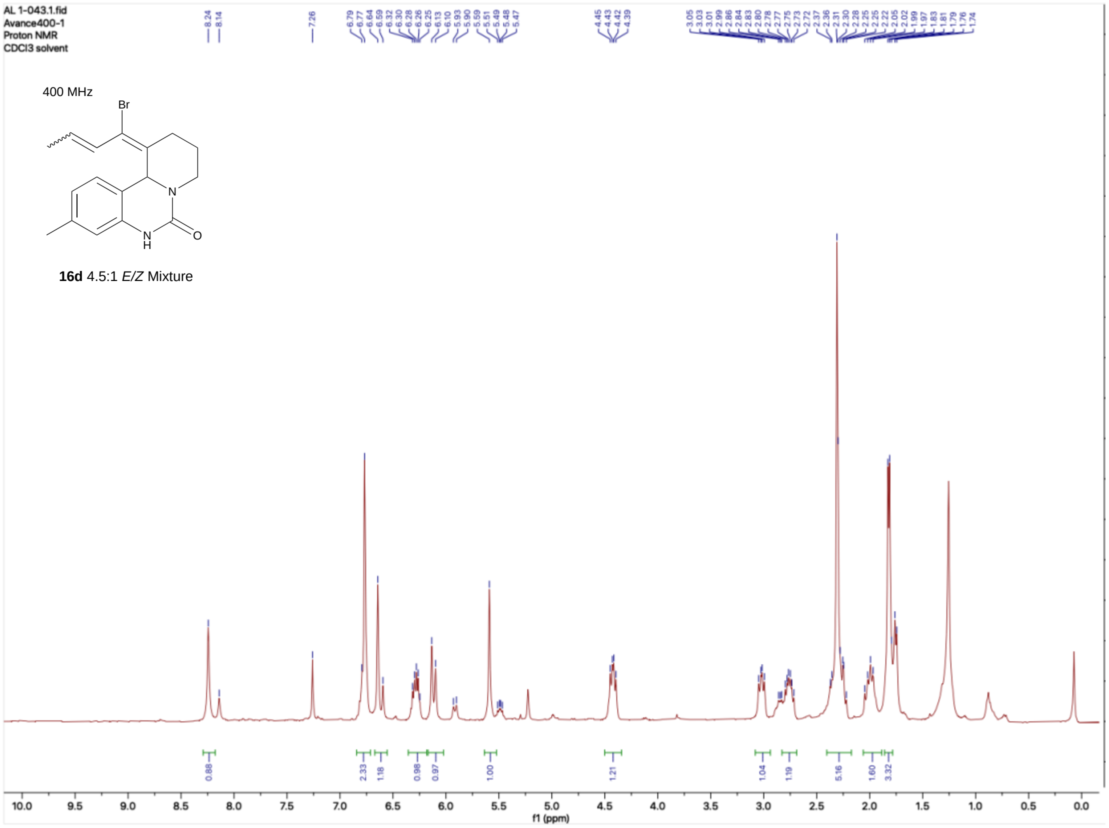
AL 1-043.1.fid
Avance400-1
Proton NMR
CDCl3 solvent
8.24 8.14
7.26
6.79 6.77 6.64 6.59 6.32 6.30 6.28 6.26 6.25 6.13 6.10 5.93 5.90 5.59 5.51 5.49 5.48 5.47
4.45 4.43 4.42 4.39
3.05 3.03 3.01 2.99 2.86 2.84 2.83 2.80 2.78 2.77 2.75 2.73 2.72 2.37 2.36 2.31 2.30 2.28 2.25 2.22 2.05 2.02 1.99 1.97 1.83 1.81 1.79 1.76 1.74
400 MHz
Br
N
H
O
16d 4.5:1 E/Z Mixture
0.88
2.33 1.18
0.98 0.97
1.00
1.21
1.04 1.19
5.16 1.60 3.32
f1 (ppm)

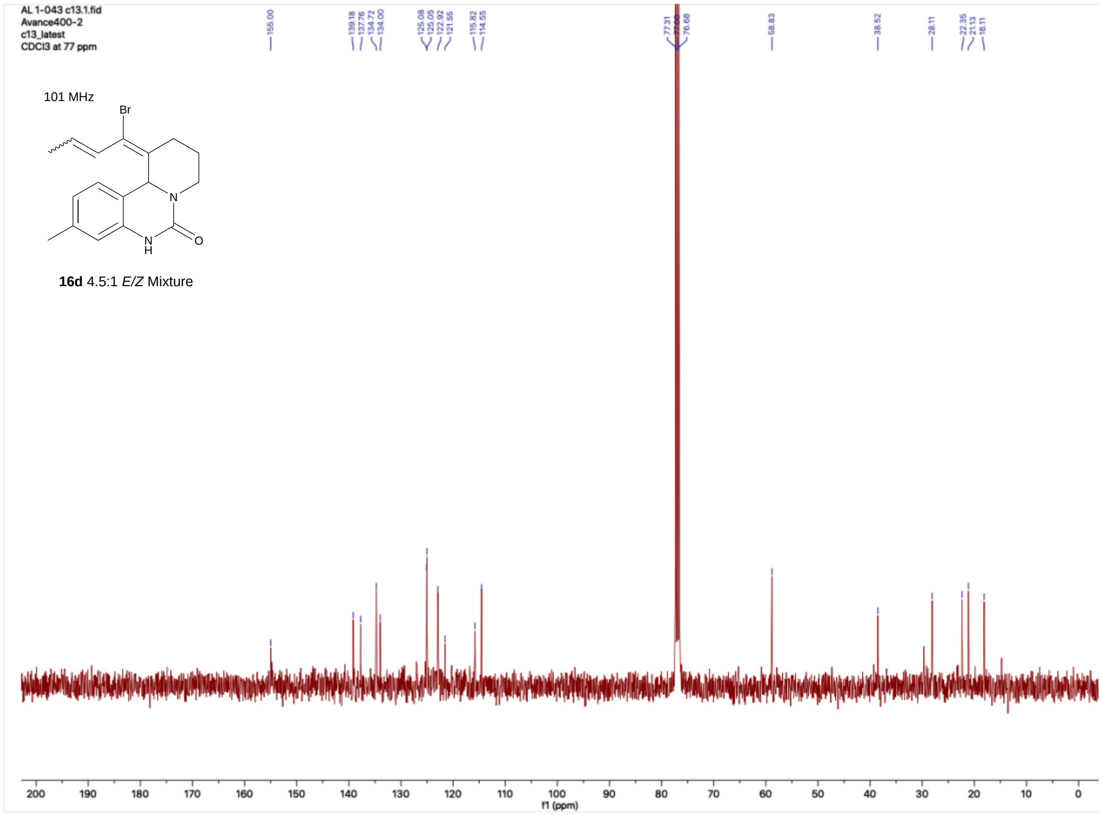
AL 1-043 c13.1.fid
Avance400-2
c13_latest
CDCl3 at 77 ppm
155.00
139.18 137.76 134.72 134.00
125.08 125.05 122.92 121.56
115.82 114.55
77.31 77.00 76.68
58.83
38.52
28.11
22.35 21.13 18.11
101 MHz
Br
N
H
O
16d 4.5:1 E/Z Mixture
f1 (ppm)

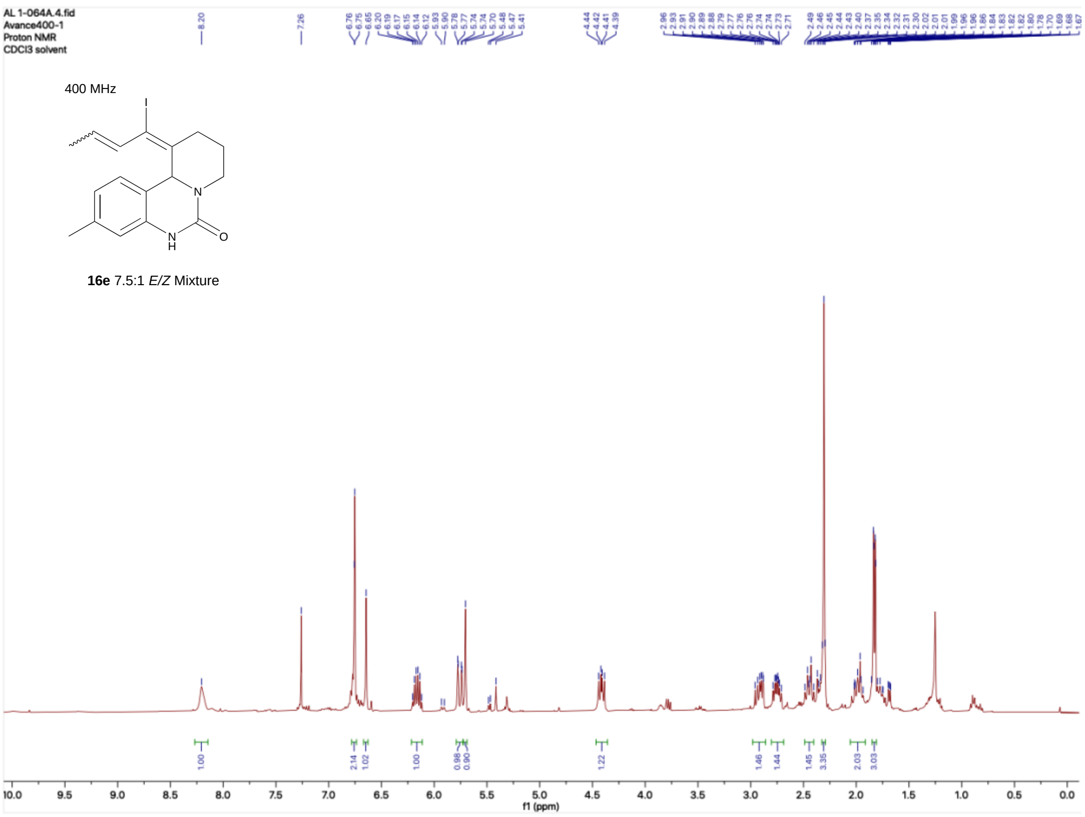
AL 1-064A.4.fid
Avance400-1
Proton NMR
CDCl3 solvent
400 MHz
I
N
N
H
O
16e 7.5:1 E/Z Mixture
f1 (ppm)

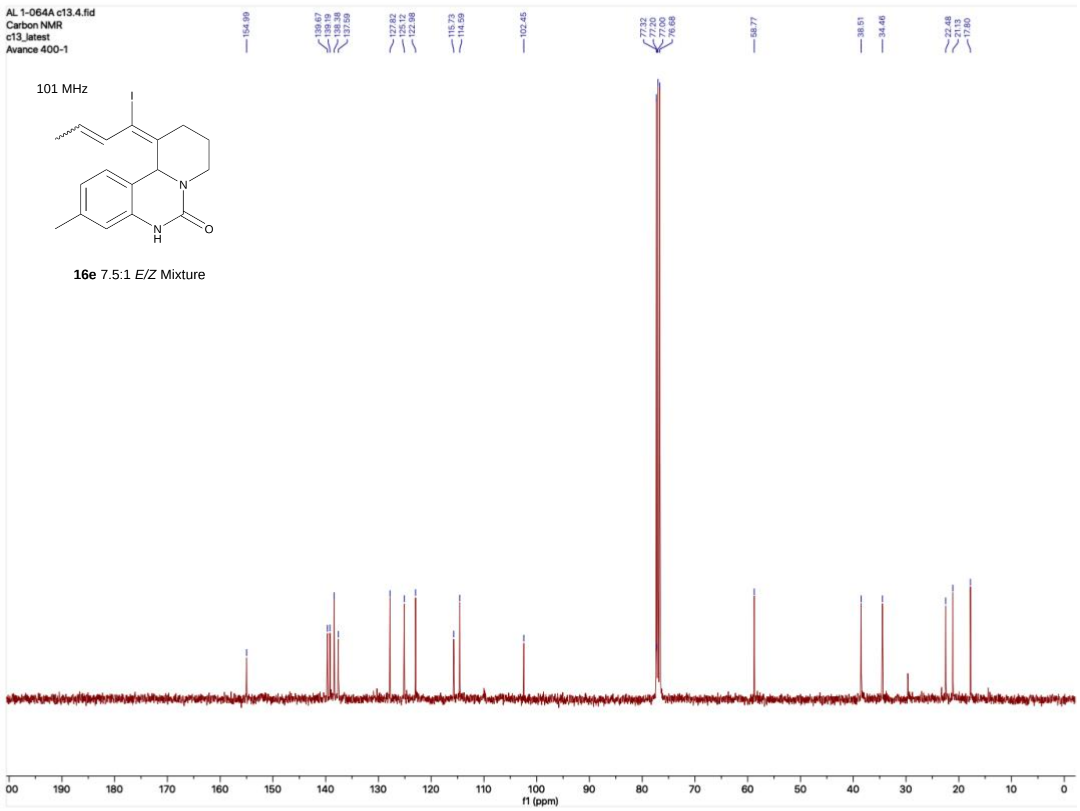
AL 1-064A c13.4.fid
Carbon NMR
c13_latest
Avance 400-1
154.99
139.67
139.19
138.38
137.59
127.82
125.12
122.98
115.73
114.59
102.45
77.32
77.20
77.00
76.68
58.77
38.51
34.46
22.48
21.13
17.80
101 MHz
I
N
N
H
O
16e 7.5:1 E/Z Mixture
f1 (ppm)

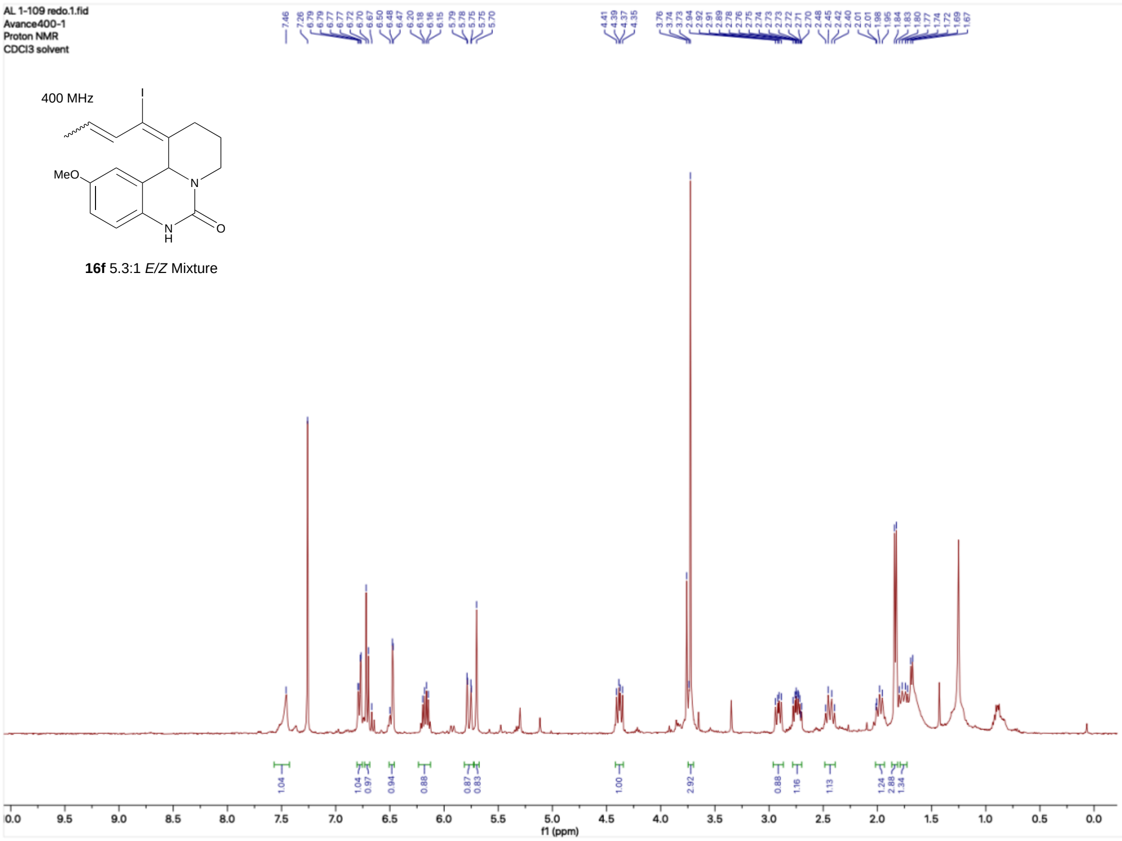

AL 1-109 redo.1.fid
Avance400-1
Proton NMR
CDCl3 solvent
400 MHz
I
MeO
N
N
H
O
16f 5.3:1 E/Z Mixture
7.46
7.26
6.79
6.79
6.77
6.77
6.72
6.70
6.67
6.50
6.48
6.47
6.20
6.18
6.16
6.15
5.79
5.78
5.75
5.75
5.70
4.41
4.39
4.37
4.35
3.76
3.74
3.73
2.94
2.92
2.91
2.89
2.78
2.76
2.75
2.74
2.73
2.73
2.72
2.71
2.70
2.48
2.45
2.42
2.40
2.01
2.01
1.98
1.95
1.84
1.83
1.80
1.77
1.74
1.72
1.69
1.67
1.04
1.04
0.97
0.94
0.88
0.87
0.83
1.00
2.92
0.88
1.16
1.13
1.24
2.88
1.34
10.0 9.5 9.0 8.5 8.0 7.5 7.0 6.5 6.0 5.5 5.0 4.5 4.0 3.5 3.0 2.5 2.0 1.5 1.0 0.5 0.0
f1 (ppm)

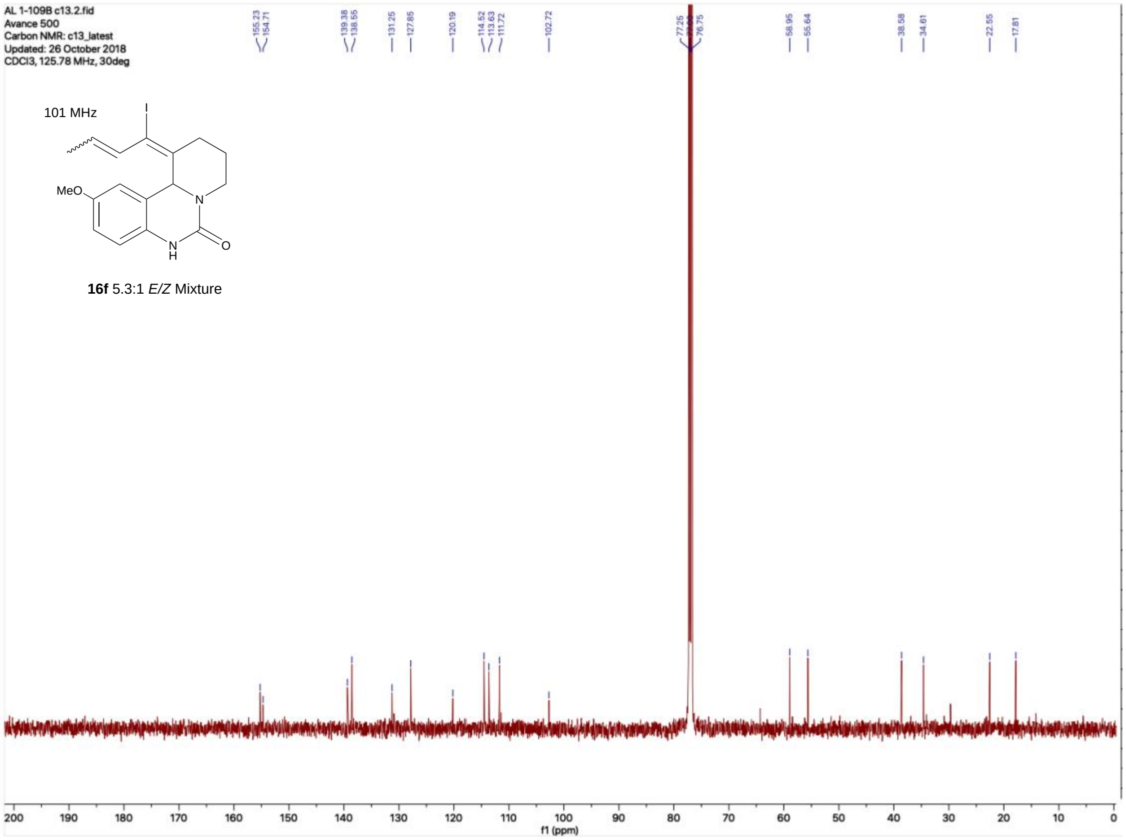

AL 1-109B c13.2.fid
Avance 500
Carbon NMR: c13_latest
Updated: 26 October 2018
CDCl3, 125.78 MHz, 30deg
101 MHz
I
MeO
N
N
H
O
16f 5.3:1 E/Z Mixture
155.23
154.71
139.38
138.55
131.25
127.85
120.19
114.52
113.63
111.72
102.72
77.25
76.75
58.95
55.64
38.58
34.61
22.55
17.81
200 190 180 170 160 150 140 130 120 110 100 90 80 70 60 50 40 30 20 10 0
f1 (ppm)

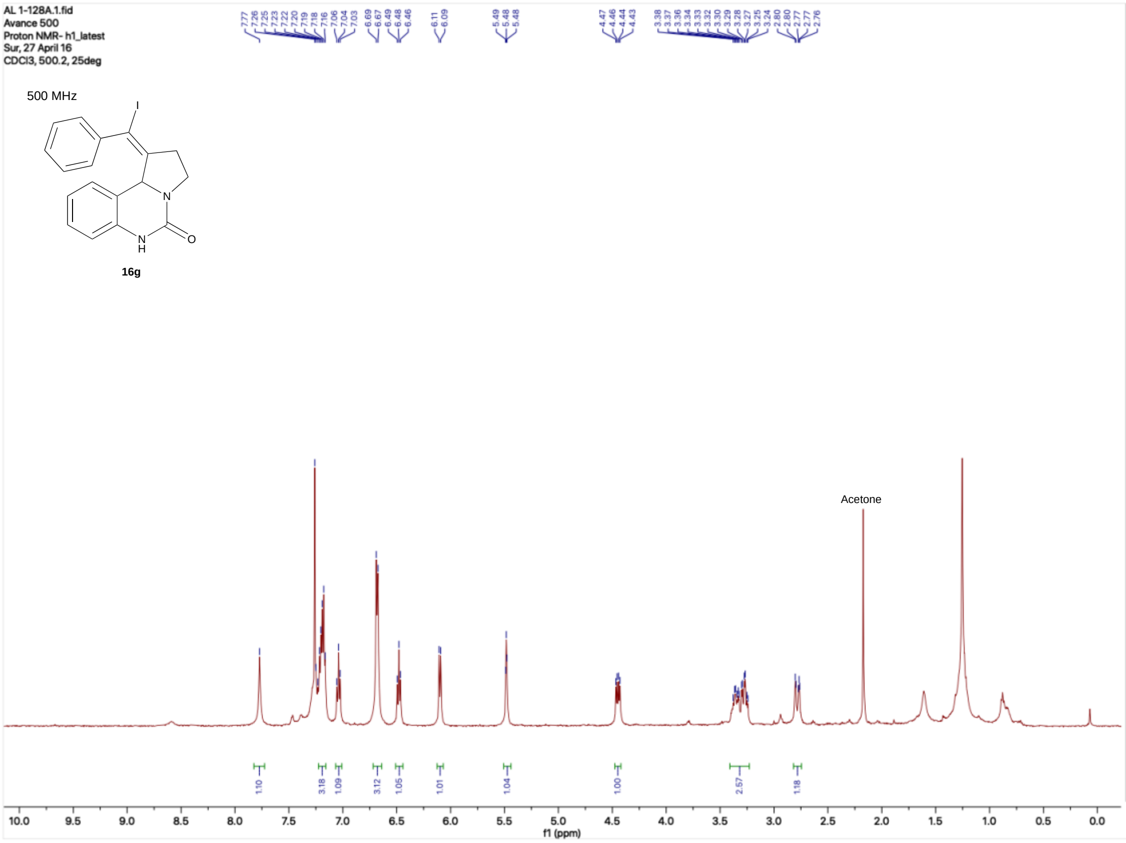
AL 1-128A.1.fid
Avance 500
Proton NMR- h1_latest
Sur, 27 April 16
CDCl3, 500.2, 25deg
500 MHz
16g
Acetone
f1 (ppm)

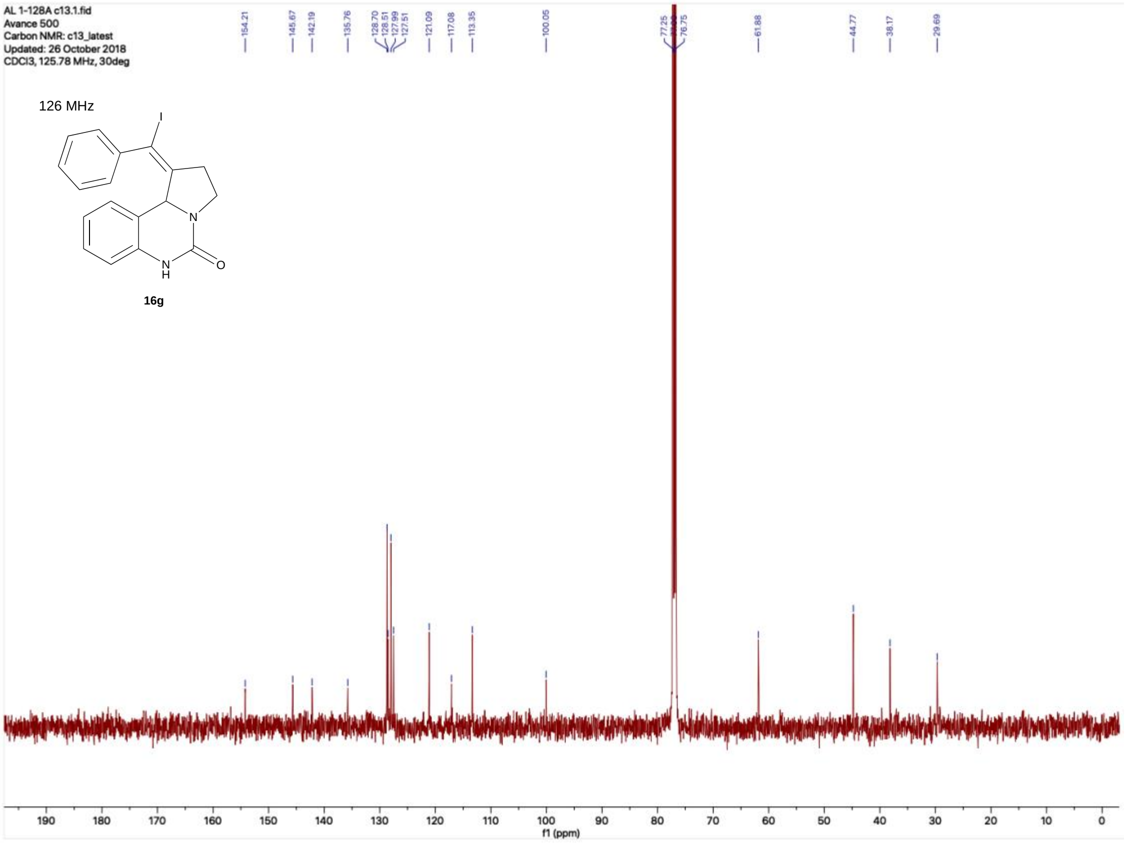
AL 1-128A c13.1.fid
Avance 500
Carbon NMR: c13_latest
Updated: 26 October 2018
CDCl3, 125.78 MHz, 30deg
126 MHz
16g
f1 (ppm)

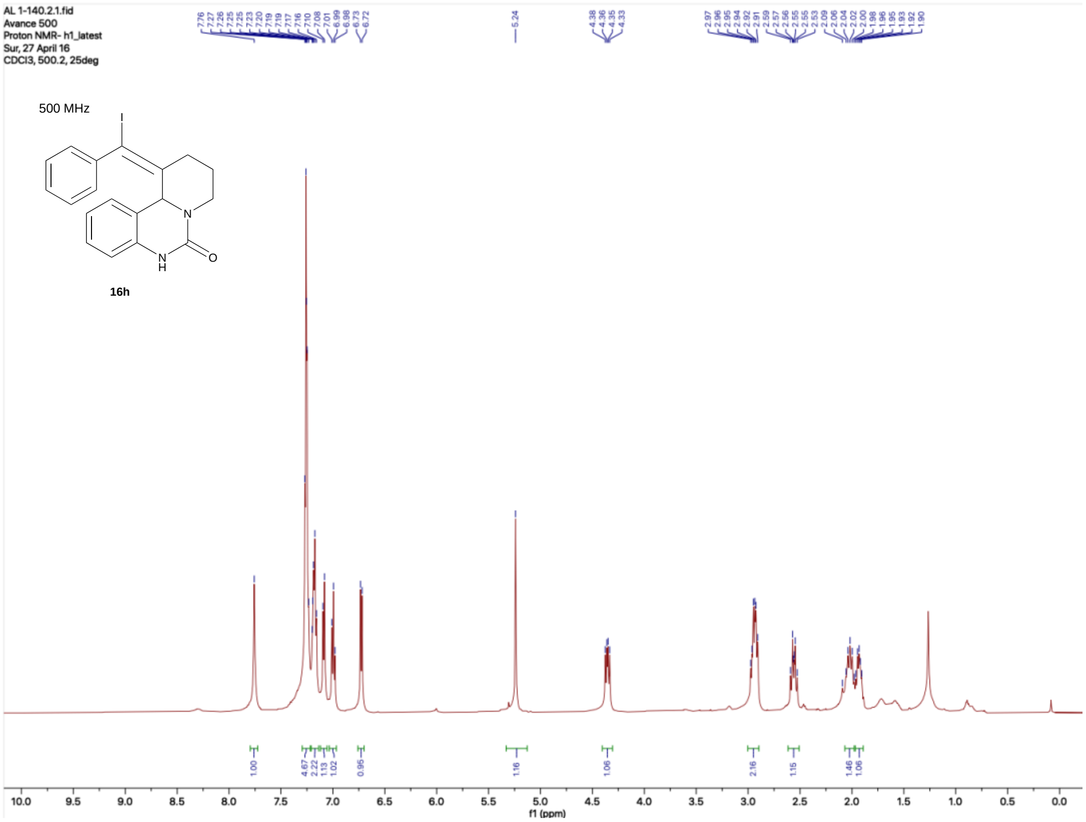
AL 1-140.2.1.fid
Avance 500
Proton NMR- h1_latest
Sur, 27 April 16
CDCl3, 500.2, 25deg
500 MHz
16h
f1 (ppm)

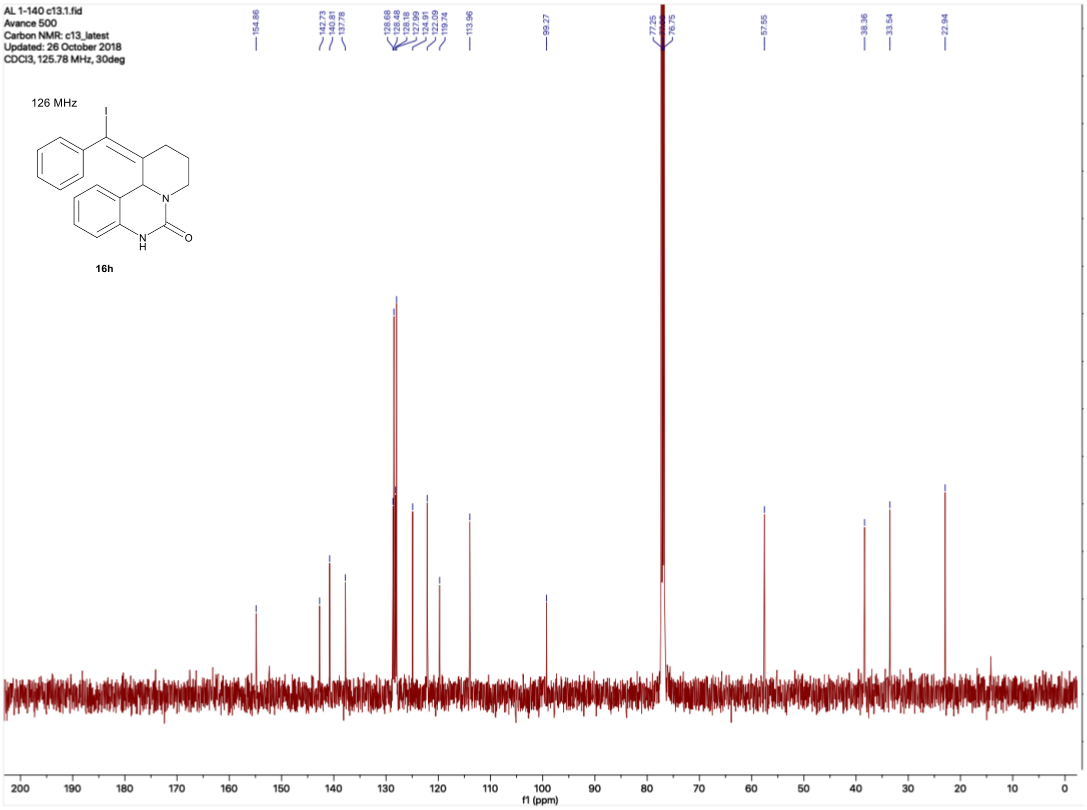
AL 1-140 c13.1.fid
Avance 500
Carbon NMR: c13_latest
Updated: 26 October 2018
CDCl3, 125.78 MHz, 30deg
126 MHz
16h
154.86
142.73
140.81
137.78
128.68
128.48
128.18
127.99
124.91
122.09
119.74
113.96
99.27
77.25
76.75
57.55
38.36
33.54
22.94
f1 (ppm)

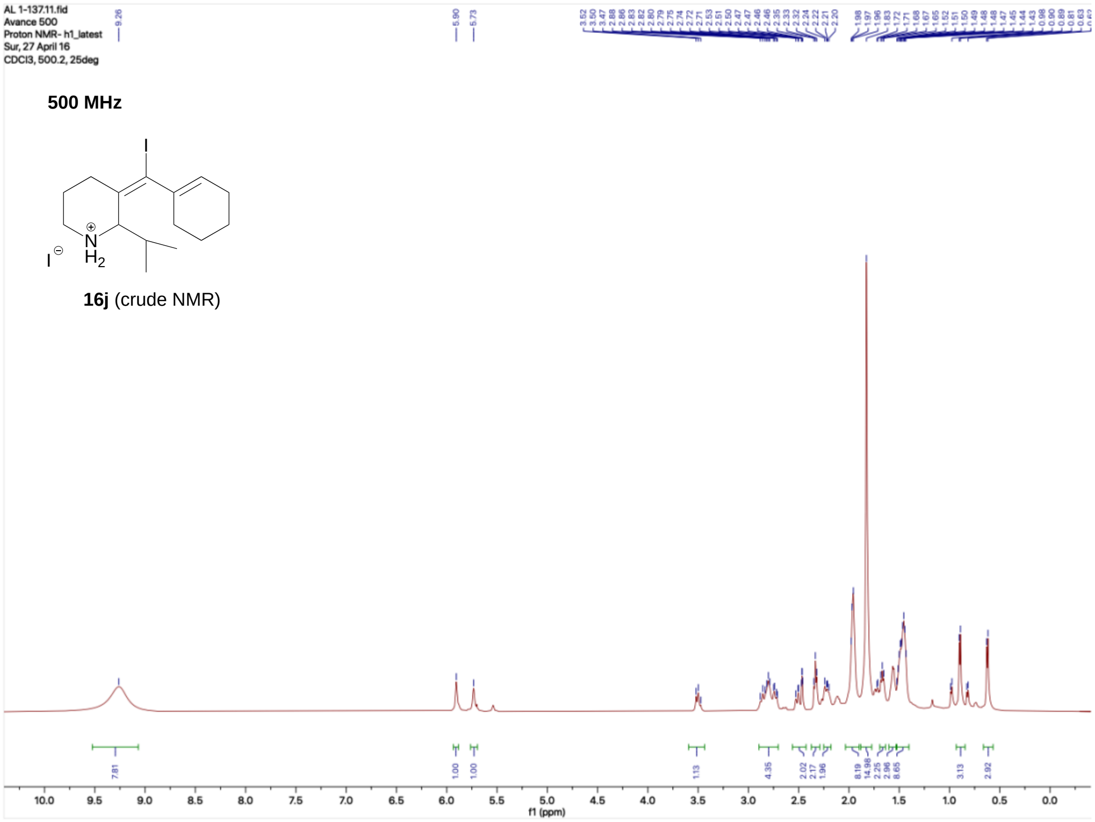
AL 1-137.11.fid
Avance 500
Proton NMR- h1_latest
Sur, 27 April 16
CDCl3, 500.2, 25deg
500 MHz
I
N
H2
I
16j (crude NMR)
f1 (ppm)

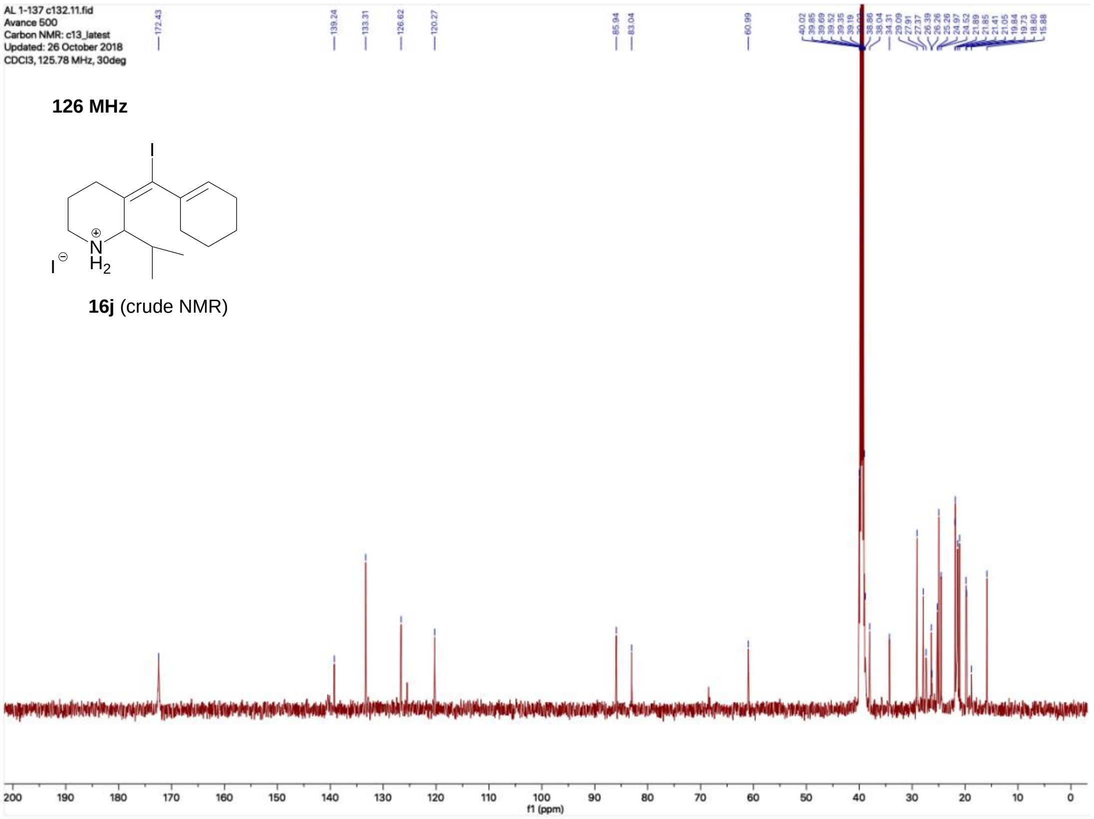
AL 1-137 c132.11.fid
Avance 500
Carbon NMR: c13_latest
Updated: 26 October 2018
CDCl3, 125.78 MHz, 30deg
126 MHz
I
N
H2
I
16j (crude NMR)
f1 (ppm)

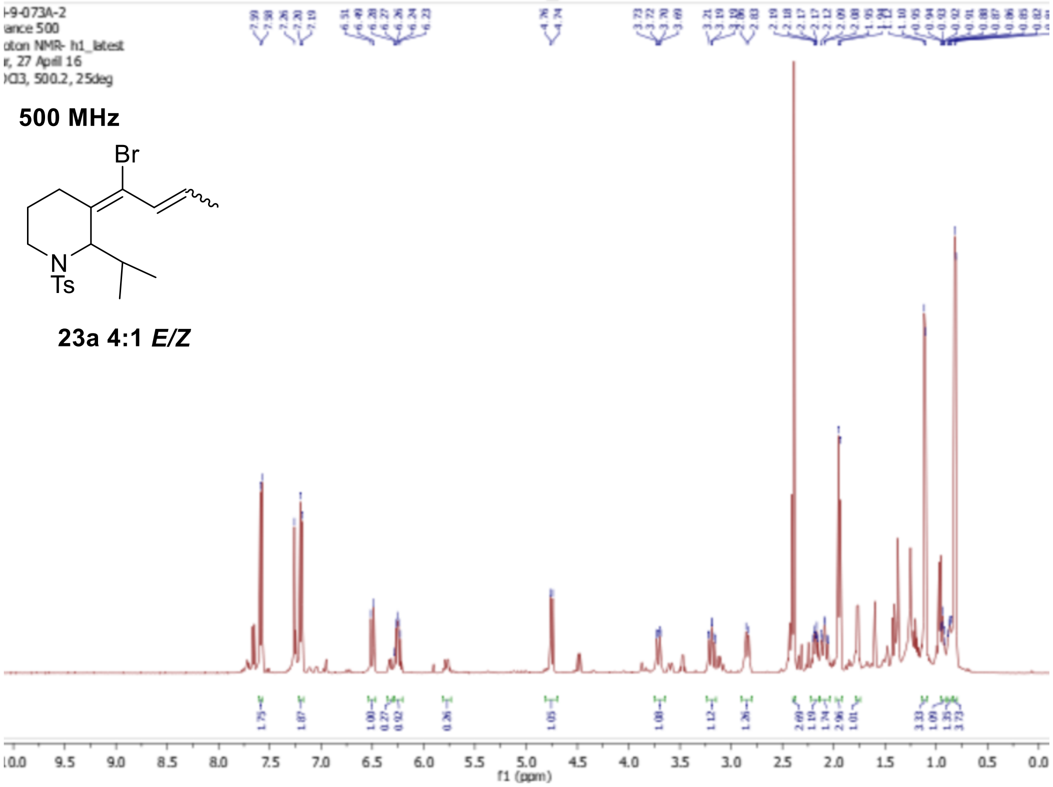
500 MHz
Br
N
Ts
23a 4:1 *E/Z*
f1 (ppm)

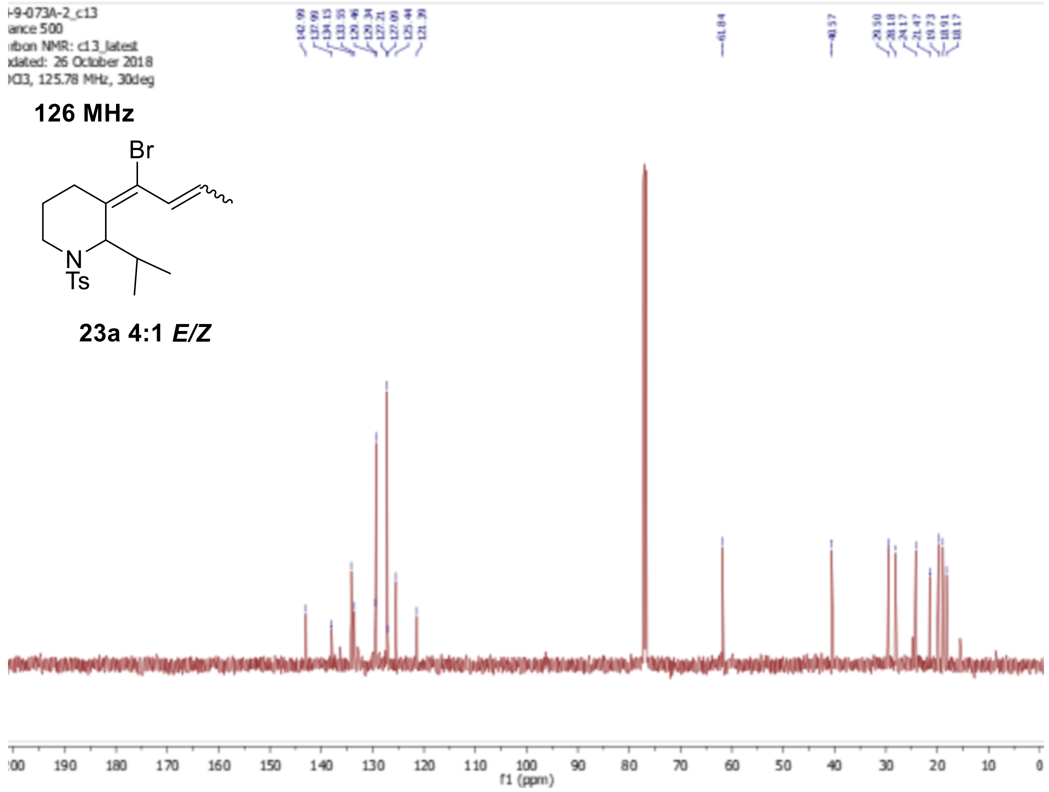
126 MHz
Br
N
Ts
23a 4:1 *E/Z*
f1 (ppm)

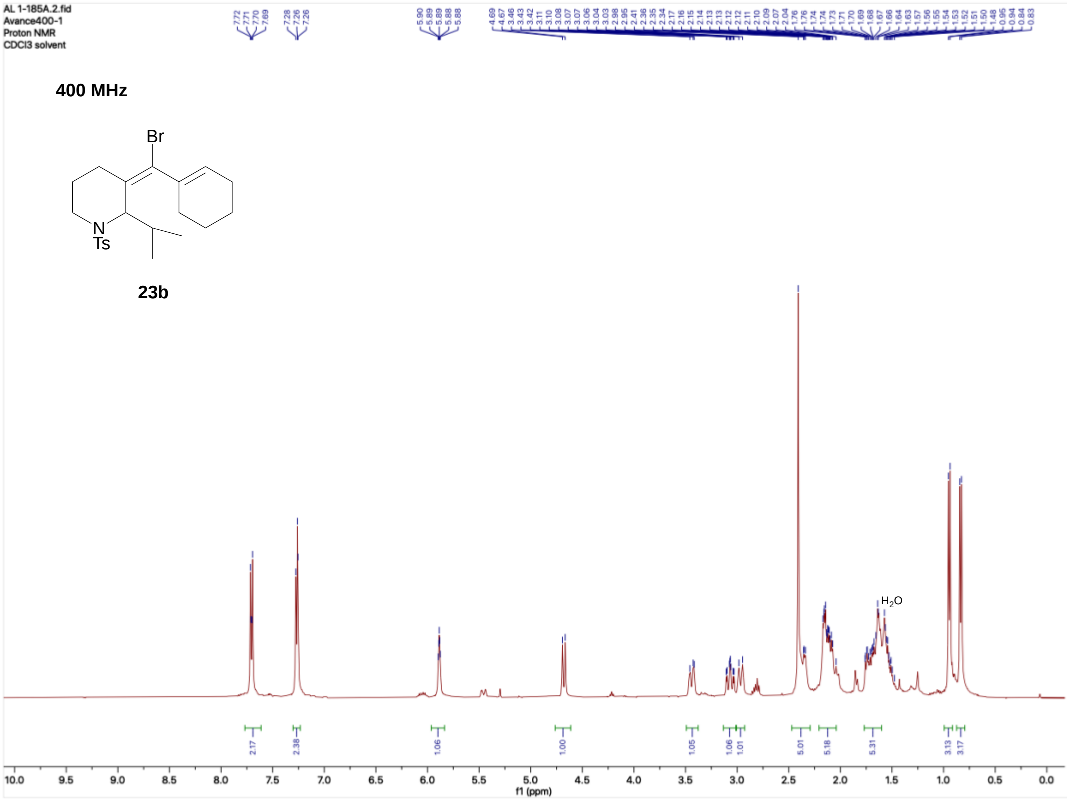
AL 1-185A.2.fid
Avance400-1
Proton NMR
CDCl3 solvent
7.72 7.71 7.70 7.69 7.28 7.26 7.26 5.90 5.89 5.89 5.88 5.88 4.69 4.67 3.46 3.43 3.42 3.11 3.10 3.08 3.07 3.06 3.04 3.03 2.98 2.95 2.41 2.36 2.35 2.34 2.17 2.16 2.15 2.14 2.13 2.12 2.12 2.11 2.10 2.09 2.07 2.04 1.76 1.76 1.74 1.74 1.73 1.71 1.70 1.69 1.68 1.67 1.66 1.64 1.63 1.57 1.56 1.55 1.54 1.53 1.52 1.51 1.50 1.48 0.95 0.94 0.84 0.83
400 MHz
Br
N
Ts
23b
H_2O
2.17 2.38 1.06 1.00 1.05 1.06 1.01 5.01 5.18 5.31 3.13 3.17
10.0 9.5 9.0 8.5 8.0 7.5 7.0 6.5 6.0 5.5 5.0 4.5 4.0 3.5 3.0 2.5 2.0 1.5 1.0 0.5 0.0
f1 (ppm)

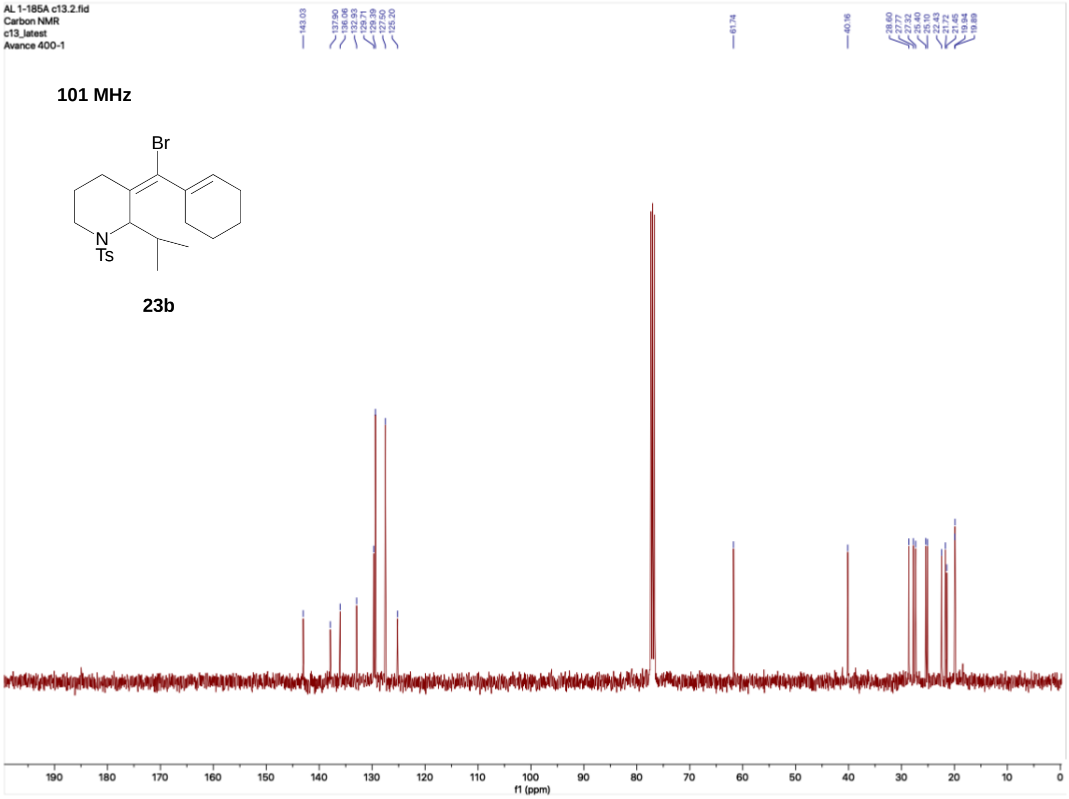
AL 1-185A c13.2.fid
Carbon NMR
c13_latest
Avance 400-1
143.03 137.90 136.06 132.93 129.71 129.39 127.50 125.20 61.74 40.16 28.60 27.77 27.32 25.40 25.10 22.43 21.72 21.45 19.94 19.89
101 MHz
Br
N
Ts
23b
190 180 170 160 150 140 130 120 110 100 90 80 70 60 50 40 30 20 10 0
f1 (ppm)

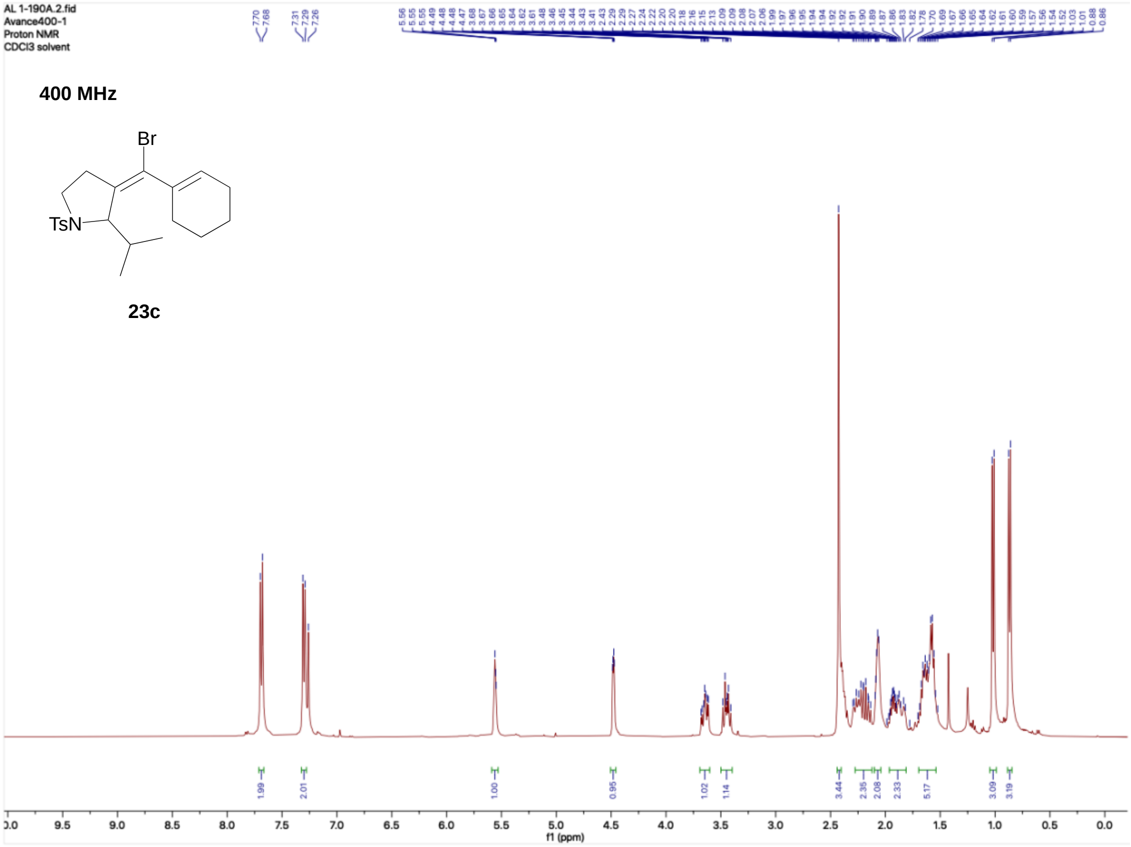
AL 1-190A.2.fid
Avance400-1
Proton NMR
CDCl3 solvent
400 MHz
Br
TsN
23c
f1 (ppm)

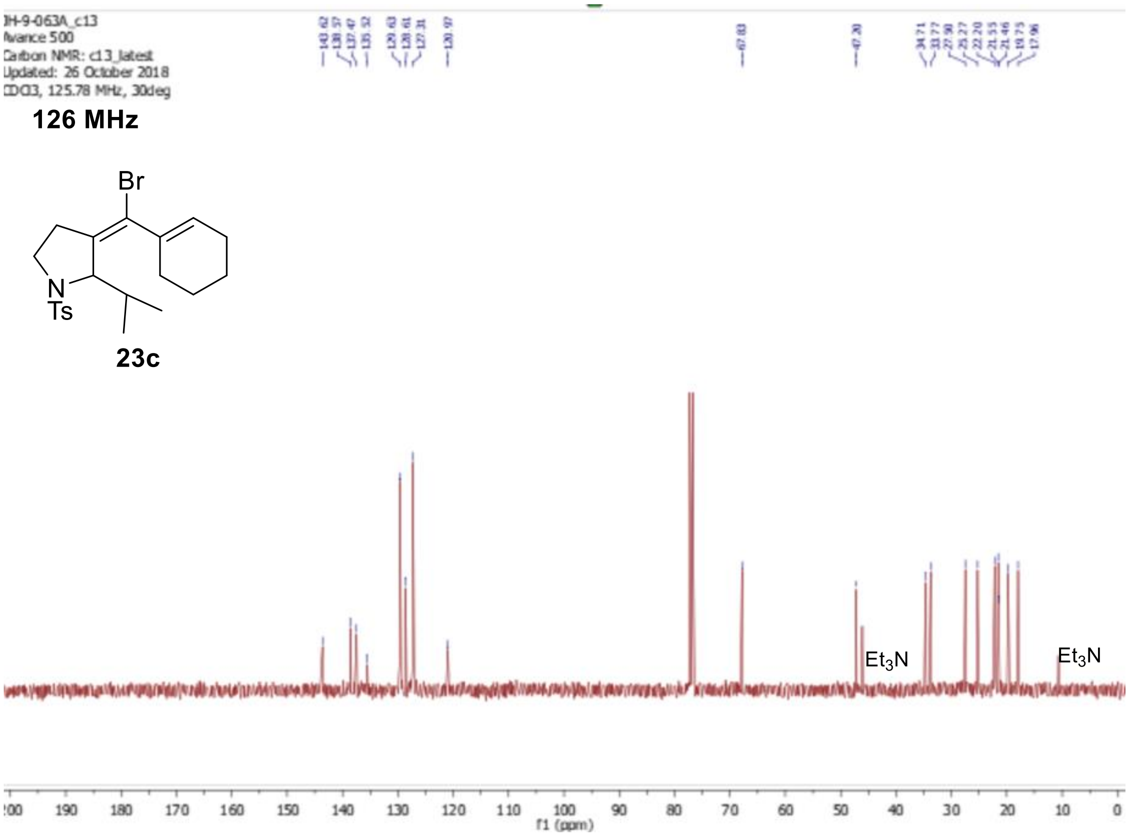
Avance 500
Updated: 26 October 2018
126 MHz
Br
N
Ts
23c
Et3N
Et3N
f1 (ppm)

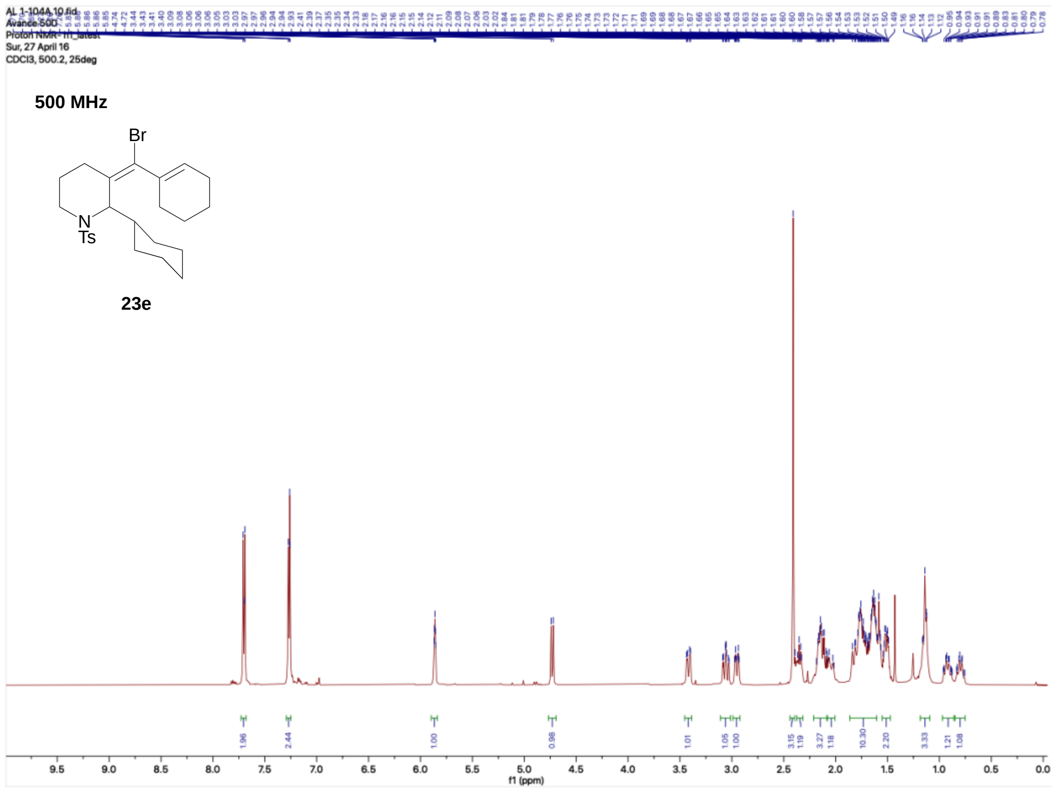
AL 1-104A.10.fid
Avance 500
Sur, 27 April 16
CDCl3, 500.2, 25deg
500 MHz
Br
N
Ts
23e
f1 (ppm)

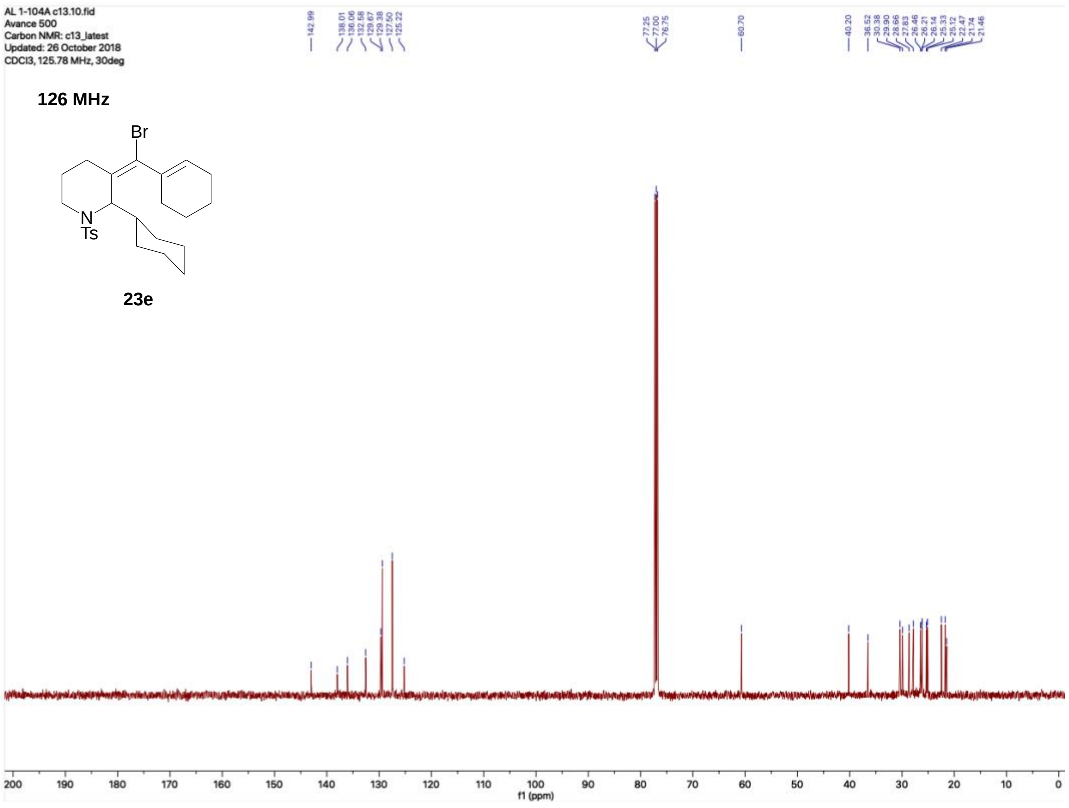
AL 1-104A c13.10.fid
Avance 500
Carbon NMR: c13_latest
Updated: 26 October 2018
CDCl3, 125.78 MHz, 30deg
126 MHz
Br
N
Ts
23e
f1 (ppm)

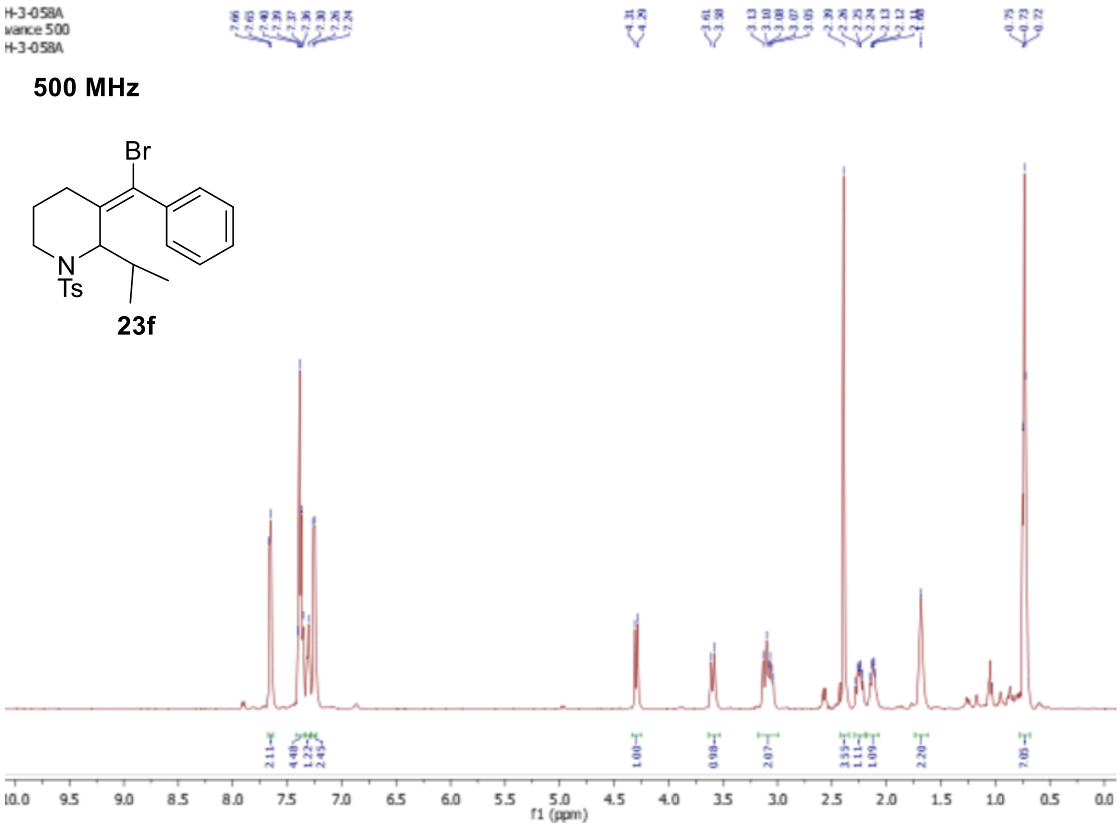

500 MHz
Br
N
Ts
23f
f1 (ppm)

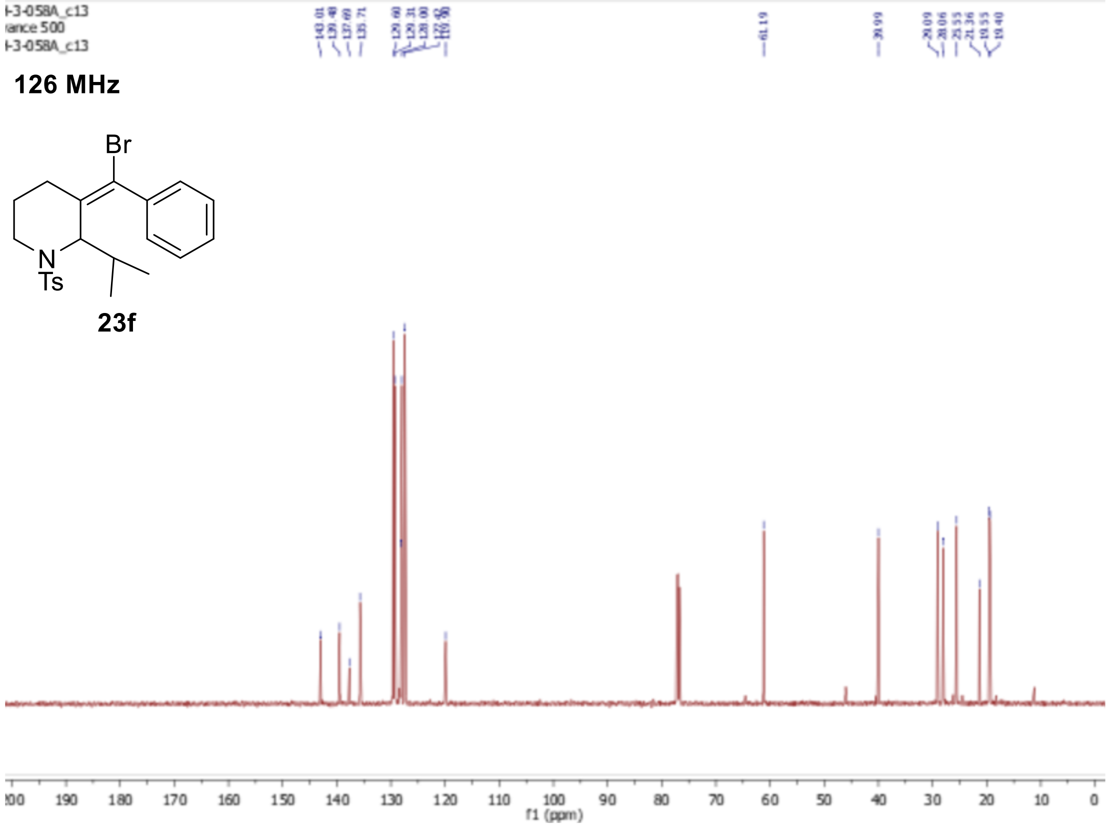

126 MHz
Br
N
Ts
23f
f1 (ppm)

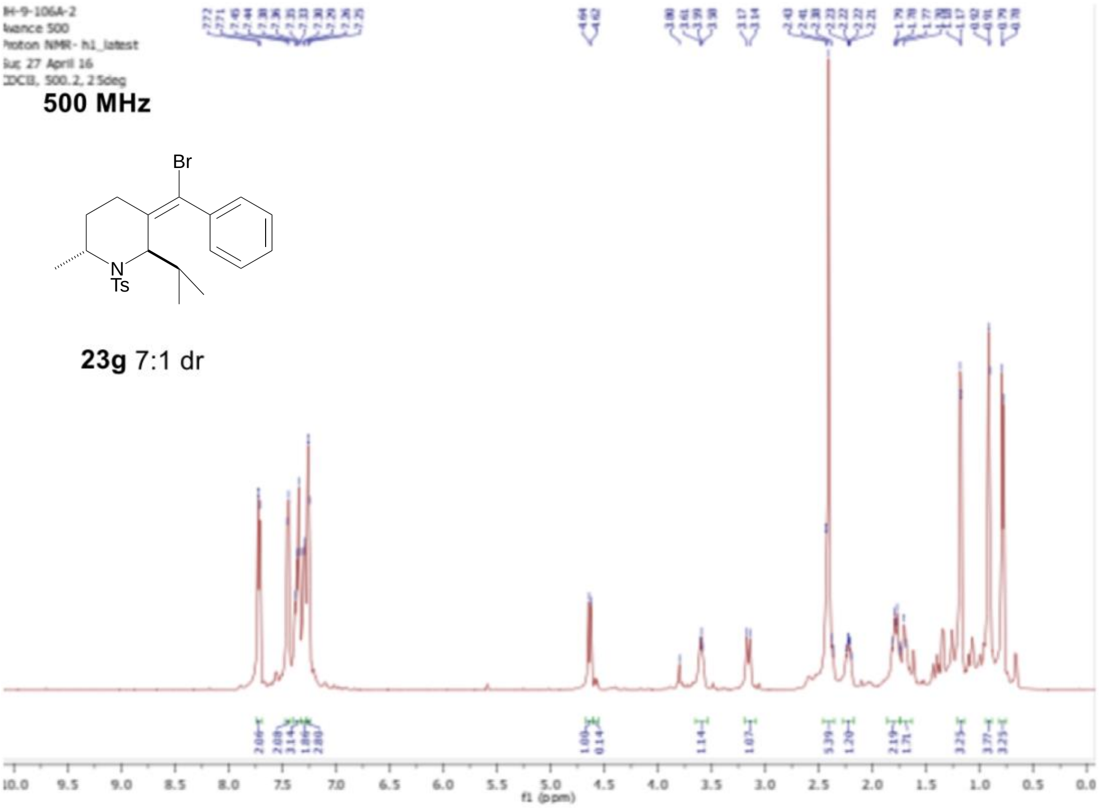
500 MHz
Br
N
Ts
23g 7:1 dr
f1 (ppm)

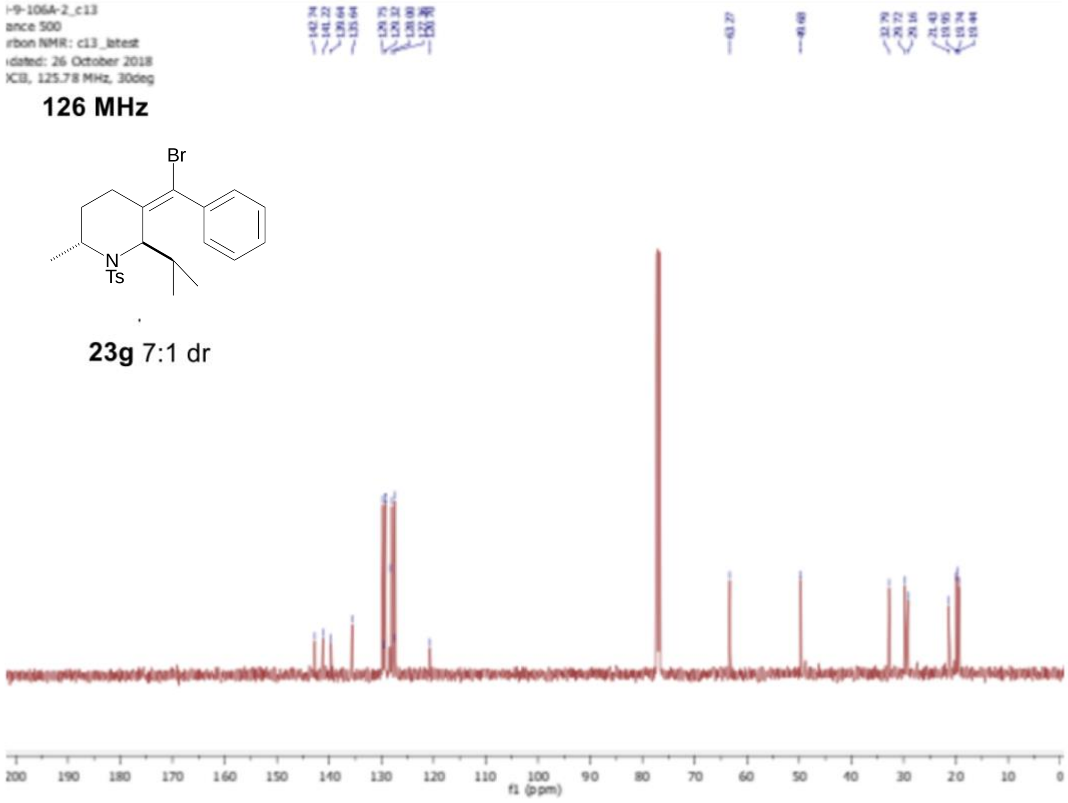
126 MHz
Br
N
Ts
23g 7:1 dr
f1 (ppm)

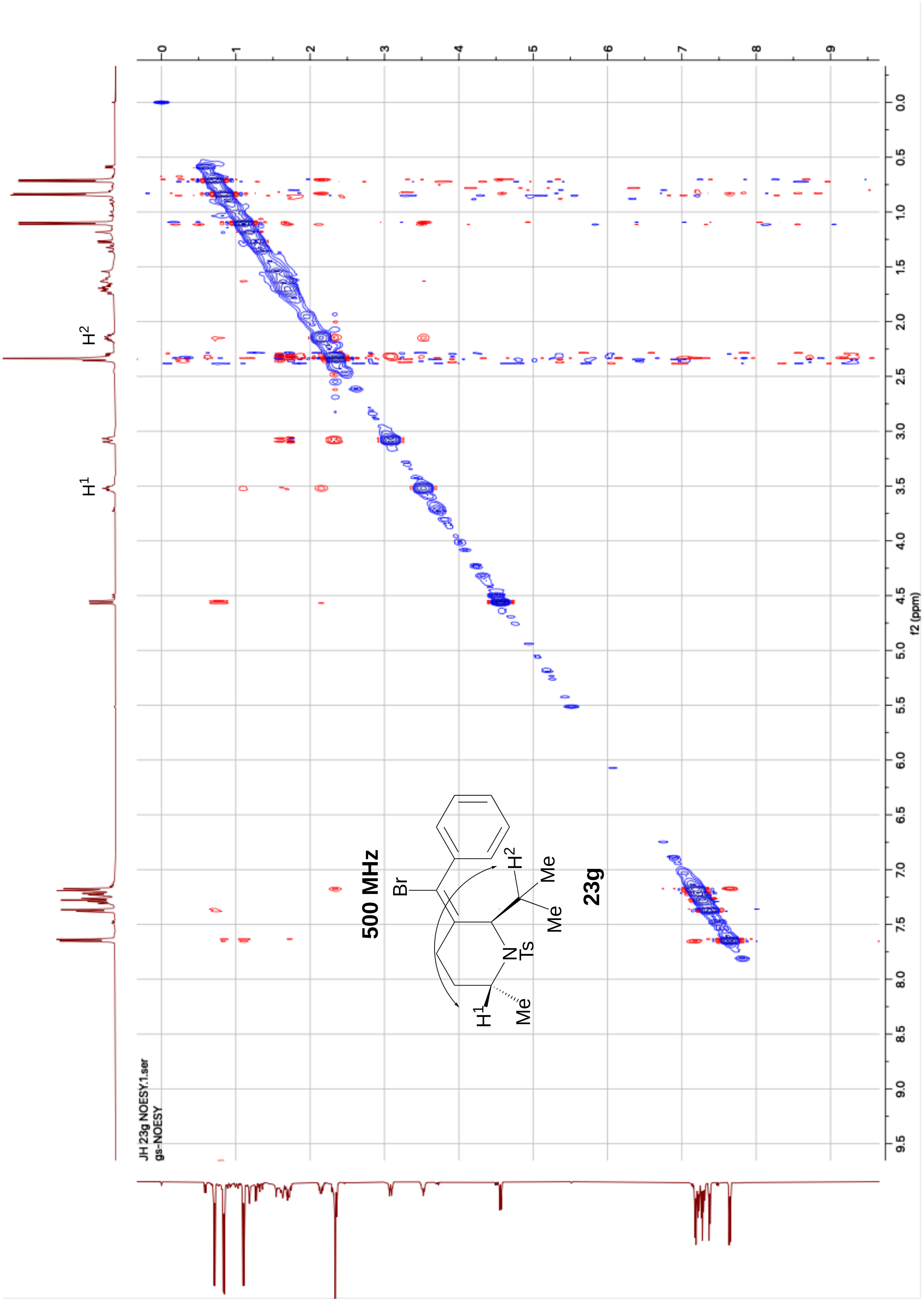
JH 23g NOESY1.ser
gs-NOESY
500 MHz
Br
H2
Me
Me
N
Ts
H1
Me
23g
H1
H2
f2 (ppm)

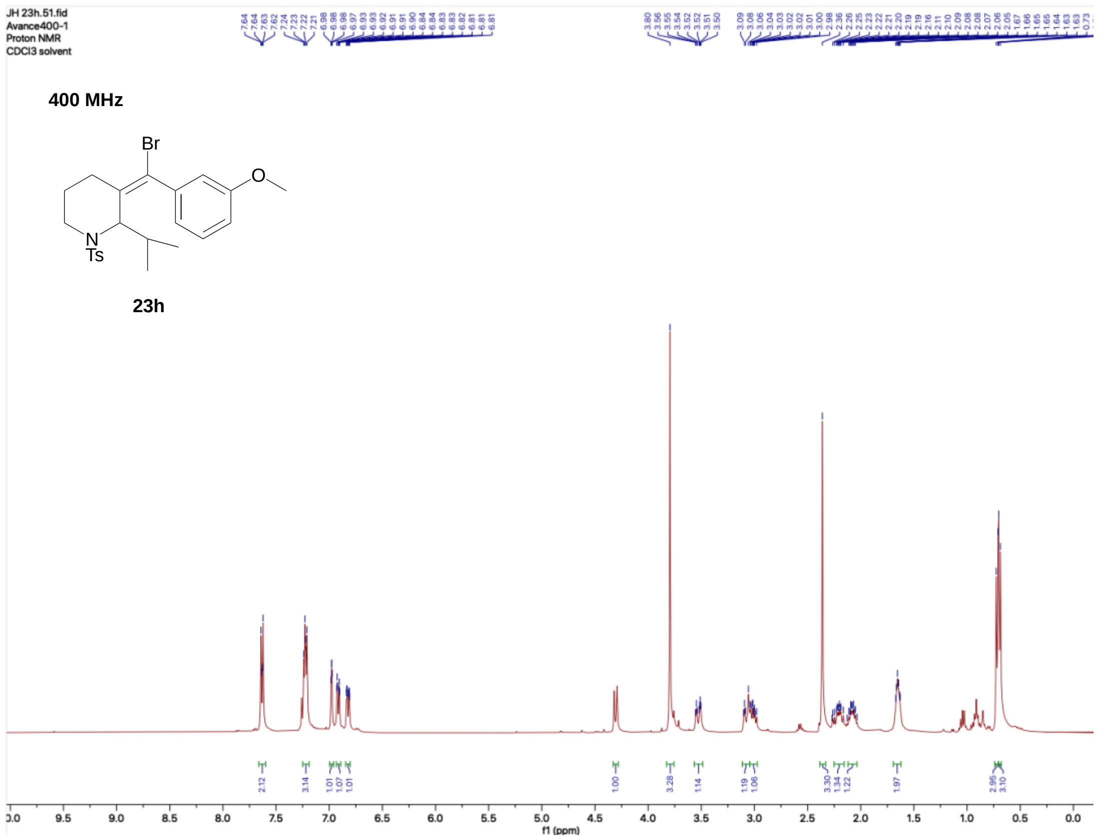
JH 23h.51.fid
Avance400-1
Proton NMR
CDCl3 solvent
400 MHz
Br
O
N
Ts
23h
2.12
3.14
1.01
1.07
1.01
1.00
3.28
1.14
1.19
1.06
3.30
1.34
1.22
1.97
2.95
3.10
f1 (ppm)

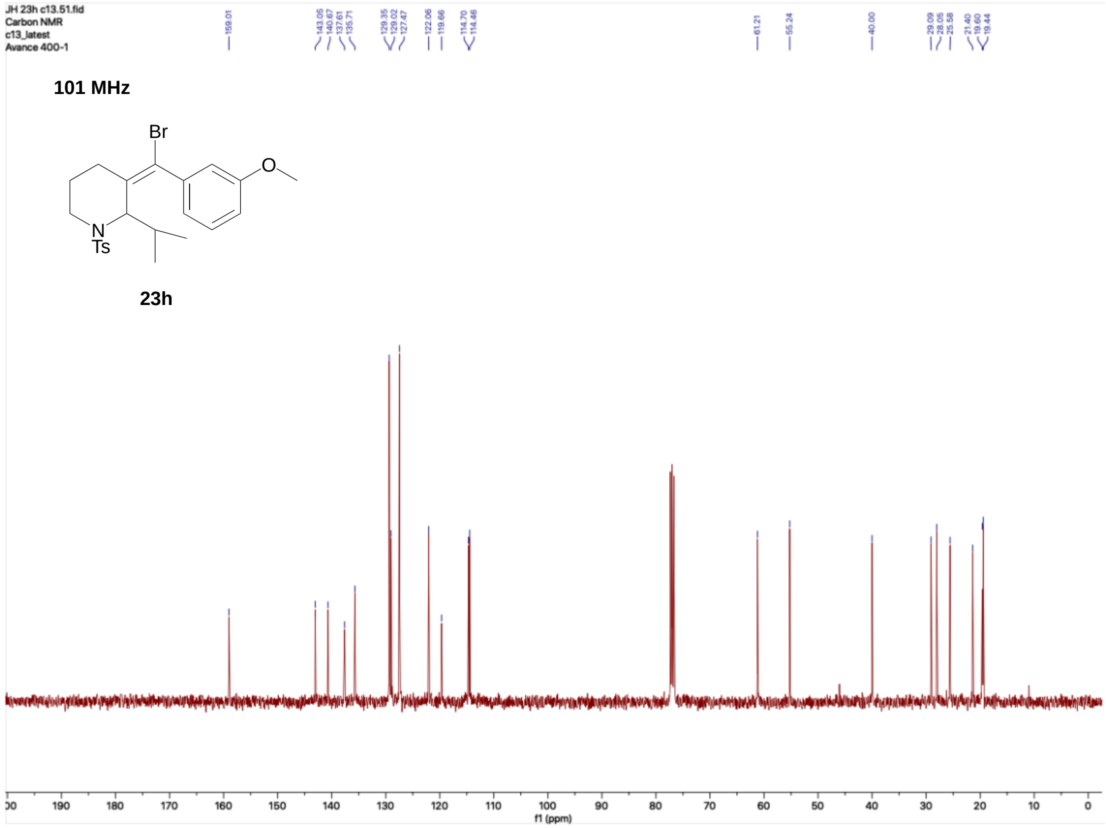
JH 23h c13.51.fid
Carbon NMR
c13_latest
Avance 400-1
159.01
143.05
140.67
137.61
135.71
129.35
129.02
127.47
122.06
119.66
114.70
114.46
61.21
55.24
40.00
29.09
28.05
25.58
21.40
19.60
19.44
101 MHz
Br
O
N
Ts
23h
f1 (ppm)

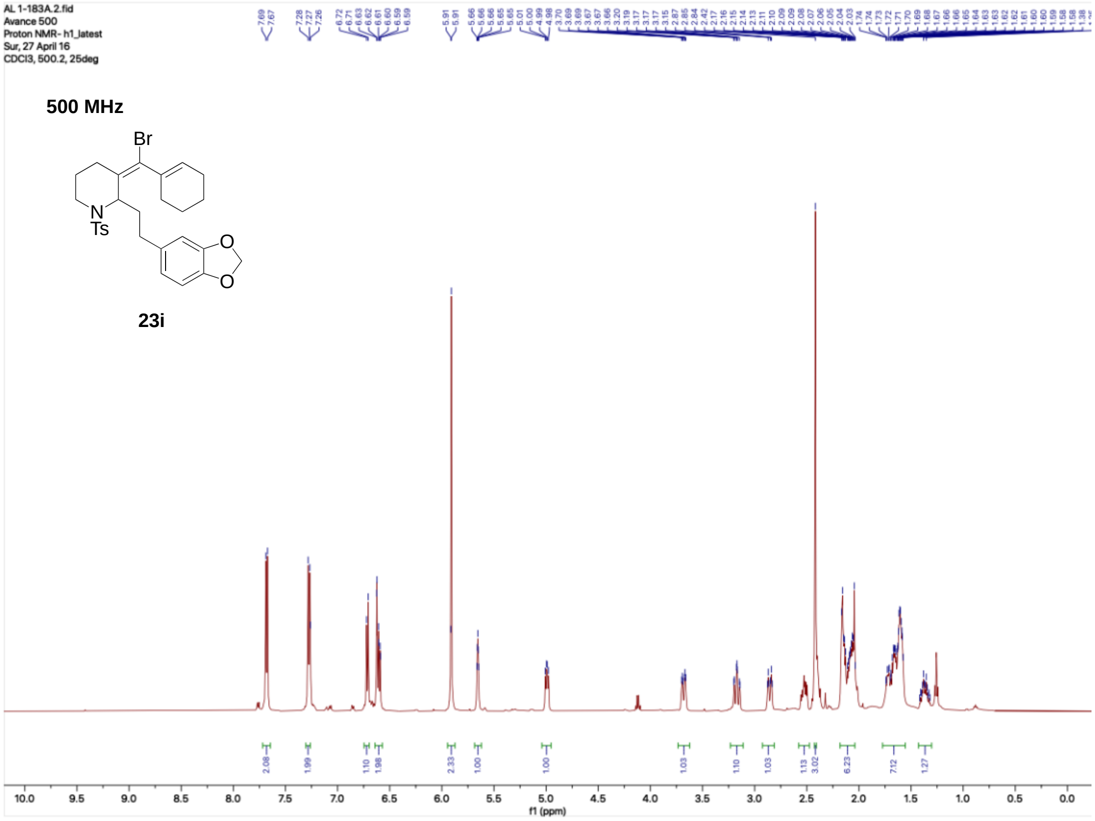
AL 1-183A.2.fid
Avance 500
Proton NMR- h1_latest
Sur, 27 April 16
CDCl3, 500.2, 25deg
500 MHz
Br
N
Ts
O
O
23i
f1 (ppm)

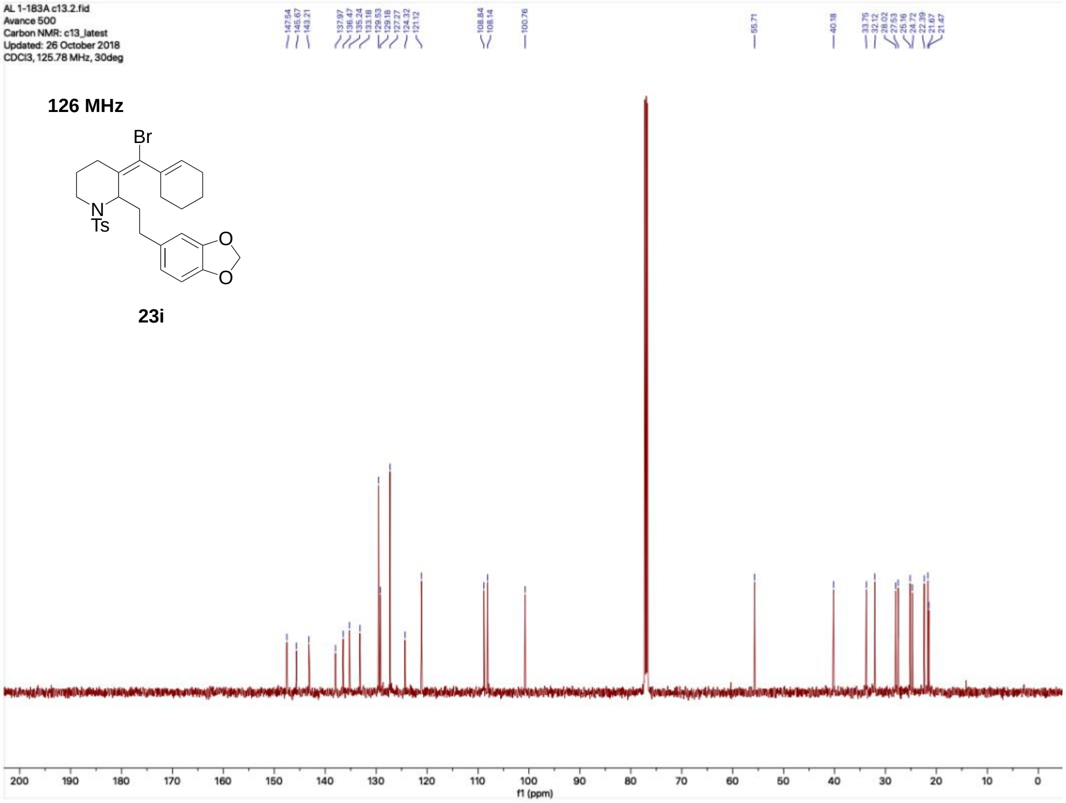
AL 1-183A c13.2.fid
Avance 500
Carbon NMR: c13_latest
Updated: 26 October 2018
CDCl3, 125.78 MHz, 30deg
147.54
145.67
143.21
137.97
136.47
135.24
133.18
129.53
129.18
127.27
124.32
121.12
108.84
108.14
100.76
55.71
40.18
33.75
32.12
28.02
27.53
25.16
24.72
22.39
21.67
21.47
126 MHz
Br
N
Ts
O
O
23i
f1 (ppm)

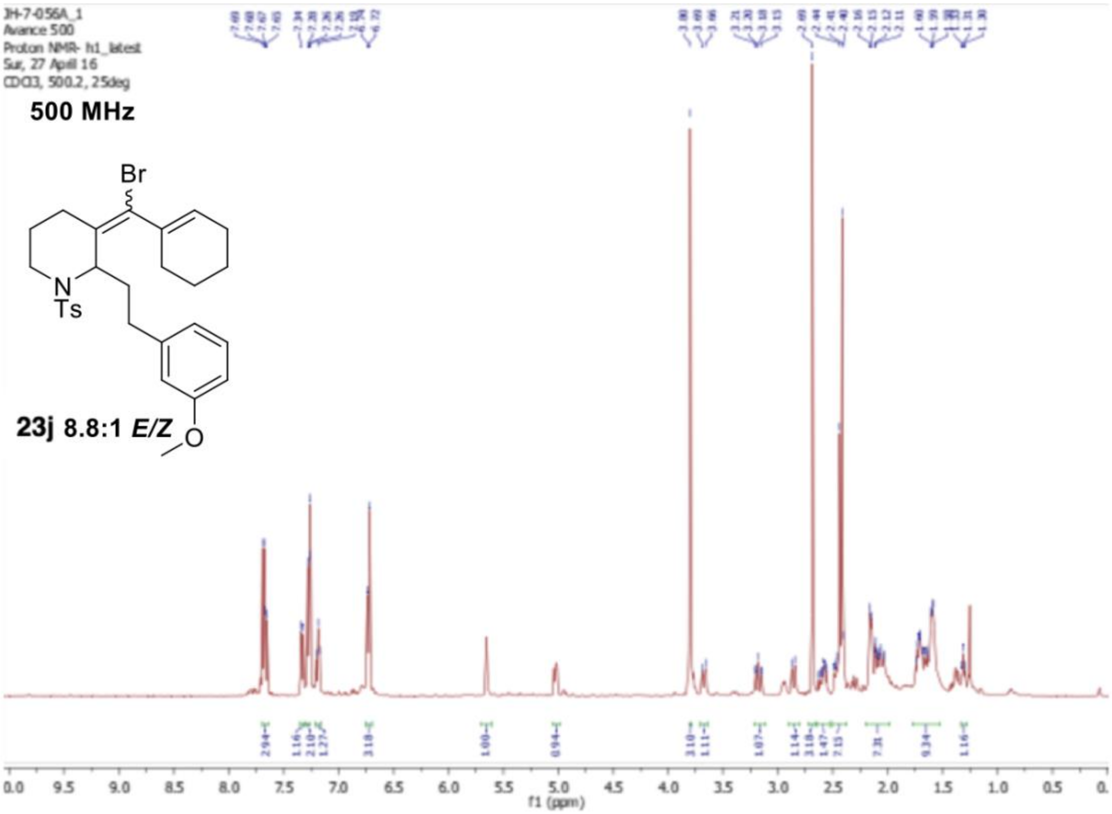
JH-7-056A_1
Avance 500
Proton NMR- h1_latest
CDCl3, 500.2, 25deg
500 MHz
Br
N
Ts
O
23j 8.8:1 E/Z
f1 (ppm)

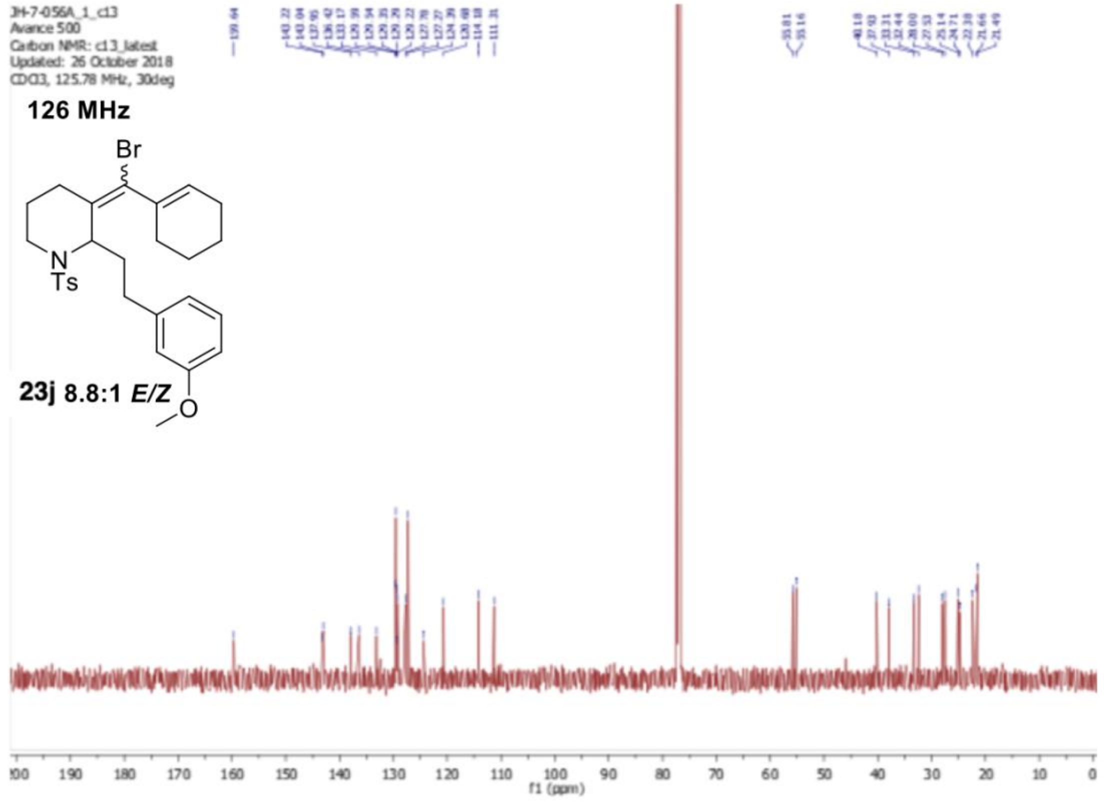
JH-7-056A_1_c13
Avance 500
Carbon NMR: c13_latest
Updated: 26 October 2018
CDCl3, 125.78 MHz, 30deg
126 MHz
Br
N
Ts
O
23j 8.8:1 E/Z
f1 (ppm)

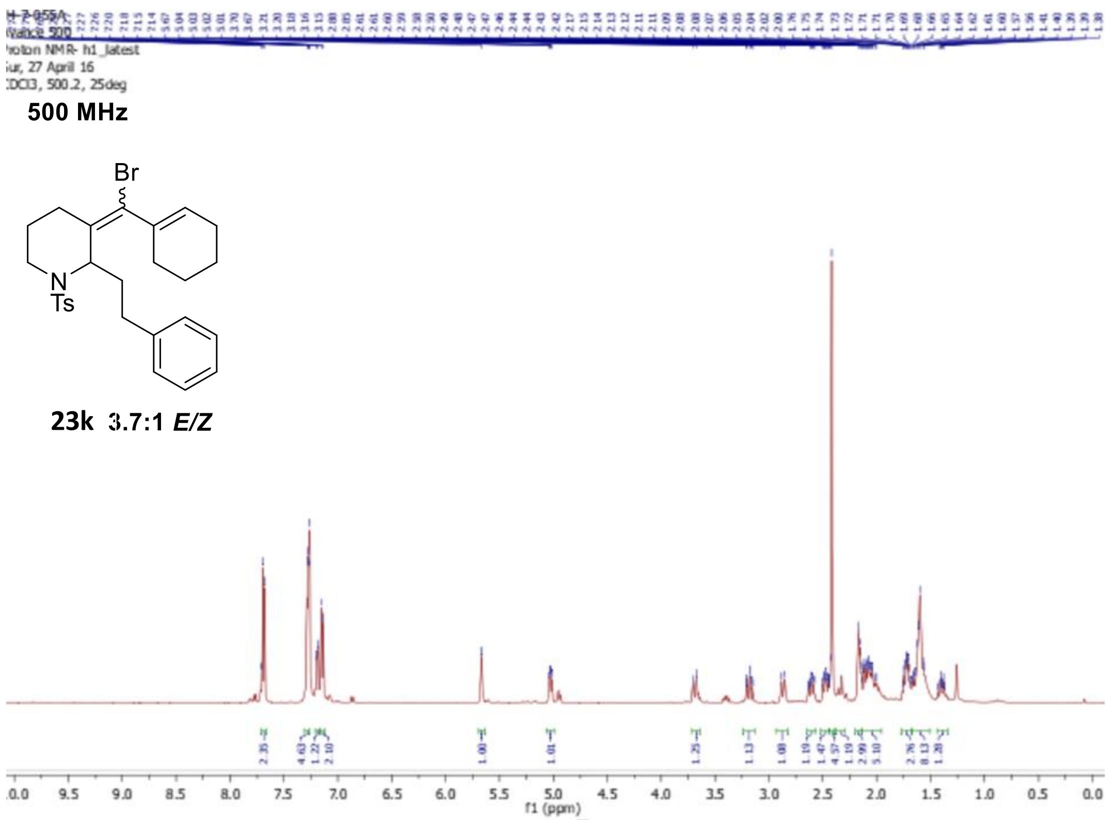
500 MHz
Br
N
Ts
23k 3.7:1 E/Z
f1 (ppm)

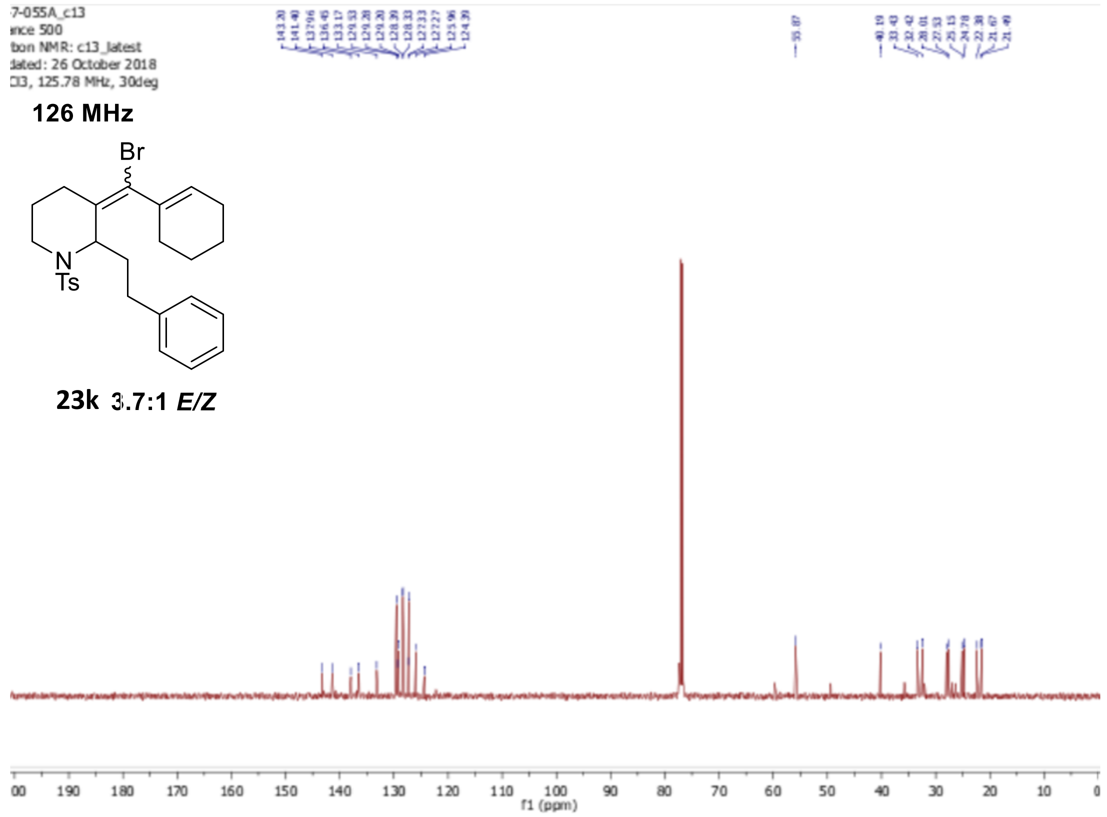
126 MHz
Br
N
Ts
23k 3.7:1 E/Z
f1 (ppm)

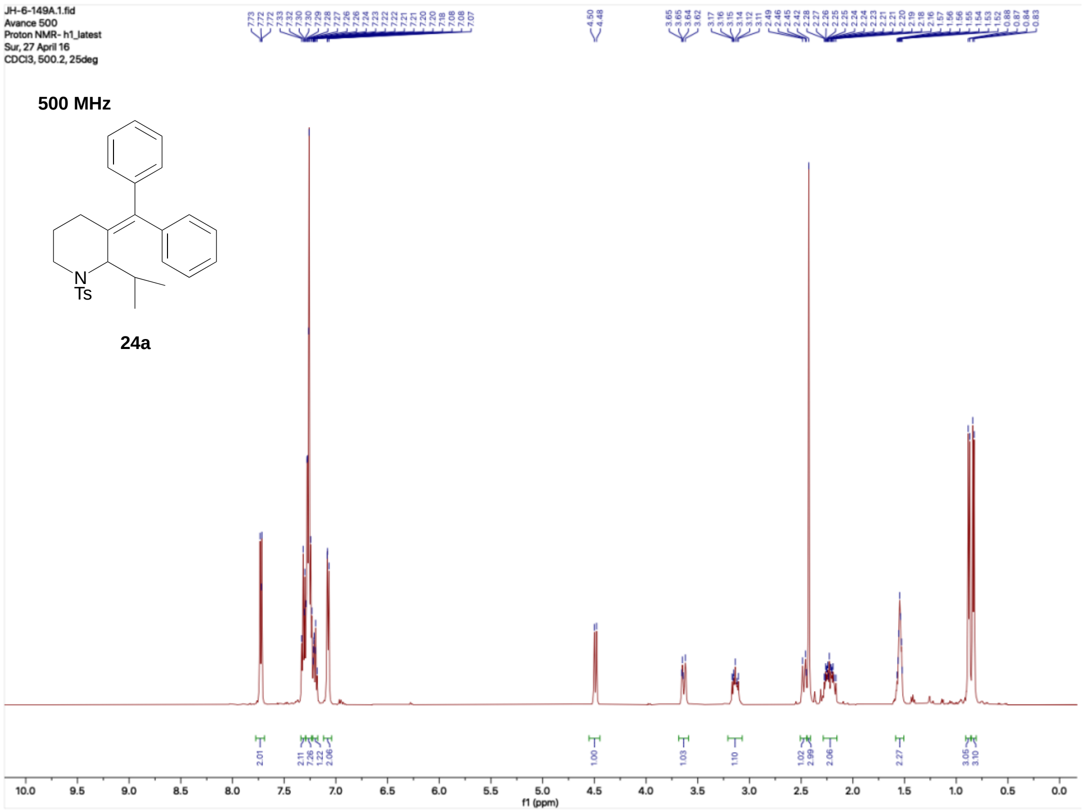
JH-6-149A.1.fid
Avance 500
Proton NMR- h1_latest
Sur, 27 April 16
CDCl3, 500.2, 25deg
500 MHz
N
Ts
24a
f1 (ppm)

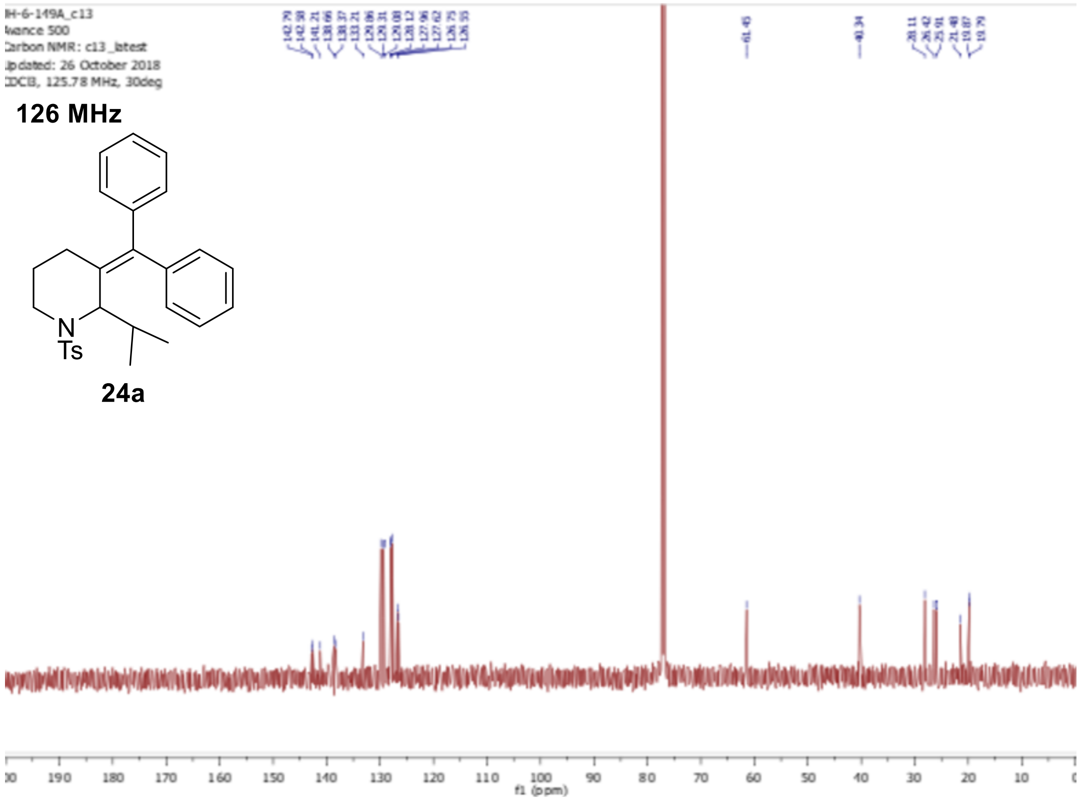
H-6-149A_c13
vance 500
arbon NMR: c13_latest
pdated: 26 October 2018
DCl3, 125.78 MHz, 30deg
126 MHz
N
Ts
24a
f1 (ppm)

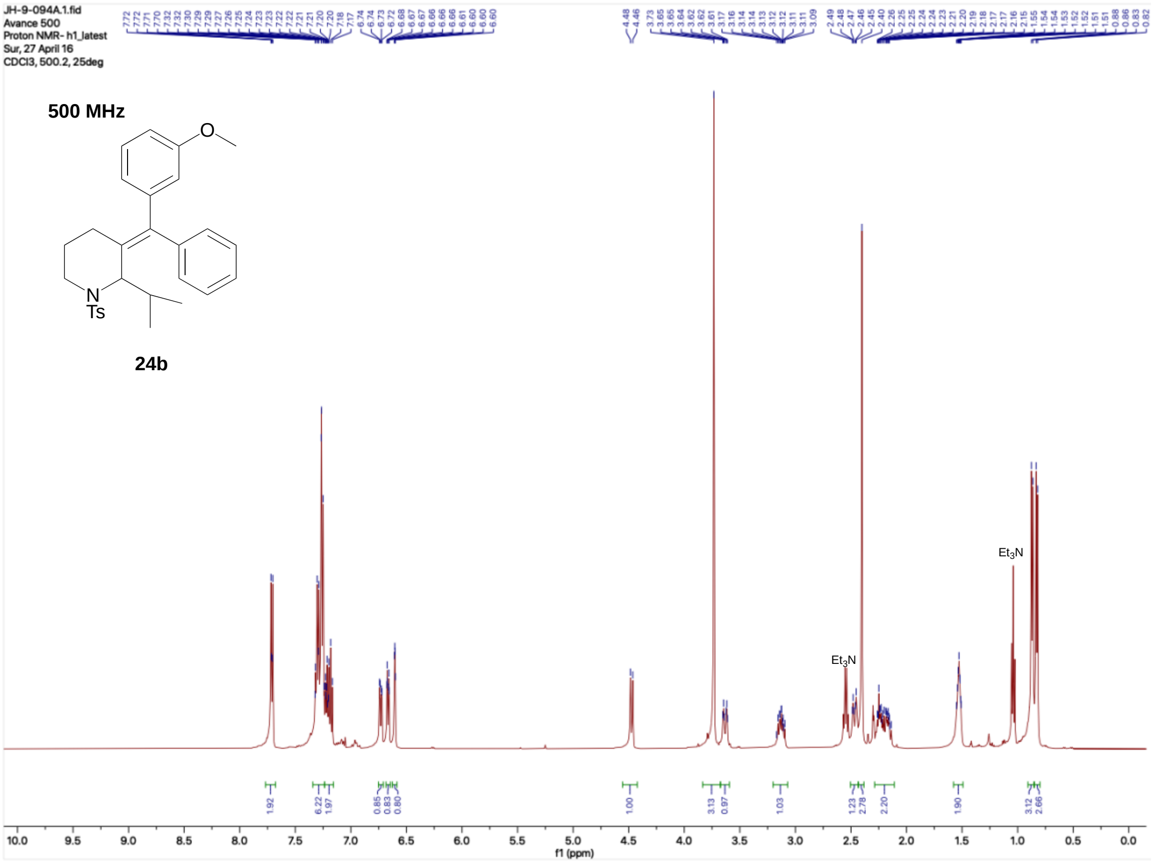
JH-9-094A.1.fid
Avance 500
Proton NMR- h1_latest
Sur, 27 April 16
CDCl3, 500.2, 25deg
500 MHz
24b
Et3N
Et3N
f1 (ppm)

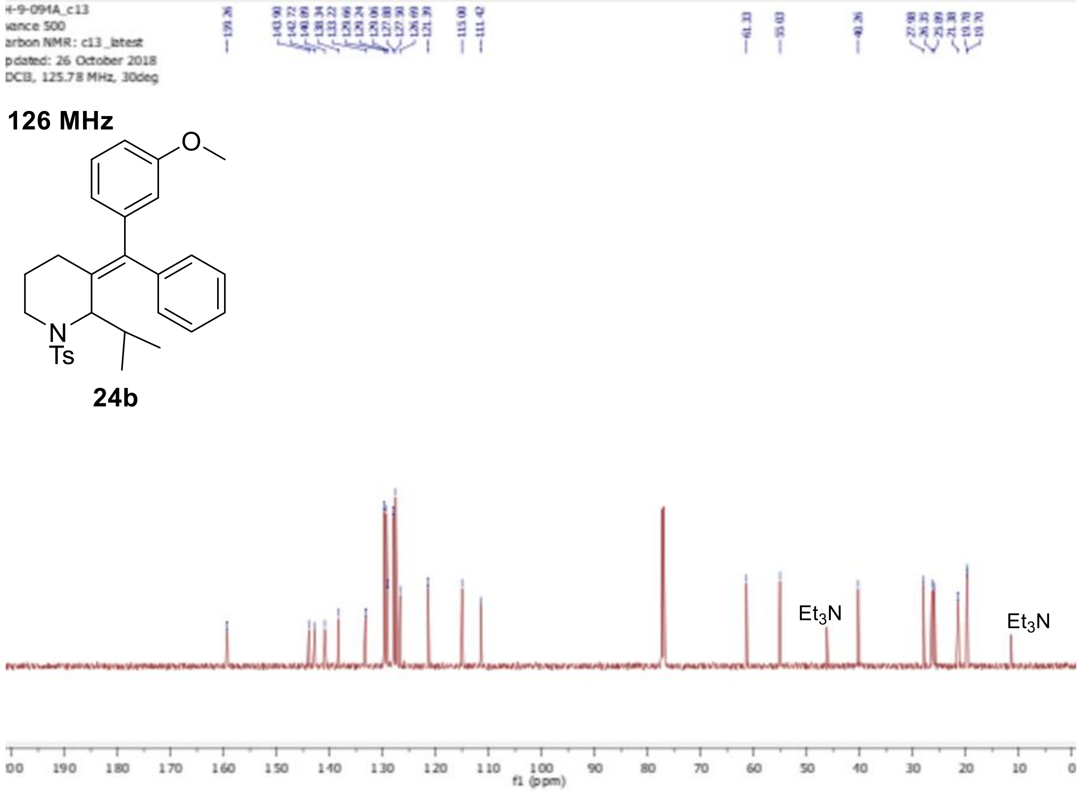
126 MHz
24b
Et3N
Et3N
f1 (ppm)

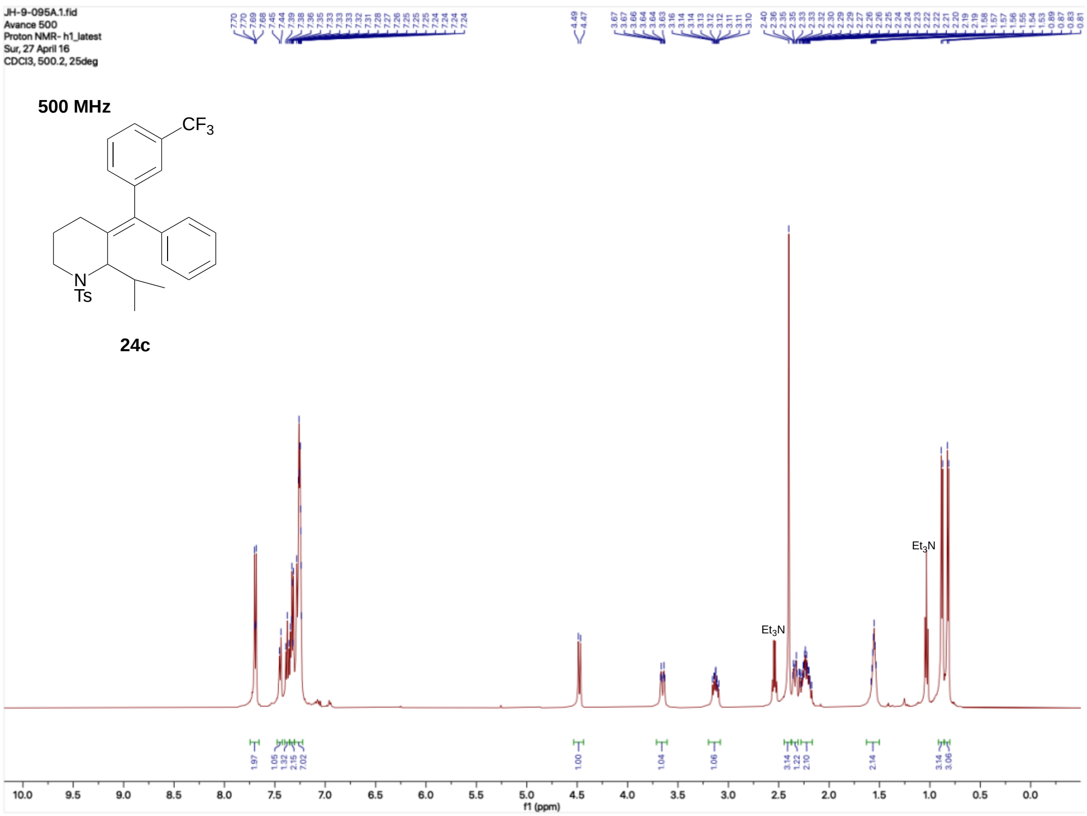
JH-9-095A.1.fid
Avance 500
Proton NMR- h1_latest
Sur, 27 April 16
CDCl3, 500.2, 25deg
500 MHz
CF3
N
Ts
24c
Et3N
Et3N
f1 (ppm)

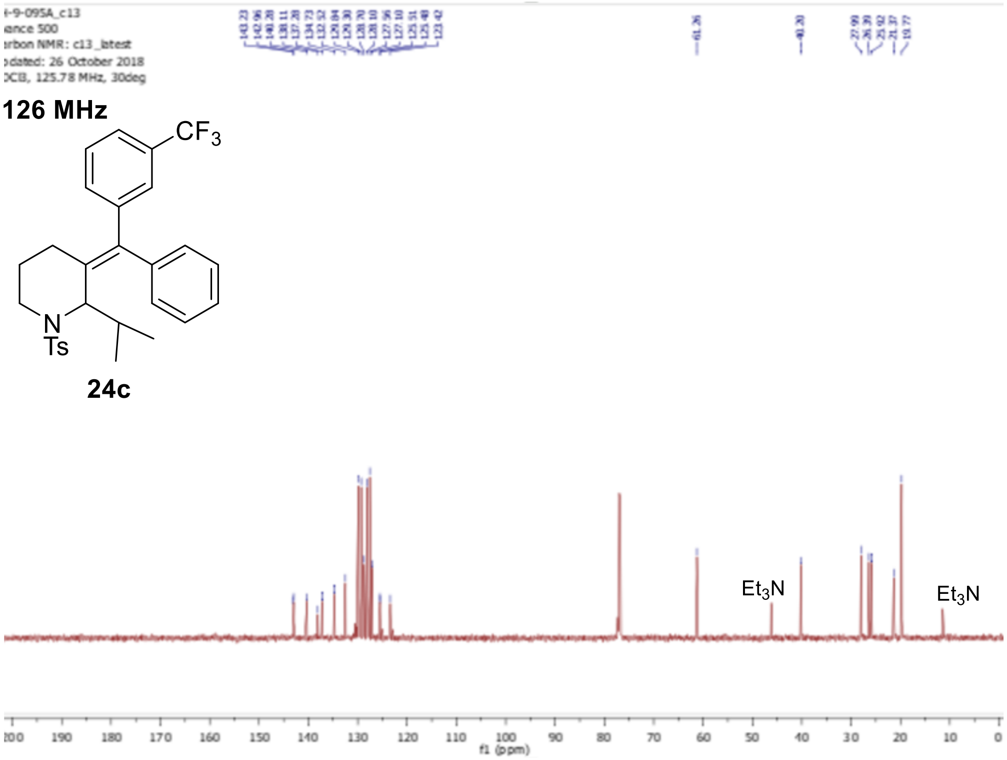
126 MHz
CF3
N
Ts
24c
Et3N
Et3N
f1 (ppm)

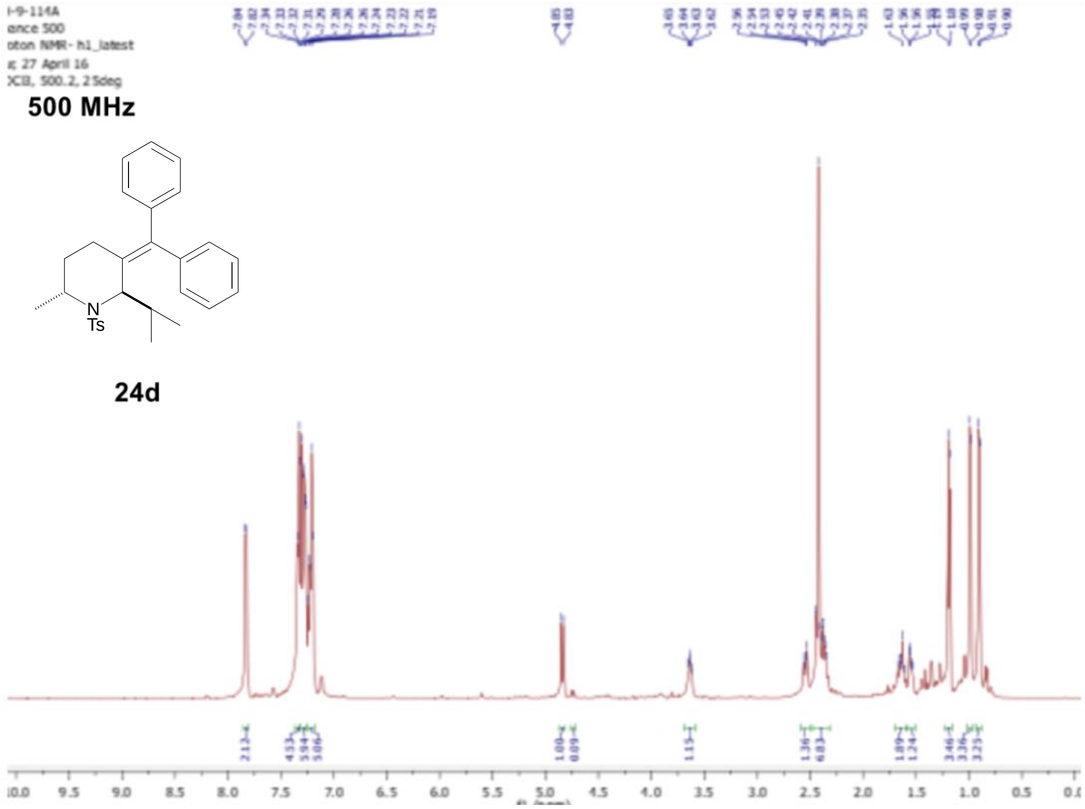
500 MHz
N
Ts
24d

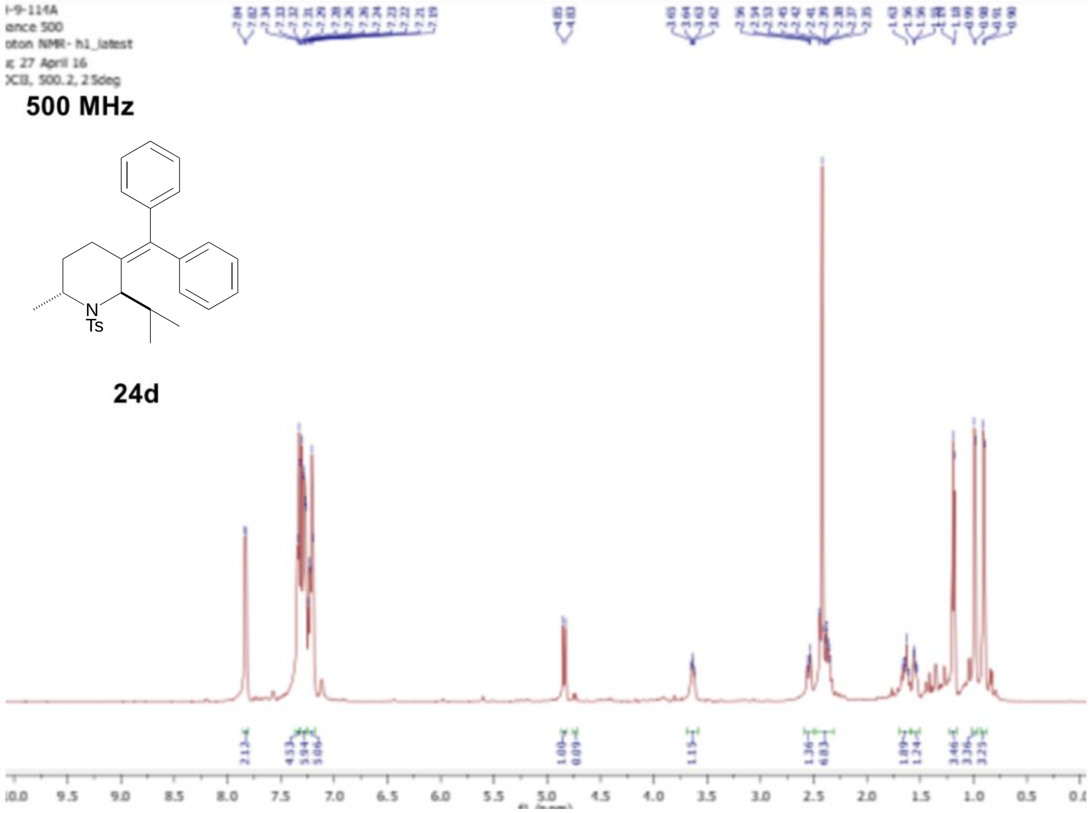
500 MHz
N
Ts
24d

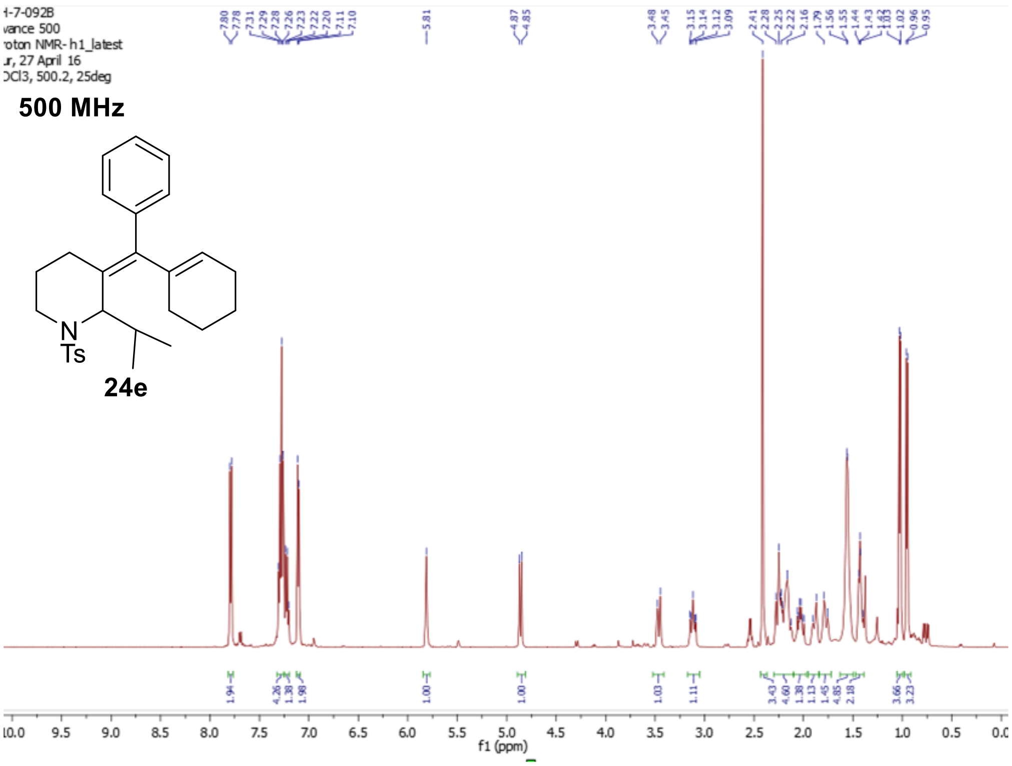
500 MHz
N
Ts
24e
f1 (ppm)

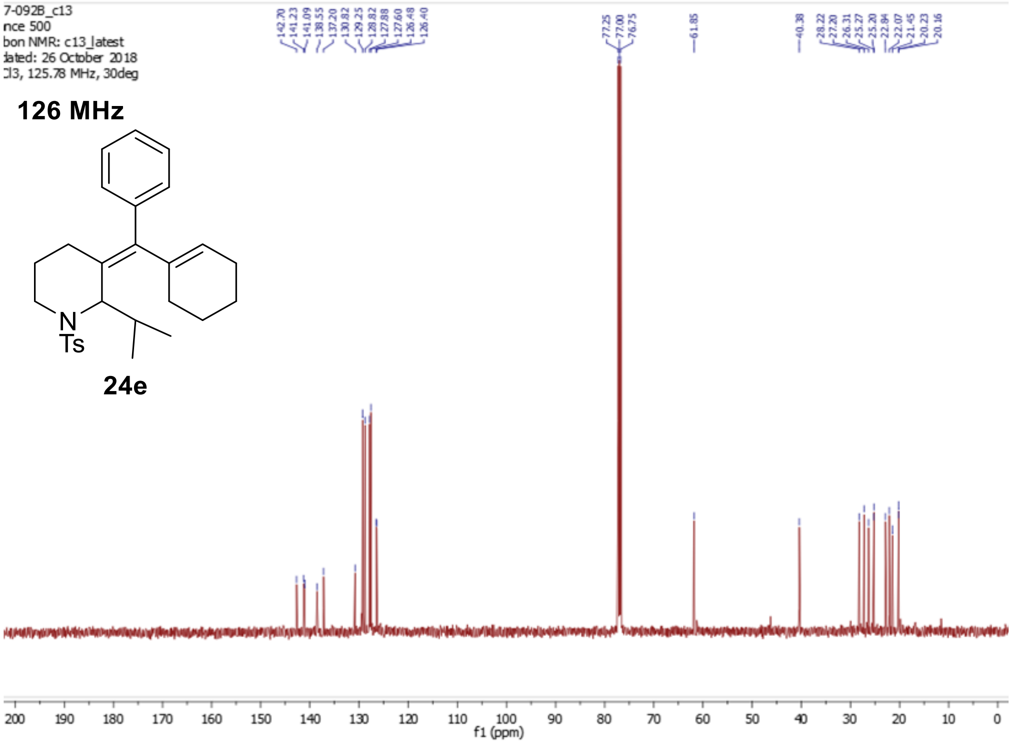
126 MHz
N
Ts
24e
f1 (ppm)

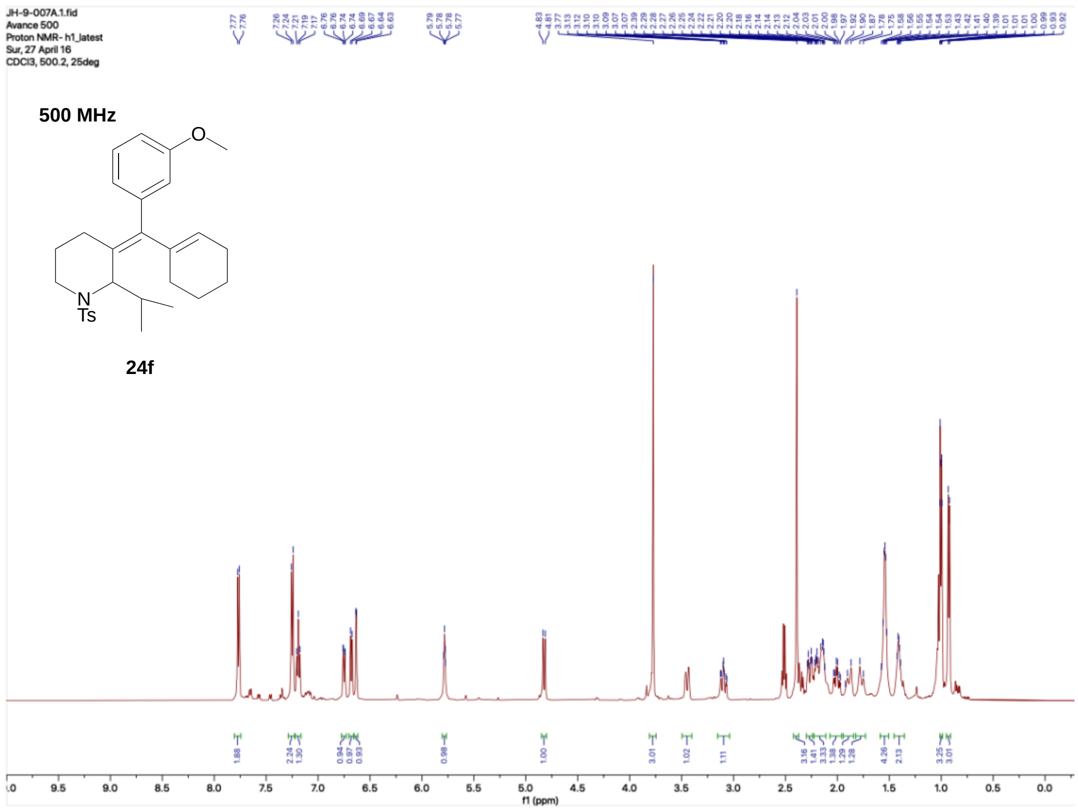
JH-9-007A.1.fid
Avance 500
Proton NMR- h1_latest
Sur, 27 April 16
CDCl3, 500.2, 25deg
500 MHz
O
N
Ts
24f
f1 (ppm)

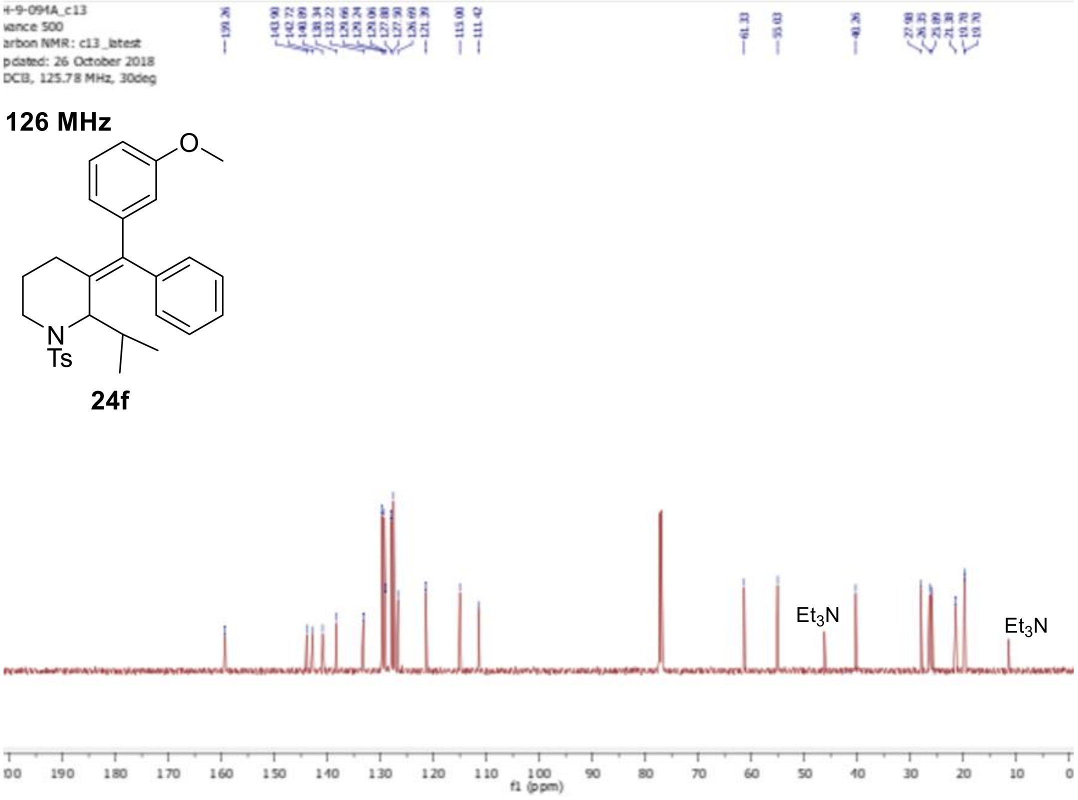
H-9-094A_c13
vance 500
arbon NMR: c13_latest
pdated: 26 October 2018
DCl3, 125.78 MHz, 30deg
126 MHz
O
N
Ts
24f
Et3N
Et3N
f1 (ppm)

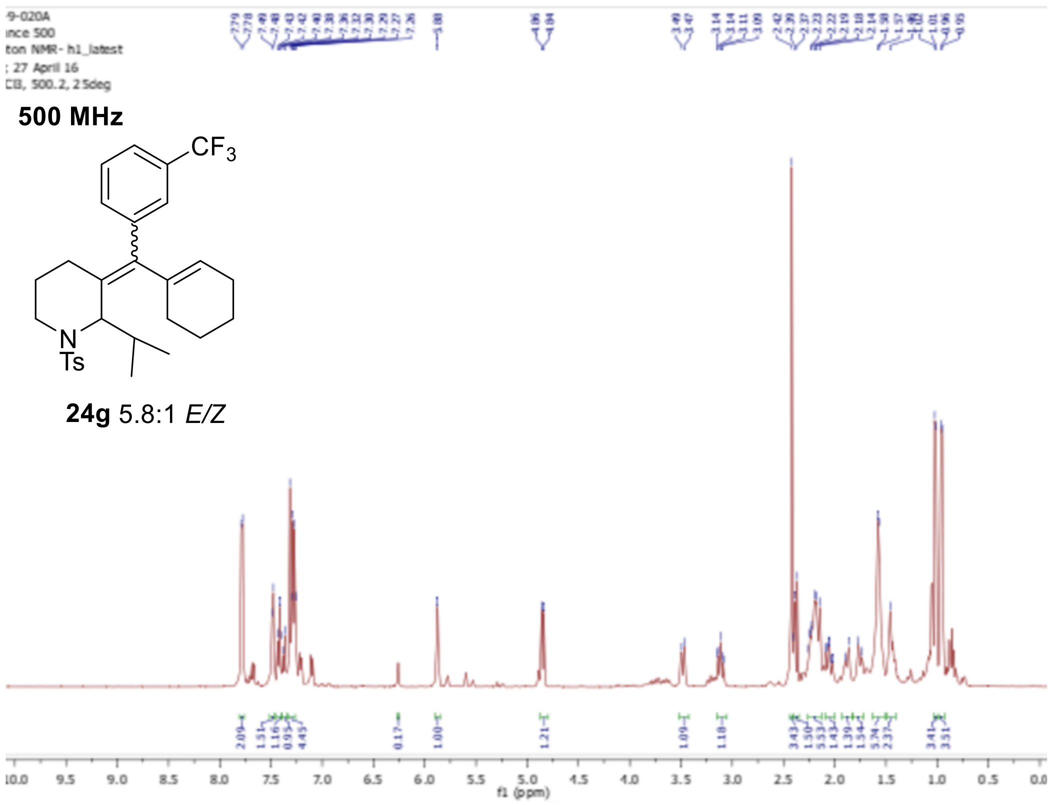
9-020A
nce 500
ton NMR- h1_latest
; 27 April 16
CB, 500.2, 25deg
500 MHz
CF3
N
Ts
24g 5.8:1 E/Z
f1 (ppm)

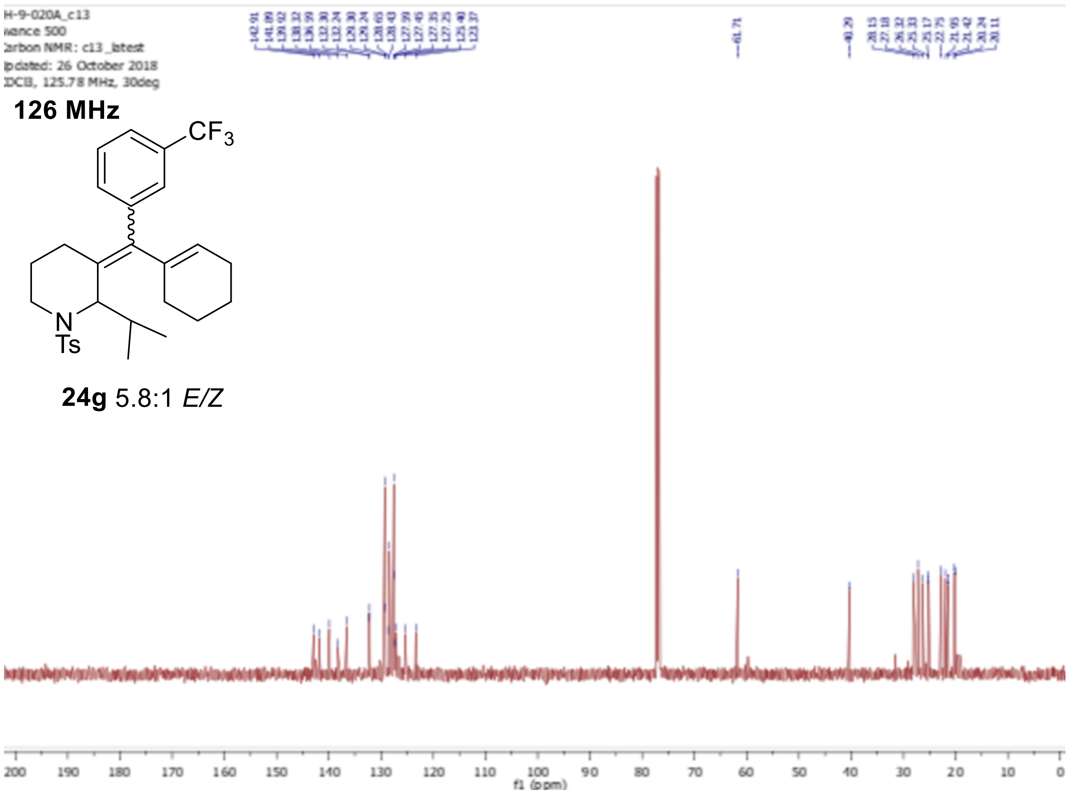
H-9-020A_c13
vance 500
Carbon NMR: c13_latest
Updated: 26 October 2018
DCB, 125.78 MHz, 30deg
126 MHz
CF3
N
Ts
24g 5.8:1 E/Z
f1 (ppm)

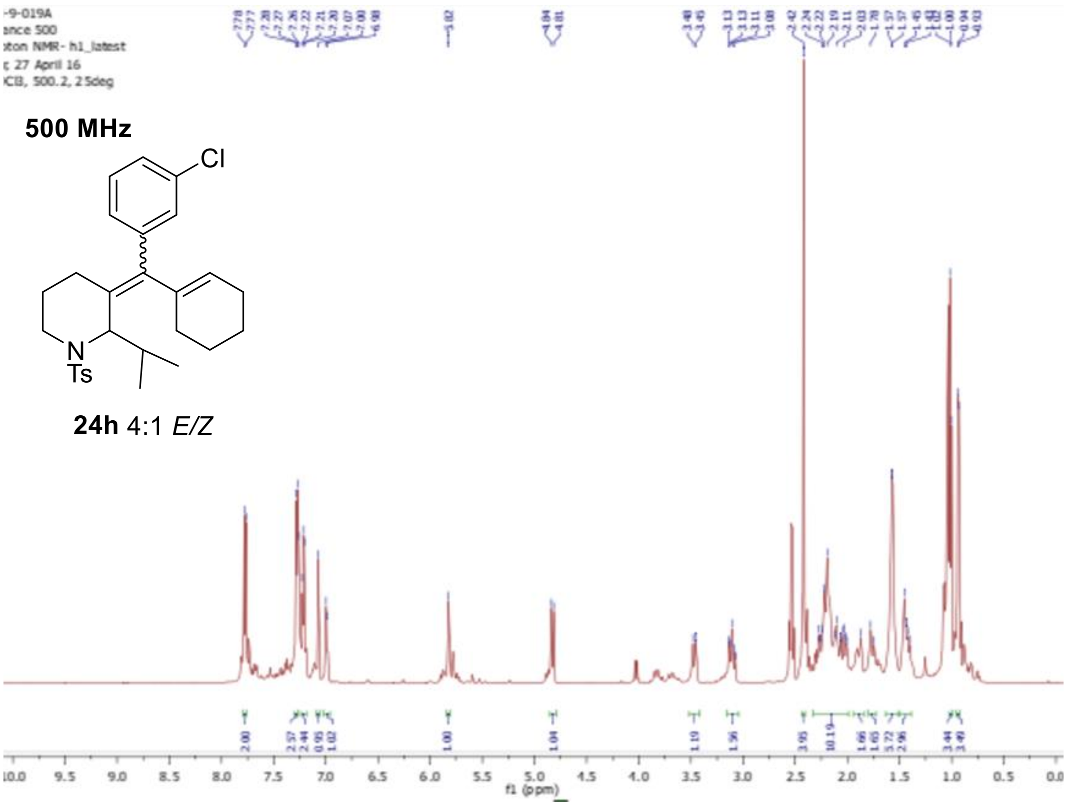

500 MHz
Cl
N
Ts
24h 4:1 *E/Z*
f1 (ppm)

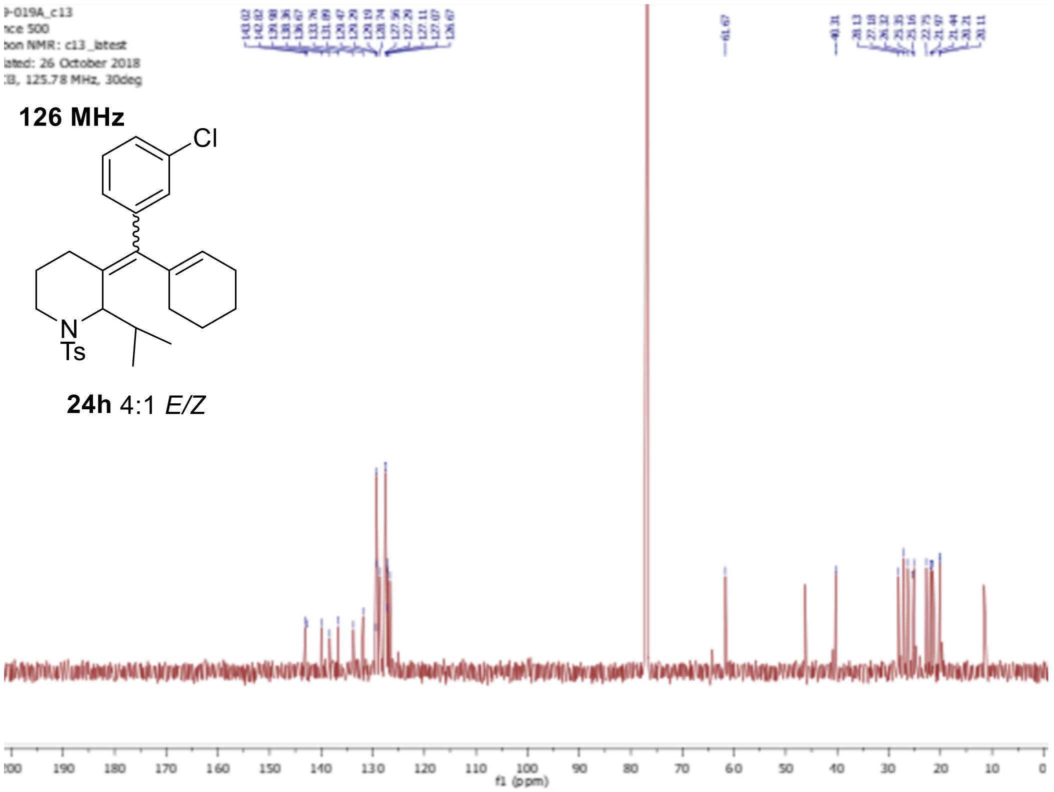

126 MHz
Cl
N
Ts
24h 4:1 *E/Z*
f1 (ppm)

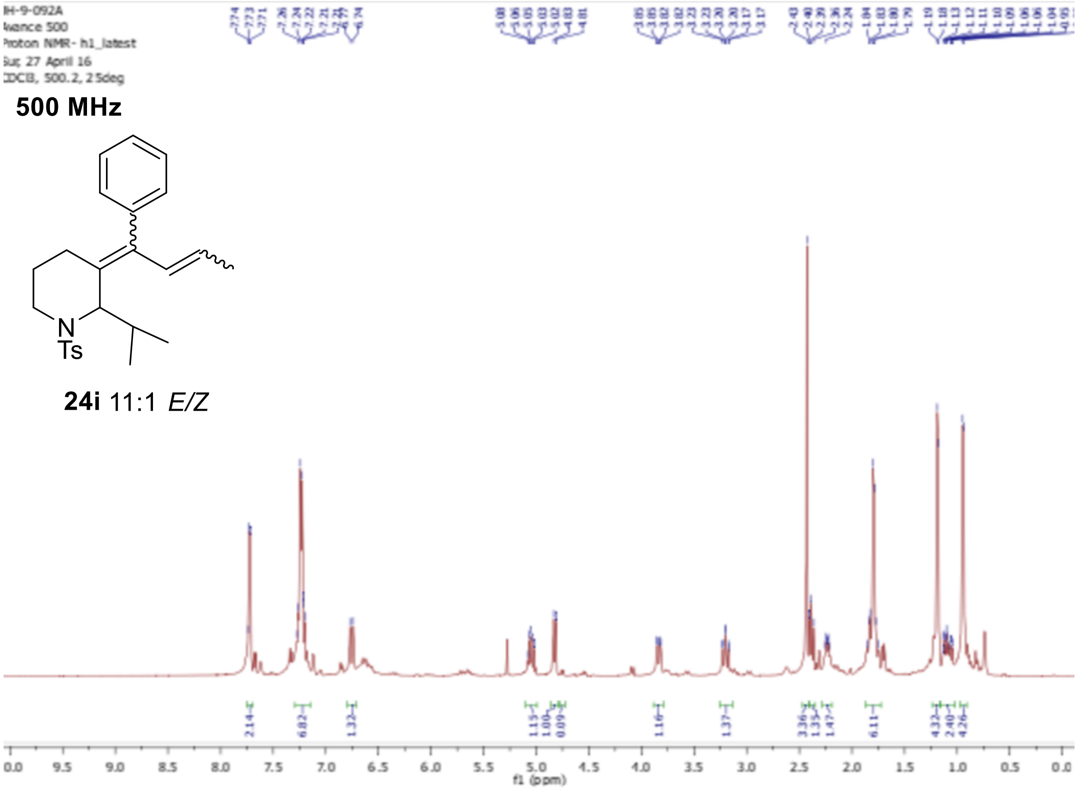
500 MHz
24i 11:1 E/Z
f1 (ppm)

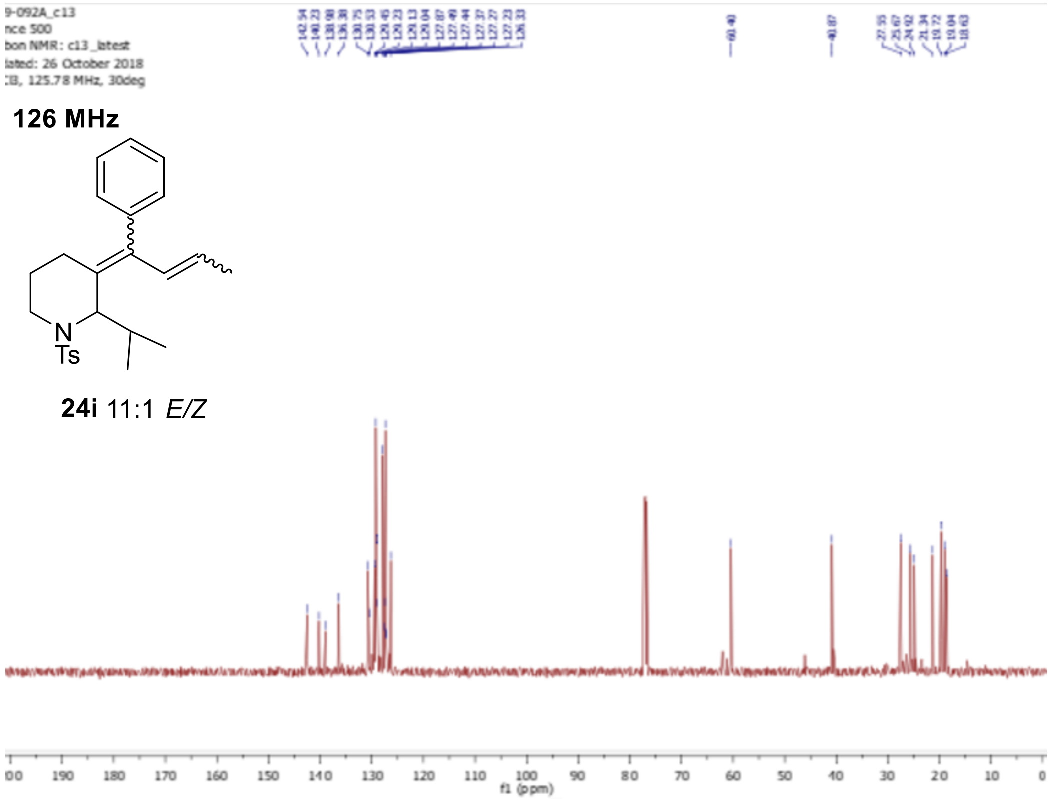
126 MHz
24i 11:1 E/Z
f1 (ppm)

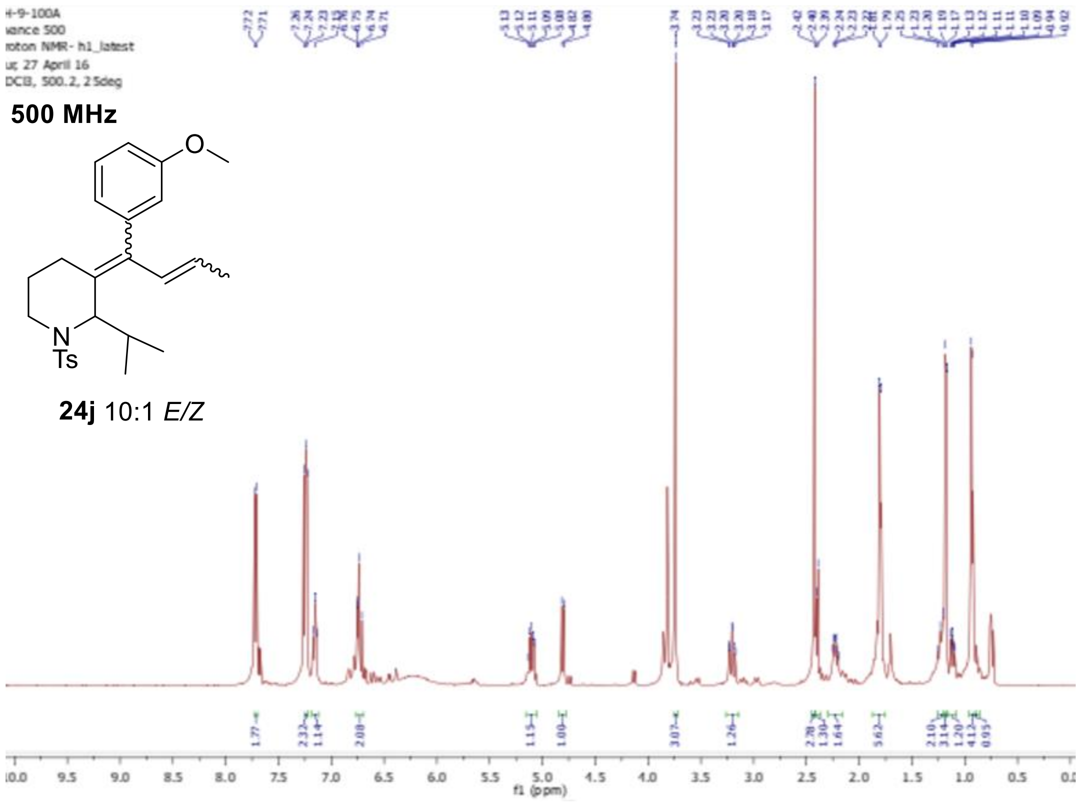

24j 10:1 *E/Z*

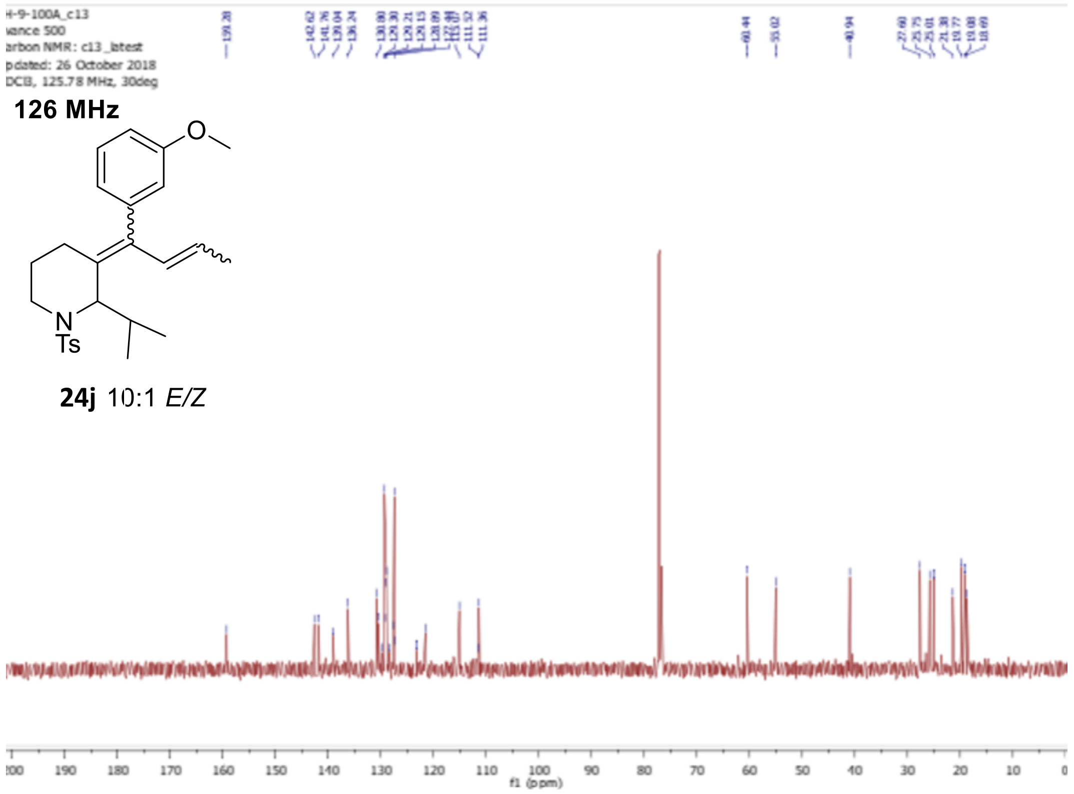

24j 10:1 *E/Z*
